# Causal roles of circulating cytokines in sarcopenia-related traits: a Mendelian randomization study

**DOI:** 10.3389/fendo.2024.1370985

**Published:** 2024-09-13

**Authors:** Zhi Chen, Jun Sun, Tengbin Shi, Chenyang Song, Chengjian Wu, Zhengru Wu, Jiajun Lin

**Affiliations:** ^1^ Department of Orthopedics, Fujian Medical University Union Hospital, Fuzhou, Fujian, China; ^2^ Department of Emergency, Zhaotong Traditional Chinese Medicine Hospital, Zhaotong, Yunnan, China

**Keywords:** inflammation, cytokines, sarcopenia, causal analysis, Mendelian randomization

## Abstract

**Background:**

Epidemiological and experimental evidence suggests that chronic inflammation plays an important role in the onset and progression of sarcopenia. However, there is inconsistent data on the inflammatory cytokines involved in the pathogenesis of sarcopenia. Therefore, we performed a two-sample Mendelian randomization (MR) analysis to explore the causal relationship between circulating cytokines and sarcopenia-related traits.

**Methods:**

The MR analysis utilized genetic data from genome-wide association study that included genetic variations in 41 circulating cytokines and genetic variant data for appendicular lean mass (ALM), hand grip strength, and usual walking pace. Causal associations were primarily explored using the inverse variance-weighted (IVW) method, supplemented by MR-Egger, simple mode, weighted median, and weighted mode analyses. Additionally, sensitivity analyses were also performed to ensure the reliability and stability of the results.

**Results:**

Three cytokines [hepatocyte growth factor (HGF), interferon gamma-induced protein 10 (IP-10), and macrophage colony-stimulating factor (M-CSF)] were positively associated with ALM (β: 0.0221, 95% confidence interval (CI): 0.0071, 0.0372, *P*= 0.0039 for HGF; β: 0.0096, 95%CI: 4e-04, 0.0189, *P*= 0.0419 for IP-10; and β: 0.0100, 95%CI: 0.0035, 0.0165, *P*= 0.0025 for M-CSF). Conversely, higher levels of interleukin-7 (IL-7), monocyte chemotactic protein 3 (MCP-3), and regulated on activation, normal T cell expressed and secreted (RANTES) were associated with decreased hand grip strength (β: -0.0071, 95%CI: -0.0127, -0.0014, *P*= 0.0140 for IL-7; β: -0.0064, 95%CI: -0.0123, -6e-04, *P*= 0.0313 for MCP-3; and β: -0.0082, 95%CI: -0.0164, -1e-04, *P*= 0.0480 for RANTES). Similarly, interleukin 1 receptor antagonist (IL-1RA) was negatively correlated with usual walking pace (β: -0.0104, 95%CI: -0.0195, -0.0013, *P*= 0.0254). Sensitivity analysis confirmed the robustness of these findings.

**Conclusions:**

Our study provides additional insights into the pivotal role of specific inflammatory cytokines in the pathogenesis of sarcopenia. Further research is required to determine whether these cytokines can be used as targets for the prevention and treatment of sarcopenia.

## Background

Sarcopenia is an age-related disease characterized by gradual loss of muscle mass, strength, and function, leading to an increased risk of falls, fractures, hospitalization, and death (1, 2). Owing to population aging, the incidence of sarcopenia is increasing annually by 10–27% worldwide (3). With the increasing prevalence, the pathogenesis and therapeutic targets of sarcopenia have emerged as research hotspots. An increasing number of studies indicated that a condition known as ‘inflammaging’, which was a sterile, chronic, low-grade systemic inflammatory state, might be closely related to the occurrence and progression of sarcopenia (4, 5).

Inflammation serves as a crucial protective and defensive mechanism within the body. Nonetheless, chronic local and systemic inflammation may result in the dysfuntion of immune cells, a perturbation in the balance between pro-inflammatory and anti-inflammatory cytokines, heightened oxidative stress, and disruptions in cellular metabolism, potentially culminating in apoptosis (6, 7). However, there have been inconsistent reports about the inflammatory cytokines identified to be involved in the pathogenesis of sarcopenia. In the ENHANce study, Dupont et al. (8) observed lower levels of IL-6 in patients with sarcopenia, while in another study, Rong et al. (9) reported increased IL-6 levels in elderly subjects with sarcopenia. Several studies revealed that sarcopenia risk increases with circulating cytokines, such as CRP, IL-10, growth differentiation factor-15, and TNF-α (9, 10). A meta-analysis revealed a significant correlation between elevated levels of CRP, IL-6, and TNF-α and reduced hand grip and knee extension strength (11). However, a discordance exists, as some studies have reported no significant association between CRP, IL-2, IL-6, IL-10, TNF-α, and sarcopenia (4, 12). Existing studies, which have drawn contradictory conclusions, were either based on a limited sample size or only explored the correlation between a few inflammatory markers and sarcopenia and the observational study design might be affected by confounding factors and reverse causality (13). Thus, we conducted a Mendelian randomization (MR) study to investigate the causal relationship between circulating cytokines levels and sarcopenia-related traits.

## Materials and methods

### Study design

**Figure 1:**
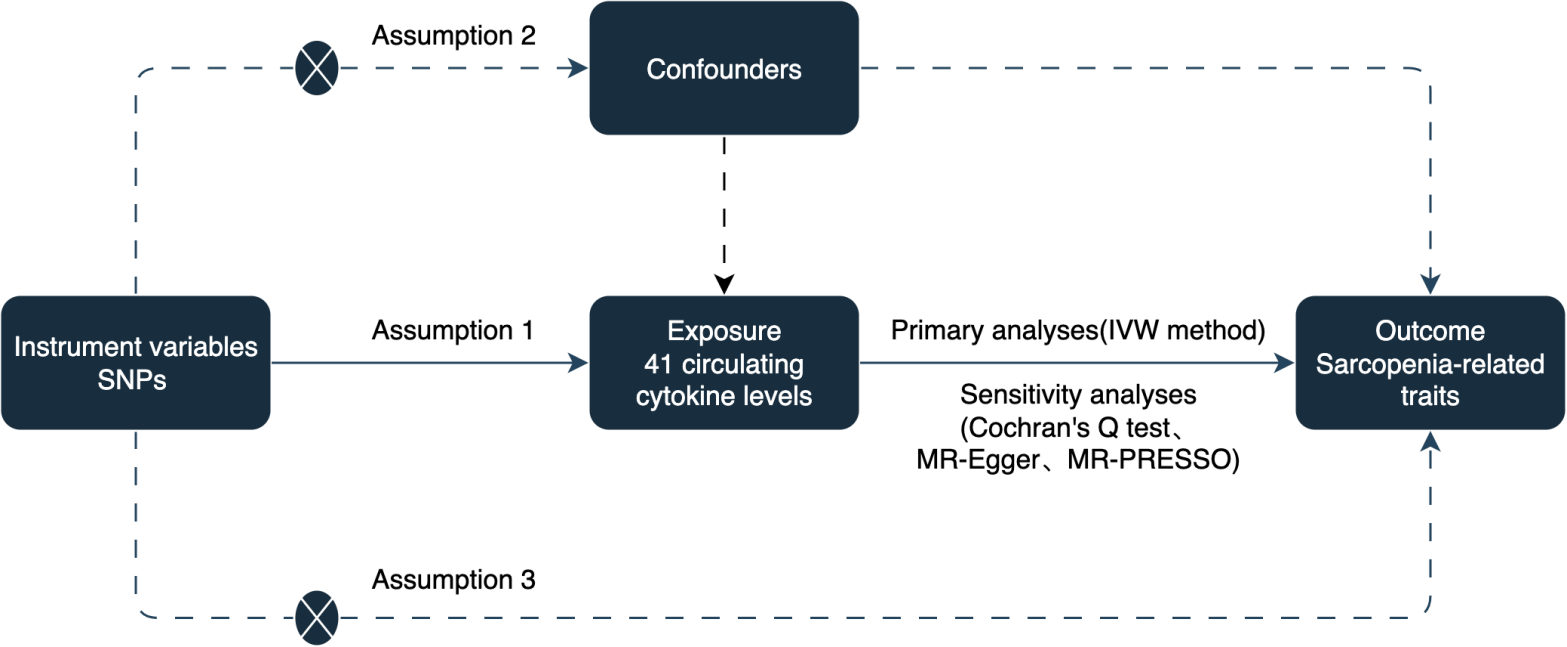
The schematic diagram of the present.

To establish a potential causal link between circulating cytokines and sarcopenia-related traits, including appendicular lean mass (ALM), hand grip strength, and usual walking pace, we conducted a two-sample MR study. The analysis was based on three assumptions: (1) instrumental variables (IVs) are strongly associated with circulating cytokines, (2) IVs are not associated with confounding factors, and (3) IVs only affect sarcopenia-related traits via circulating cytokines (14). The study design is illustrated in **Figure 1**.

### Data sources

The summary statistics and IVs for circulating levels of 41 cytokines were derived from a genome-wide association study (GWAS) that comprised 8293 European participants (15). Three GWAS summary statistics for sarcopenia-related traits [ALM (n=450243), hand grip strength (n=461089), and usual walking pace (n=459915)] were extracted from UK Biobank. These GWAS data can be downloaded from the IEU Open GWAS project (https://gwas.mrcieu.ac.uk/); detailed information is provided in **Table 1**.

**Table 1:** Details of the genome-wide association studies and datasets used in this study.

Exposure or outcome	Sample size	Ancestry	Links for data	PMID
Circulating concentrations of 41 cytokines	8,293 participants	European ancestry	https://data.bris.ac.uk/data/dataset?q=cytokines&level=top	27989323
Appendicular lean mass	450243 participants	European ancestry	http://gwas.mrcieu.ac.uk/datasets/ebi-a-GCST90000025/	33097823
Hand grip strength (right)	461089 participants	European ancestry	http://gwas.mrcieu.ac.uk/datasets/ukb-b-10215/	NA
Usual walking pace	459915 participants	European ancestry	http://gwas.mrcieu.ac.uk/datasets/ukb-b-4711/	NA

### Extraction of IVs

Based on the GWAS summary data for circulating cytokines, several quality control steps were performed to select eligible IVs. First, we extracted SNPs associated with circulating cytokines with genome-wide significance (P < 5 × 10^−6^). Second, to ensure that potential SNPs for circulating cytokines were not in linkage disequilibrium (LD), the threshold was set to r^2^ = 0.001 within a distance of 10000 kb (16). Finally, the F-statistics were calculated after harmonization. IVs with F-statistics >10 were considered sufficiently strong to mitigate the effects of potential bias (17).

### MR analysis

Five statistical methods were applied to obtain reliable results: MR-Egger, weighted median, inverse variance weighting (IVW), simple mode, and weighted mode (18). In cases where significant heterogeneity was observed, a random-effects method was performed; while, in other cases, a fixed-effects model was used. The MR estimates are presented as beta values (β) and corresponding 95% confidence intervals (CIs), with *P*-values < 0.05 considered statistically significant.

### Sensitivity analyses

To detect potential heterogeneity, we performed Cochran’s Q test and visualized funnel plots, with a *P*-value < 0.05 as the threshold of significance. Further, MR-Egger regression was performed and scatter plots were prepared, and an intercept term *P* -value < 0.05 was considered to have pleiotropy effects. Finally, we performed the Mendelian Randomization Pleiotropy Residual Sum and Outlier (MR-PRESSO) test to detect and remove outliers, and retested the results (19).

### Software

All statistical analyses were conducted using “Two-Sample MR,” “Mendelian Randomization,” and “MR-PRESSO” packages in the R statistical software (Version 4.1.2).

## Results

### Circulating cytokines and ALM

**Figure 2:**
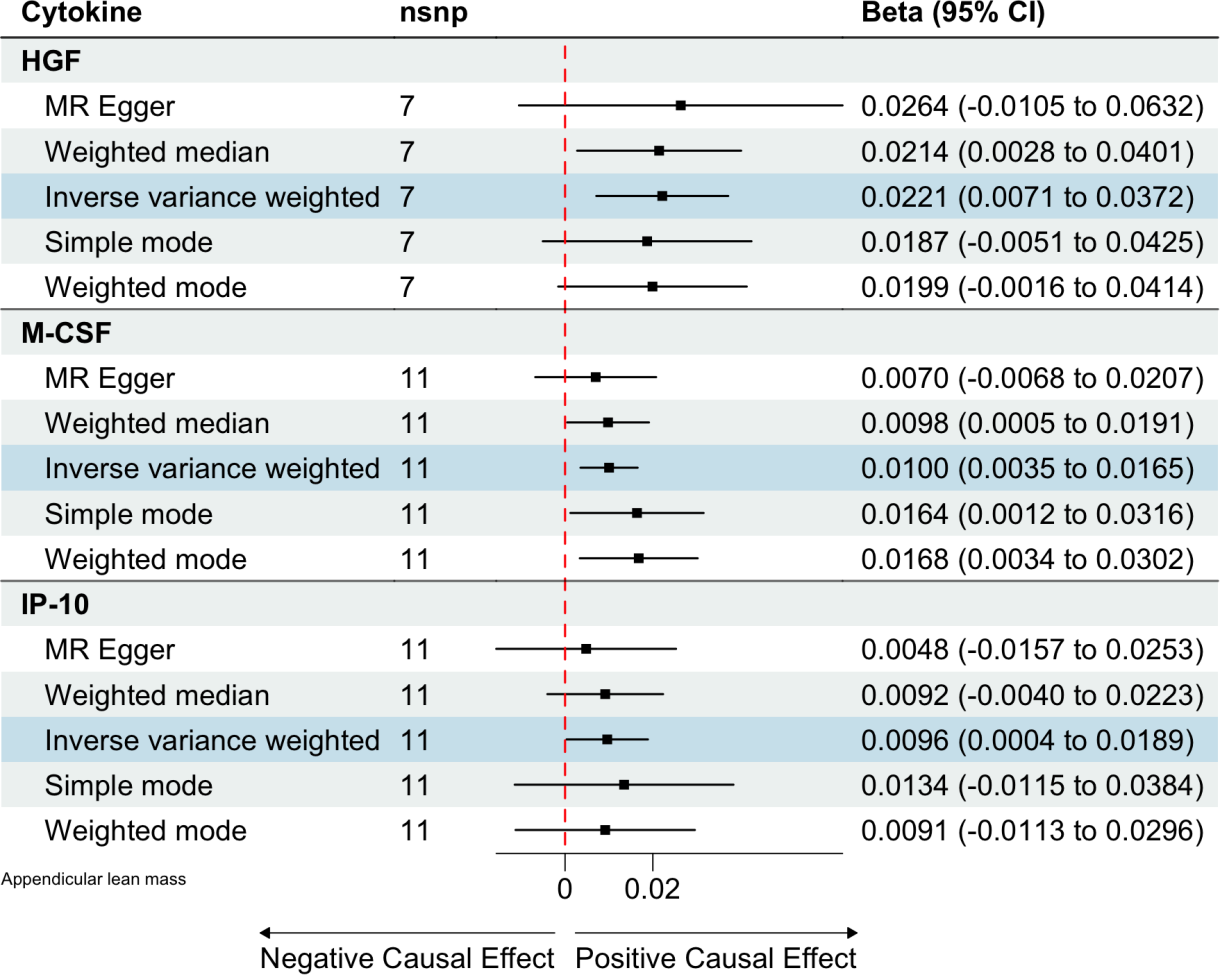
Estimated causal relationships of circulating cytokines with ALM with different MR methods.

Based on the aforementioned quality control measures, 413 SNPs were identified as IVs to investigate the causal relationships between circulating cytokines and ALM (**Supplementary Table 1**). The MR analysis showed that three cytokines, namely hepatocyte growth factor (HGF), interferon gamma-induced protein 10 (IP-10), and macrophage colony-stimulating factor (M-CSF), were causally associated with ALM in the IVW method. All of these cytokines had positive effects on ALM (β: 0.0221, 95%CI: 0.0071, 0.0372, *P*= 0.0039 for HGF; β: 0.0096, 95%CI: 4e-04, 0.0189, *P*= 0.0419 for IP-10; and β: 0.0100, 95%CI: 0.0035, 0.0165, *P*= 0.0025 for M-CSF) (**Table 2**; **Figure 2**). The Cochran’s Q test revealed no significant results, confirming the stability of the major findings. In addition, the MR-Egger intercept and MR-PRESSO tests detected non-significant results, indicating that the main estimates were less likely to be affected by horizontal pleiotropy (**Table 3**; **Supplementary Figures 1**-**2**).

**Table 2:** MR estimates from different methods of assessing the causal effect of circulating cytokines on ALM.

Outcome	Exposure	No. of IVs	Method	Beta (95% CI)	*P*-value
ALM	HGF	7	IVW	0.0221 (0.0071,0.0372)	0.0039
			MR Egger	0.0264 (-0.0105,0.0632)	0.2200
			Weighted median	0.0214 (0.0021,0.0408)	0.0298
			Simple mode	0.0187 (-0.0076,0.045)	0.2122
			Weighted mode	0.0199 (0,0.0399)	0.0982
ALM	IP-10	11	IVW	0.0096 (4e-04,0.0189)	0.0419
			MR Egger	0.0048 (-0.0157,0.0253)	0.6566
			Weighted median	0.0092 (-0.0045,0.0228)	0.1886
			Simple mode	0.0134 (-0.0101,0.037)	0.2893
			Weighted mode	0.0091 (-0.0113,0.0295)	0.4001
ALM	M-CSF	11	IVW	0.0100 (0.0035,0.0165)	0.0025
			MR Egger	0.007 (-0.0068,0.0207)	0.3463
			Weighted median	0.0098 (0.0011,0.0184)	0.0266
			Simple mode	0.0164 (0.0016,0.0312)	0.0547
			Weighted mode	0.0168 (0.0025,0.031)	0.0435

**Table 3:** The results of heterogeneity and pleiotropy test for MR analysis of the causal relationships between circulating cytokines and sarcopenia-related traits.

Outcome	Exposure	No. of IVs	Cochran's Q		MR-Egger		MR-PRESSO	
			Q	P-value	Intercept	P-value	RSSobs	P-value
ALM	HGF	7	6.5239	0.26	-7.06E-04	0.81	7.5419	0.5095
	IP-10	11	10.4536	0.32	1.04E-03	0.61	12.9874	0.4044
	M-CSF	11	8.1407	0.52	1.13E-03	0.63	10.1443	0.6291
Hand grip strength	IL-7	10	7.3636	0.50	7.20E-04	0.67	9.6650	0.6007
	MCP-3	6	3.2493	0.52	4.53E-04	0.84	4.6747	0.6947
	RANTES	9	10.1903	0.18	-1.13E-03	0.68	13.4386	0.2481
Usual walking pace	IL-1RA	7	4.4102	0.49	-3.80E-03	0.10	12.3688	0.2247

### Circulating cytokines and hand grip strength

**Figure 3:**
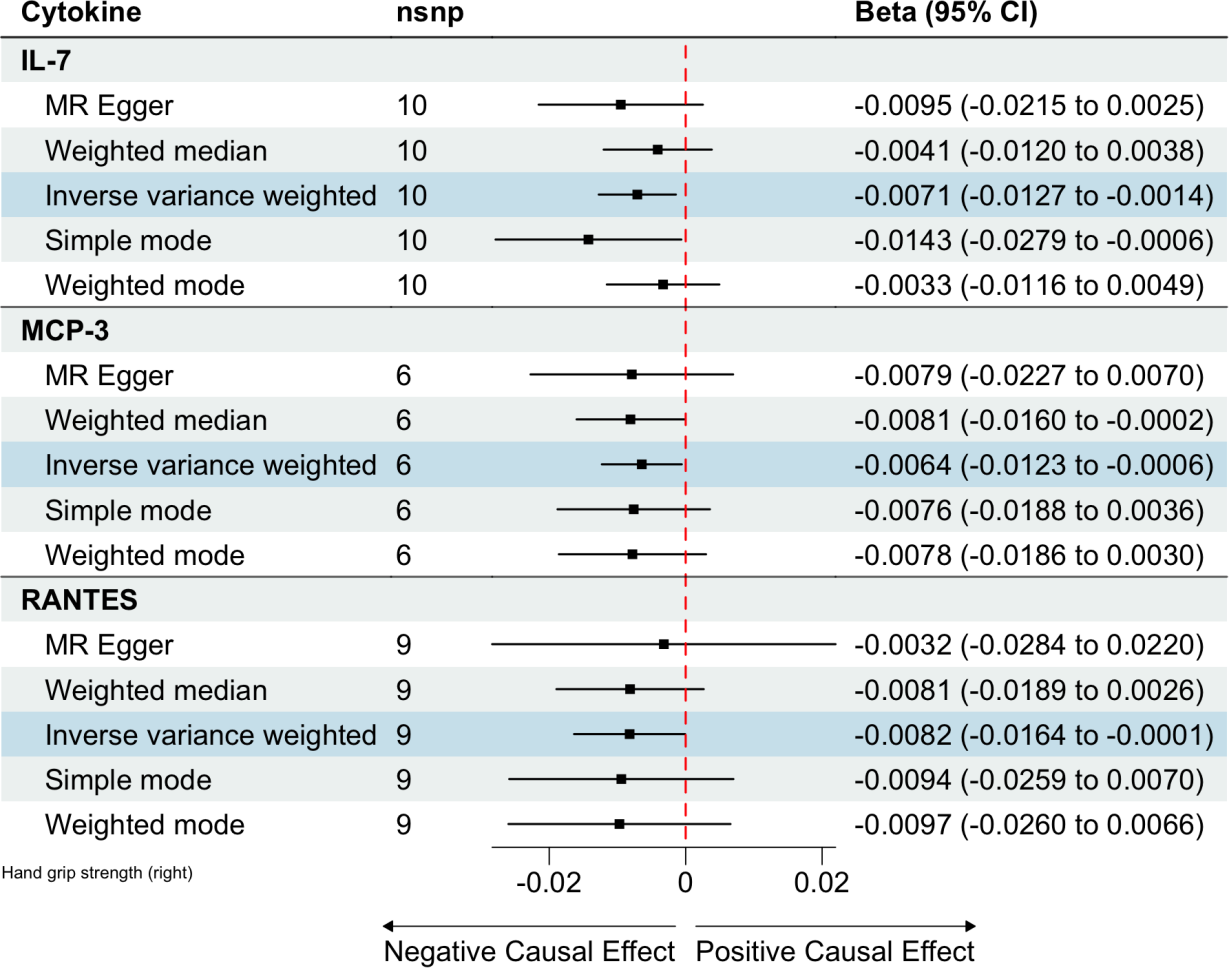
Estimated causal relationships of circulating cytokines with hand grip strength with different MR methods.

In total, 404 SNPs were identified as IVs to explore the causal relationships between circulating cytokines and hand grip strength (**Supplementary Table 2**). MR analysis using the IVW method revealed that three cytokines, namely interleukin-7 (IL-7), monocyte chemotactic protein 3 (MCP-3), and regulated on activation, normal T cell expressed and secreted (RANTES), were causally associated with hand grip strength. These cytokines had negative effects on hand grip strength (β: -0.0071, 95%CI: -0.0127, -0.0014, *P*= 0.0140 for IL-7; β: -0.0064, 95%CI: -0.0123, -6e-04, *P*= 0.0313 for MCP-3; and β: -0.0082, 95%CI: -0.0164, -1e-04, *P*= 0.0480 for RANTES) (**Table 4**; **Figure 3**). The Cochran’s Q test revealed no significant heterogeneity. The results of the MR-Egger intercept and MR-PRESSO tests demonstrated that the horizontal pleiotropy did not bias the causal effect of cytokines on hand grip strength (**Table 3**; **Supplementary Figures 3**, **4**).

**Table 4:** MR estimates from different methods of assessing the causal effect of circulating cytokines on hand grip strength.

Outcome	Exposure	No. of IVs	Method	Beta (95% CI)	P-value
Hand grip strength	IL-7	10	IVW	-0.0071 (-0.0127, -0.0014)	0.0140
			MR Egger	-0.0095 (-0.0215, 0.0025)	0.1599
			Weighted median	-0.0041 (-0.012, 0.0038)	0.3109
			Simple mode	-0.0143 (-0.0279, -6e-04)	0.0704
			Weighted mode	-0.0033 (-0.0116, 0.0049)	0.4518
Hand grip strength	MCP-3	6	IVW	-0.0064 (-0.0123, -6e-04)	0.0313
			MR Egger	-0.0079 (-0.0227, 0.007)	0.3563
			Weighted median	-0.0081 (-0.016, -2e-04)	0.0445
			Simple mode	-0.0076 (-0.0188, 0.0036)	0.2391
			Weighted mode	-0.0078 (-0.0186, 0.003)	0.2154
Hand grip strength	RANTES	9	IVW	-0.0082 (-0.0164, -1e-04)	0.0480
			MR Egger	-0.0032 (-0.0284, 0.022)	0.8105
			Weighted median	-0.0081 (-0.0189, 0.0026)	0.1388
			Simple mode	-0.0094 (-0.0259, 0.007)	0.2932
			Weighted mode	-0.0097 (-0.026, 0.0066)	0.2760

### Circulating cytokines and usual walking pace

**Figure 4:**
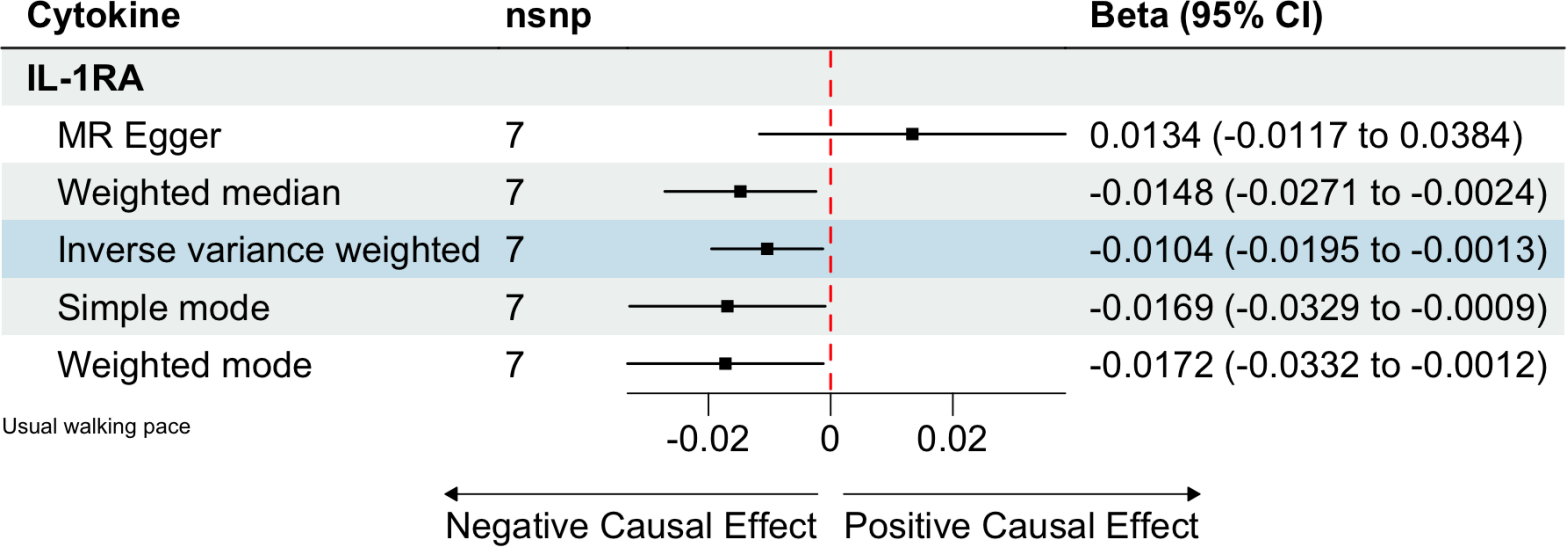
Estimated causal relationships of circulating cytokines with usual walking pace with different MR methods.

We identified 408 eligible SNPs to investigate the causal associations between circulating cytokines and the usual walking pace (**Supplementary Table 3**). Interleukin 1 receptor antagonist (IL-1RA) revealed significant negative causal effects on usual walking pace (β: -0.0104, 95%CI: -0.0195, -0.0013, *P*= 0.0254, IVW method) (**Table 5**; **Figure 4**). Cochran’s Q test did not show any significant heterogeneity. No evidence of horizontal pleiotropy was observed based on the MR-Egger intercept and MR-PRESSO test (**Table 3**; **Supplementary Figures 5**, **6**).

**Table 5:** MR estimates from different methods of assessing the causal effect of circulating cytokines on usual walking pace.

Outcome	Exposure	No. of IVs	Method	Beta (95% CI)	P-value
Usual walking pace	IL-1RA	7	IVW	-0.0104 (-0.0195, -0.0013)	0.0254
			MR Egger	0.0134 (-0.0117, 0.0384)	0.3432
			Weighted median	-0.0148 (-0.027, -0.0025)	0.0179
			Simple mode	-0.0169 (-0.0335, -3e-04)	0.0934
			Weighted mode	-0.0172 (-0.0328, -0.0017)	0.0731

## Discussion

Recently, the influence of inflammatory processes on the initiation and progression of sarcopenia has become a focal point of interest within the scientific community. However, prior research has been hindered by limitations such as restricted sample sizes or focusing solely on the relationship between a limited set of inflammatory biomarkers and sarcopenia. Moreover, the observational study designs previously utilized may be prone to confounding factors and the complexities of reverse causality, which have collectively led to inconsistencies in the findings. Our Mendelian randomization study provides novel insights, indicating that sarcopenia is not triggered by a single cytokine but rather is the result of an imbalance within the intricate cytokine regulatory network. The perturbation of the delicate equilibrium between pro-inflammatory and anti-inflammatory cytokines, when disrupted, may significantly impact the pathogenesis and clinical course of sarcopenia.

Emerging evidence suggests that the proliferation and differentiation of muscle satellite cells are influenced by growth factors and hormones, including hepatocyte growth factor (HGF), insulin-like growth factors-1 (IGF-1), and testosterone (20). An *in vivo* study showed that increasing HGF levels improved age-related muscle regeneration and dysfunction (21). HGF has been suggested to promote skeletal muscle regeneration by regulating the mobilization and modification of bone marrow stem cells (22, 23). In addition to these experimental studies, a few clinical observational studies have proposed a relationship between sarcopenia and HGF (24, 25). Our MR analysis have revealed a protective role of HGF against muscle loss. IP-10, also known as C-X-C motif ligand 10 (CXCL-10), is a potent monocyte or dendritic cell-derived chemokine involved in T-cell migration and activation (26). Recently, the role of IP-10 in muscle disorders has gained the attention of researchers. Deyhle et al. (27) observed that CXCL-10 promoted the myogenic differentiation of human primary myoblasts *in vitro*, suggesting its involvement in muscle regeneration. However, clinical studies have reported conflicting results. A cohort study analyzing the relationship between skeletal muscle index (SMI) and 39 circulating cytokines in 125 patients with colon cancer, reported that an increased IP-10 level correlated significantly with a lower SMI (28). Contrarily, another study revealed a significantly lower IP-10 in older men than that in their younger counterparts (29). Similarly, Perrini et al. (30) reported that IP-10 levels decreased with age and were associated with muscle decline. Although we observed a positive association between IP-10 and ALM, further research is required to clarify the underlying mechanisms. M-CSF is a well-known monocyte mobilizer that regulates the function of various inflammatory cells and induces the survival, proliferation, and maturation of macrophages (31). M-CSF has been used successfully for muscle recovery in several mouse injury models (32, 33). Some researchers have found that aged muscle treated with M-CSF had higher macrophage content and greater muscle force (34). Consistent with these experimental findings, our results suggest a protective effect of M-CSF against ALM. Our findings implicate HGF, IP-10, and M-CSF as promising candidates for biomarkers and therapeutic targets in the context of muscle wasting. Further research is essential to elucidate the precise mechanisms of action and the intricate regulatory networks within which these cytokines operate. This understanding is critical for the advancement of targeted interventions to ameliorate sarcopenia more effectively.

It has been proposed that higher levels of circulating cytokines may play a role in muscle strength decline (11). IL-7 is a cytokine chiefly produced by the skeletal muscle (29). *In vitro* and *in vivo* experiments have shown that IL-7 affects the myogenesis and migration of skeletal muscle cells, suggesting the physiological importance of muscular IL-7 (35). In a cohort study, Weerd et al. (36) found that morbidly obese participants had higher serum IL-7 levels than the lean healthy participants. In another study investigating the effects of exercise on circulating IL-7 levels, Andersson et al. (37) observed significantly elevated circulating IL-7 levels immediately after soccer games. Our findings suggest a negative causal relationship between IL-7 levels and hand grip strength. IL-7 is likely to have dual effects on muscle strength depending on the duration of exposure. When increased acutely, it may positively affect muscle metabolism by upregulating lipolysis and fat oxidation; however, it may reduce muscle strength when increased chronically, as is the case in inflammation (38). Monocyte chemotactic proteins (MCPs) are chemotactic cytokines that regulate a distinct spectrum of target cells and exhibit different biological activities depending on the cell type (39). *In vivo* experimental data have disclosed a pronounced elevation in the expression of MCP-1 within the skeletal muscle tissue of SAMP8 mice, correlating with their advancement in age from 12 to 40 weeks (40). In a clinical study, an age-related increase in the levels of MCP-1 has been observed among healthy individuals (41). Currently, five members of the MCP family have been identified: MCP-1, MCP-2, MCP-3, MCP-4, and MCP-5 (39). Several studies have reported that MCP-1 impairs muscle anabolism and accelerates catabolism, leading to impaired muscle strength (42, 43). Our MR analysis revealed a negative causal relationship between MCP-3 and muscle strength, suggesting the chemotactic effects of MCP-3 are likely broader than those of MCP-1 (39). RANTES contributes to the aggregation of inflammatory cells and to persistent inflammatory response (44). RANTES has been implicated as a key modifier of the immune response in muscle-wasting diseases, such as Duchenne muscular dystrophy (45). *In vivo* experiments have also revealed the accumulation of RANTES in the skeletal muscles of sarcopenic rats (44). In a cross-sectional study, Fielding et al. (46) observed a negative association between RANTES and physical function in females. Our MR analysis further confirmed the negative causal relationship between RANTES levels and muscle strength.

IL-1RA is a member of the IL-1 cytokine family and is secreted by various of cells. IL-1RA is assumed to exert anti-inflammatory effects by antagonizing the IL-1 pathway via binding to the IL-1 receptor (47). However, the role of IL-1RA in the inflammation of skeletal muscles remains controversial. The finding from an animal study indicated a modest rise in serum IL-1RA levels among rats engaged in moderate and intense physical activity, as opposed to their sedentary counterparts (48). Another study elucidated that IL-1RA levels were lower in white adipose tissue, higher in skeletal muscle, and similar in the serum of trained rats compared to those in sedentary rats (49). The outcomes of a placebo-controlled, double-blinded, parallel-group clinical trial highlighted that IL-1RA therapy exerted no significant influence on gene expression within the skeletal muscle of obese individuals suffering from type 2 diabetes (50). In a cross-sectional study, Cesari et al. (51) reported a statistically significant correlation linking elevated IL-1RA levels to diminished physical performance and reduced muscular strength in older adults residing in the community. Similarly, our MR analysis indicated a negative causal relationship between IL-1RA and usual walking pace. Owing to the inconsistency in these findings, additional studies are required to determine the exact mechanisms of IL-1RA in the progression of sarcopenia.

## Limitations

This study had some limitations. First, the GWAS included only individuals of European ancestry, limiting the applicability of our findings to different ethnic backgrounds. Second, owing to the lack of individual-level data, a stratified analysis could not be performed to determine whether the causal effects of circulating cytokines on sarcopenia-related traits varied by age and sex. Third, residual bias is inevitable, as it is a recognized drawback of the MR technique, even with pleiotropic tests and MR-PRESSO procedures to prevent pleiotropic mixing. Therefore, further research is required to verify the association between these variables.

## Conclusion

In this study, we employed MR analysis to provide a more holistic perspective on the causal relationship between inflammatory cytokines and sarcopenia, suggesting that elevated levels of IL-7, MCP-3, RANTES, and IL-1RA increase, whereas HGF, IP-10, and M-CSF decrease the risk of sarcopenia. This study may shed new light on the etiology, diagnosis, prevention, and treatment of sarcopenia. However, further studies are needed to fully understand the precise biological mechanisms and determine the potential of these cytokines as targets for the prevention or treatment of sarcopenia.

## Data Availability

The original contributions presented in the study are included in the article/**Supplementary Material**. Further inquiries can be directed to the corresponding author.

## Supplementary material

Supplementary Figure 1 Causal relationships between circulating cytokines and ALM in funnel plots. **(A)**. HGF, **(B)**. IP-10, **(C)**. M-CSF.

Supplementary Figure 2 Causal relationships between circulating cytokines and ALM in scatter plots. **(A)**. HGF, **(B)**. IP-10, **(C)**. M-CSF.

Supplementary Figure 3 Causal relationships between circulating cytokines and hand grip strength in funnel plots. **(A)**. IL-7, **(B)**. MCP-3, **(C)**. RANTES.

Supplementary Figure 4 Causal relationships between circulating cytokines and hand grip strength in scatter plots. **(A)**. IL-7, **(B)**. MCP-3, **(C)**. RANTES.

Supplementary Figure 5 Causal relationships between circulating cytokines and usual walking pace in funnel plots. a.IL-1RA.

Supplementary Figure 6 Causal relationships between circulating cytokines and usual walking pace in scatter plots. a.IL-1RA.
