# Supplementary figures and images for: Causal roles of circulating cytokines in sarcopenia-related traits: a Mendelian randomization study
Source: Front Endocrinol (Lausanne). 2024 Sep 13;15:1370985. doi: 10.3389/fendo.2024.1370985 (PMC11427268; doi:10.3389/fendo.2024.1370985)

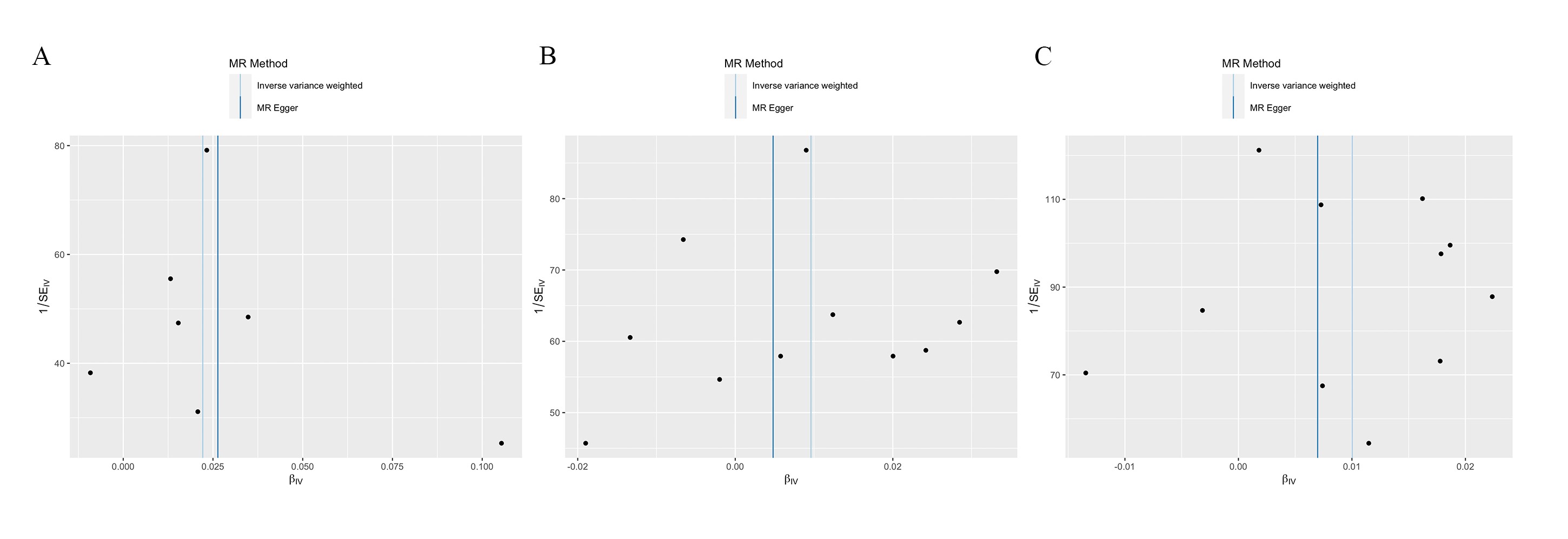

Supplement: Supplementary Figure 1 — Causal relationships between circulating cytokines and ALM in funnel plots. (A). HGF, (B). IP-10, (C). M-CSF. [file Image1.jpeg]

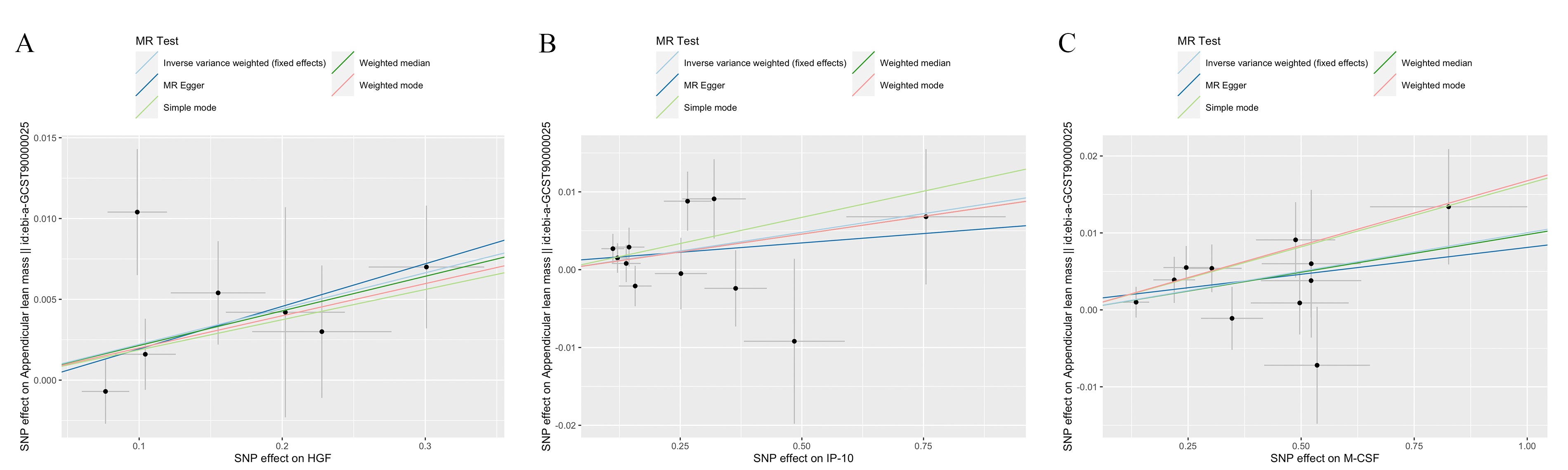

Supplement: Supplementary Figure 2 — Causal relationships between circulating cytokines and ALM in scatter plots. (A). HGF, (B). IP-10, (C). M-CSF. [file Image2.jpeg]

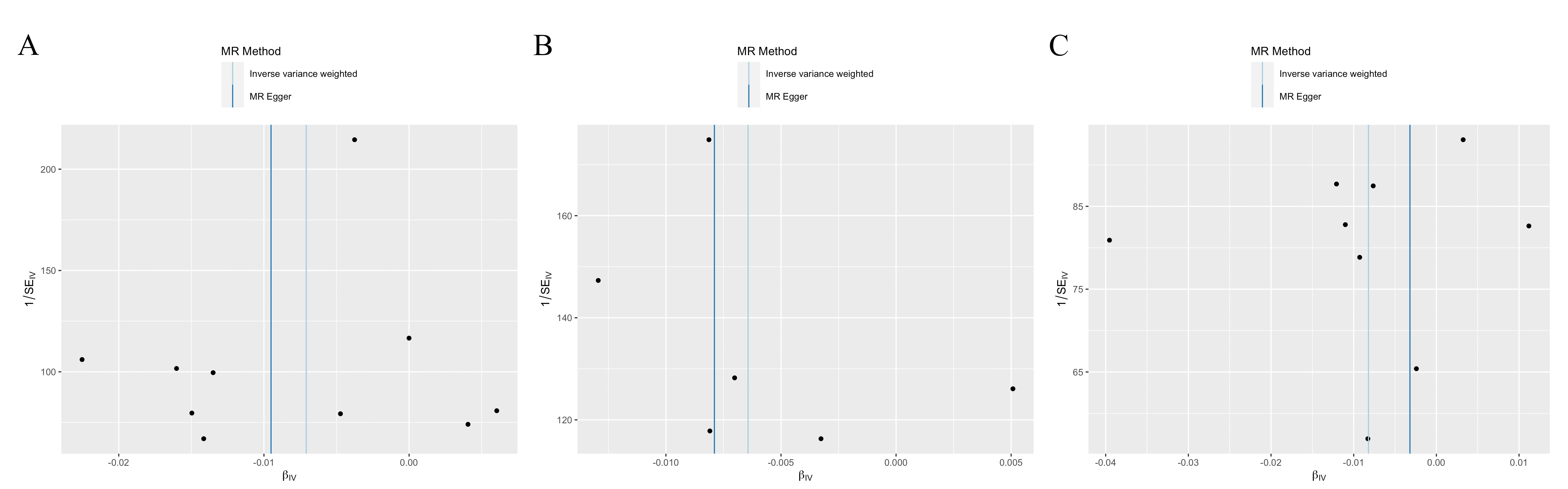

Supplement: Supplementary Figure 3 — Causal relationships between circulating cytokines and hand grip strength in funnel plots. (A). IL-7, (B). MCP-3, (C). RANTES. [file Image3.jpeg]

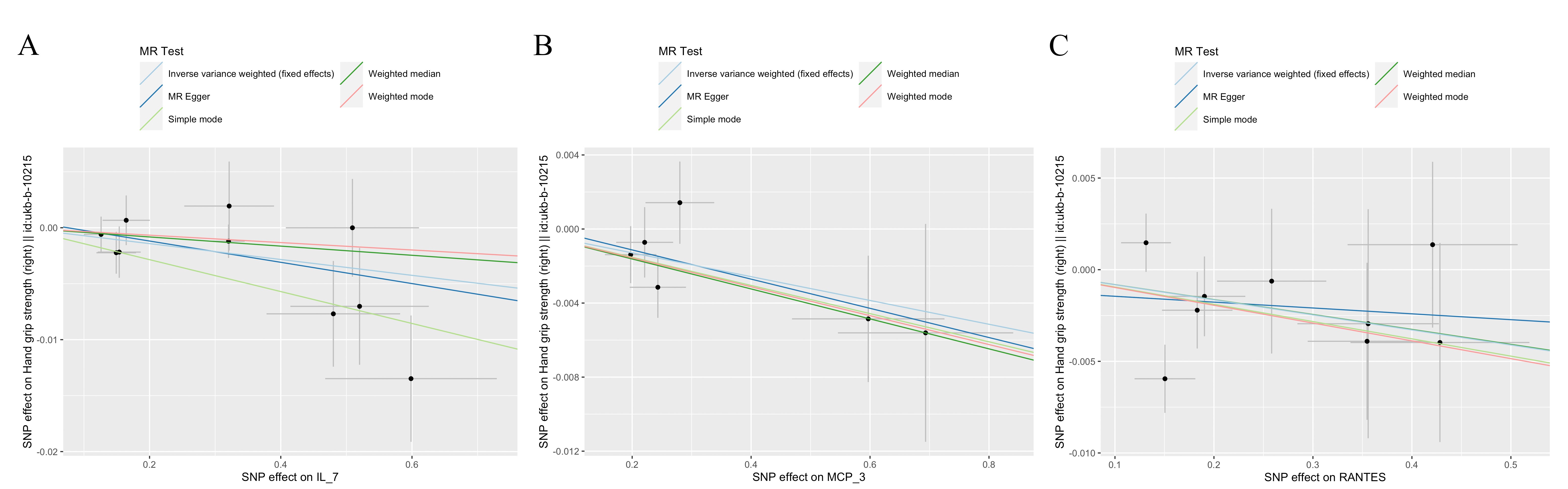

Supplement: Supplementary Figure 4 — Causal relationships between circulating cytokines and hand grip strength in scatter plots. (A). IL-7, (B). MCP-3, (C). RANTES. [file Image4.jpeg]

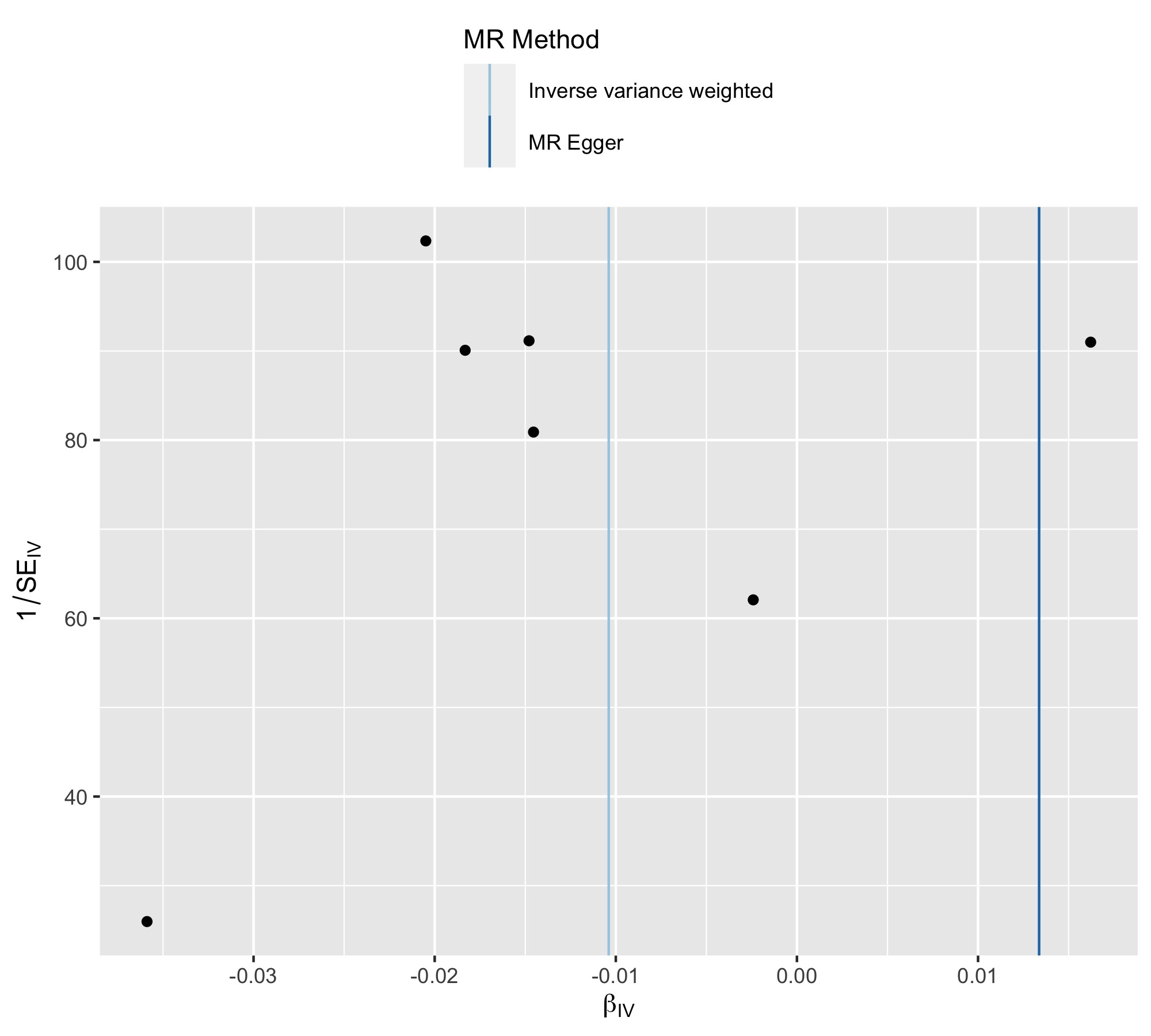

Supplement: Supplementary Figure 5 — Causal relationships between circulating cytokines and usual walking pace in funnel plots. a.IL-1RA. [file Image5.jpeg]

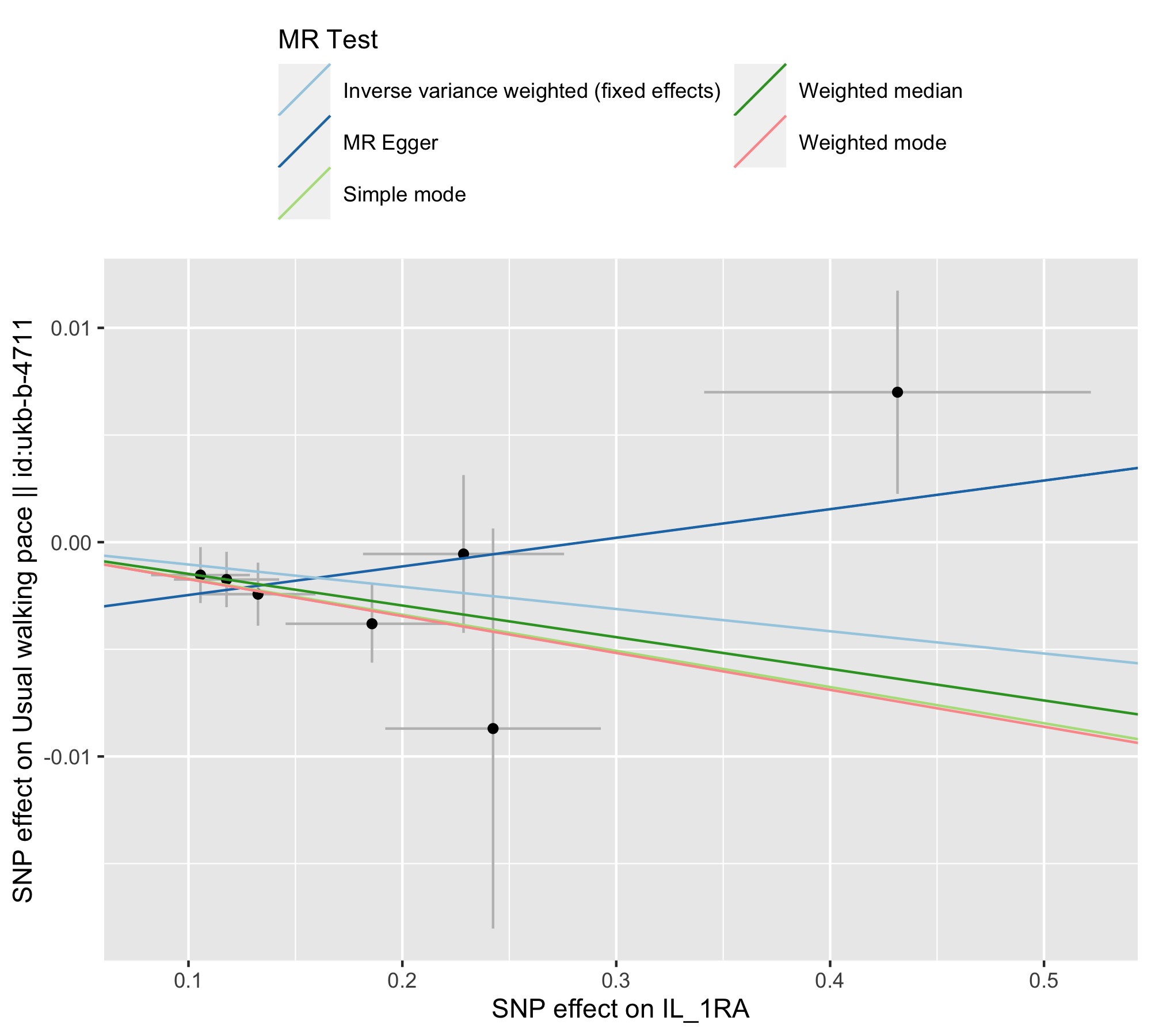

Supplement: Supplementary Figure 6 — Causal relationships between circulating cytokines and usual walking pace in scatter plots. a.IL-1RA. [file Image6.jpeg]
