# Supplementary material for: Causal roles of circulating cytokines in sarcopenia-related traits: a Mendelian randomization study
Source: Front Endocrinol (Lausanne). 2024 Sep 13;15:1370985. doi: 10.3389/fendo.2024.1370985 (PMC11427268; doi:10.3389/fendo.2024.1370985)
Supplement: Supplementary file 7 [file Table1.docx]

Supplemental table 1. Genome-wide significant SNPs used as IVs to investigate causal relationships between circulating cytokines and ALM.

| **Outcome** | **Exposure** | **SNP** | **Exposure** | | | **Outcome** | | |
| --- | --- | --- | --- | --- | --- | --- | --- | --- |
|  |  |  | **Beta** | **Se** | **P-value** | **Beta** | **Se** | **P-value** |
| ALM | B_NGF | rs28637706 | -0.1554 | 0.0261 | 2.72E-09 | 0.0038 | 0.002 | 0.05387 |
| ALM | B_NGF | rs73472576 | -0.1146 | 0.0251 | 4.81E-06 | 7.00E-04 | 0.002 | 0.717799 |
| ALM | B_NGF | rs7970581 | 0.1358 | 0.028 | 1.22E-06 | -0.0026 | 0.0022 | 0.2476 |
| ALM | CTACK | rs116303454 | 0.3754 | 0.081 | 3.58E-06 | -0.0101 | 0.0065 | 0.1185 |
| ALM | CTACK | rs118084576 | 0.5675 | 0.1226 | 3.66E-06 | 0.0114 | 0.0088 | 0.1918 |
| ALM | CTACK | rs135564 | -0.1672 | 0.0267 | 3.59E-10 | -0.0028 | 0.0021 | 0.1753 |
| ALM | CTACK | rs141331414 | 0.1977 | 0.0415 | 1.89E-06 | -0.008 | 0.0036 | 0.02727 |
| ALM | CTACK | rs2070074 | 0.4401 | 0.0372 | 2.60E-32 | 0.0031 | 0.0032 | 0.3307 |
| ALM | CTACK | rs55764737 | 0.5424 | 0.0967 | 2.01E-08 | -6.00E-04 | 0.0051 | 0.9028 |
| ALM | CTACK | rs57789542 | -0.7687 | 0.1659 | 3.58E-06 | 0.0153 | 0.0066 | 0.02011 |
| ALM | CTACK | rs60247384 | 0.1128 | 0.0245 | 4.30E-06 | -8.00E-04 | 0.0022 | 0.7165 |
| ALM | CTACK | rs62578137 | -0.1311 | 0.0286 | 4.66E-06 | -0.0049 | 0.0025 | 0.04524 |
| ALM | CTACK | rs72729450 | -0.5123 | 0.1094 | 2.81E-06 | 0.0039 | 0.0077 | 0.612999 |
| ALM | CTACK | rs7333764 | 0.2811 | 0.0591 | 2.00E-06 | 9.00E-04 | 0.0068 | 0.8999 |
| ALM | CTACK | rs76395525 | 0.5193 | 0.1081 | 1.55E-06 | -0.0064 | 0.0084 | 0.4466 |
| ALM | EOTAXIN | rs11087905 | 0.0954 | 0.0188 | 4.07E-07 | 0.004 | 0.0023 | 0.077999 |
| ALM | EOTAXIN | rs112347425 | 0.1595 | 0.0276 | 7.77E-09 | -0.0046 | 0.0032 | 0.1521 |
| ALM | EOTAXIN | rs12075 | 0.1692 | 0.0155 | 1.21E-27 | -5.00E-04 | 0.0019 | 0.7891 |
| ALM | EOTAXIN | rs187131 | 0.1264 | 0.0253 | 5.74E-07 | -0.0044 | 0.0028 | 0.1205 |
| ALM | EOTAXIN | rs2024050 | 0.164 | 0.0302 | 5.47E-08 | 0.0077 | 0.0031 | 0.01247 |
| ALM | EOTAXIN | rs2027855 | 0.0743 | 0.0162 | 4.27E-06 | 0.0027 | 0.002 | 0.1879 |
| ALM | EOTAXIN | rs2211994 | 0.0876 | 0.0177 | 6.98E-07 | -0.0034 | 0.0022 | 0.1204 |
| ALM | EOTAXIN | rs2228467 | -0.4154 | 0.0291 | 3.47E-46 | -0.0035 | 0.0039 | 0.374 |
| ALM | EOTAXIN | rs5754733 | -0.105 | 0.0213 | 8.20E-07 | -0.0017 | 0.0023 | 0.4439 |
| ALM | EOTAXIN | rs59808887 | -0.1698 | 0.0356 | 1.89E-06 | 0.0011 | 0.0035 | 0.749799 |
| ALM | EOTAXIN | rs745331 | -0.0821 | 0.0176 | 3.04E-06 | 9.00E-04 | 0.0021 | 0.677001 |
| ALM | EOTAXIN | rs75426604 | -0.1371 | 0.0291 | 2.40E-06 | 0.0062 | 0.0028 | 0.02821 |
| ALM | EOTAXIN | rs79722574 | -0.1092 | 0.0227 | 1.50E-06 | -0.0019 | 0.0026 | 0.4739 |
| ALM | EOTAXIN | rs9317045 | 0.1172 | 0.0236 | 6.95E-07 | -0.0019 | 0.0026 | 0.4591 |
| ALM | FGF_BASIC | rs116745220 | -0.6176 | 0.1324 | 3.09E-06 | -0.0023 | 0.0074 | 0.7536 |
| ALM | FGF_BASIC | rs13412535 | -0.1129 | 0.0224 | 4.76E-07 | -0.0016 | 0.0022 | 0.463 |
| ALM | FGF_BASIC | rs145577605 | 0.2043 | 0.0427 | 1.67E-06 | -0.0371 | 0.0121 | 0.002235 |
| ALM | FGF_BASIC | rs61990749 | 0.1124 | 0.0228 | 8.23E-07 | -0.0013 | 0.003 | 0.668599 |
| ALM | FGF_BASIC | rs75168112 | -0.1024 | 0.0214 | 1.64E-06 | -0.0012 | 0.0029 | 0.685499 |
| ALM | FGF_BASIC | rs78873483 | 0.1286 | 0.0282 | 4.98E-06 | -0.0013 | 0.0031 | 0.6693 |
| ALM | FGF_BASIC | rs9903590 | 0.1281 | 0.0267 | 1.62E-06 | -0.0056 | 0.0032 | 0.08324 |
| ALM | GROA | rs1113500 | 0.1162 | 0.0243 | 1.72E-06 | 0.0018 | 0.0019 | 0.3672 |
| ALM | GROA | rs114991247 | -0.2202 | 0.0463 | 1.97E-06 | 0.0144 | 0.0063 | 0.02292 |
| ALM | GROA | rs118158560 | 0.2761 | 0.0592 | 3.09E-06 | 0.0067 | 0.004 | 0.094161 |
| ALM | GROA | rs12075 | 0.3724 | 0.0236 | 3.46E-56 | -5.00E-04 | 0.0019 | 0.7891 |
| ALM | GROA | rs140734053 | 0.7333 | 0.1545 | 2.07E-06 | -0.0165 | 0.0073 | 0.02353 |
| ALM | GROA | rs185768063 | 0.4038 | 0.076 | 1.06E-07 | -0.0048 | 0.0102 | 0.635501 |
| ALM | GROA | rs188345231 | 0.6177 | 0.1322 | 2.97E-06 | 0.0078 | 0.0069 | 0.2623 |
| ALM | GROA | rs508977 | -0.3838 | 0.0279 | 4.57E-43 | -0.0026 | 0.0022 | 0.2392 |
| ALM | GROA | rs62024303 | -0.3013 | 0.066 | 4.91E-06 | -0.0064 | 0.0047 | 0.1722 |
| ALM | GROA | rs76390238 | 0.6223 | 0.1352 | 4.14E-06 | -0.0074 | 0.0066 | 0.2674 |
| ALM | GROA | rs78653452 | -0.7395 | 0.1559 | 2.09E-06 | 0.0166 | 0.0085 | 0.050441 |
| ALM | G_CSF | rs115256310 | -0.6788 | 0.1359 | 5.85E-07 | -0.0023 | 0.0074 | 0.7504 |
| ALM | G_CSF | rs117261691 | 0.1318 | 0.0288 | 4.67E-06 | -0.0063 | 0.007 | 0.369 |
| ALM | G_CSF | rs11903143 | 0.0889 | 0.0175 | 3.78E-07 | -3.00E-04 | 0.0021 | 0.8802 |
| ALM | G_CSF | rs145756094 | -0.7323 | 0.1479 | 7.40E-07 | 0.0066 | 0.0075 | 0.3778 |
| ALM | G_CSF | rs2671444 | -0.0776 | 0.0166 | 2.86E-06 | 9.00E-04 | 0.002 | 0.6486 |
| ALM | G_CSF | rs586313 | -0.0883 | 0.0187 | 2.36E-06 | -0.0023 | 0.0022 | 0.2875 |
| ALM | G_CSF | rs74148555 | -0.3771 | 0.0753 | 5.59E-07 | 0.0089 | 0.0055 | 0.1044 |
| ALM | G_CSF | rs77318030 | -0.2031 | 0.0427 | 2.02E-06 | 0.0018 | 0.0042 | 0.6709 |
| ALM | HGF | rs11060254 | -0.0765 | 0.0166 | 3.97E-06 | 7.00E-04 | 0.002 | 0.7347 |
| ALM | HGF | rs13412535 | -0.1043 | 0.0213 | 9.67E-07 | -0.0016 | 0.0022 | 0.463 |
| ALM | HGF | rs1617833 | -0.0749 | 0.016 | 2.93E-06 | -0.006 | 0.0019 | 0.001803 |
| ALM | HGF | rs180840563 | -0.2022 | 0.0416 | 1.15E-06 | -0.0042 | 0.0065 | 0.523801 |
| ALM | HGF | rs2003620 | 0.2277 | 0.0487 | 2.98E-06 | 0.003 | 0.0041 | 0.4719 |
| ALM | HGF | rs4245058 | -0.1552 | 0.0331 | 2.68E-06 | -0.0054 | 0.0032 | 0.095089 |
| ALM | HGF | rs57146176 | -0.0987 | 0.0208 | 2.18E-06 | -0.0104 | 0.0039 | 0.007489 |
| ALM | HGF | rs5745687 | -0.3008 | 0.0404 | 9.92E-14 | -0.007 | 0.0038 | 0.064699 |
| ALM | IFN_G | rs10481651 | -0.0793 | 0.0168 | 2.18E-06 | 8.00E-04 | 0.002 | 0.706801 |
| ALM | IFN_G | rs10761731 | -0.0813 | 0.0167 | 1.07E-06 | -0.0041 | 0.0019 | 0.03053 |
| ALM | IFN_G | rs113600793 | 0.1871 | 0.0371 | 4.43E-07 | -0.0053 | 0.005 | 0.2868 |
| ALM | IFN_G | rs115729819 | 0.2511 | 0.0514 | 1.05E-06 | -0.0217 | 0.0078 | 0.005732 |
| ALM | IFN_G | rs11843756 | 0.1812 | 0.0391 | 3.62E-06 | 0.0067 | 0.0059 | 0.2566 |
| ALM | IFN_G | rs12420286 | 0.2357 | 0.05 | 2.45E-06 | -0.0023 | 0.0047 | 0.630099 |
| ALM | IFN_G | rs2073438 | 0.092 | 0.0188 | 9.55E-07 | -1.00E-04 | 0.0021 | 0.9609 |
| ALM | IFN_G | rs2188420 | 0.1005 | 0.0201 | 5.90E-07 | 0.0012 | 0.0029 | 0.680899 |
| ALM | IFN_G | rs60059008 | 0.0852 | 0.0176 | 1.30E-06 | -0.0013 | 0.002 | 0.5028 |
| ALM | IFN_G | rs73479333 | -0.1123 | 0.024 | 2.82E-06 | 2.00E-04 | 0.0036 | 0.9624 |
| ALM | IFN_G | rs74148555 | -0.3771 | 0.077 | 9.86E-07 | 0.0089 | 0.0055 | 0.1044 |
| ALM | IFN_G | rs78296352 | 0.3419 | 0.065 | 1.42E-07 | 0.0038 | 0.0048 | 0.4308 |
| ALM | IL_10 | rs10457128 | -0.0854 | 0.0172 | 6.96E-07 | 0.0014 | 0.002 | 0.4815 |
| ALM | IL_10 | rs10493718 | -0.1081 | 0.0222 | 1.07E-06 | -2.00E-04 | 0.0022 | 0.9302 |
| ALM | IL_10 | rs10888839 | 0.1203 | 0.025 | 1.56E-06 | -0.0045 | 0.0035 | 0.201 |
| ALM | IL_10 | rs1530455 | 0.082 | 0.0174 | 2.53E-06 | -3.00E-04 | 0.0019 | 0.8685 |
| ALM | IL_10 | rs2086656 | -0.08 | 0.017 | 2.59E-06 | -0.0013 | 0.0021 | 0.534399 |
| ALM | IL_10 | rs282258 | 0.0993 | 0.0162 | 8.63E-10 | 5.00E-04 | 0.0019 | 0.8034 |
| ALM | IL_10 | rs3002131 | 0.1191 | 0.026 | 4.59E-06 | -0.0068 | 0.0029 | 0.02032 |
| ALM | IL_10 | rs3025021 | 0.0913 | 0.0194 | 2.61E-06 | 0.0024 | 0.0021 | 0.2439 |
| ALM | IL_10 | rs339203 | 0.0954 | 0.0203 | 2.75E-06 | 0.0029 | 0.0023 | 0.2115 |
| ALM | IL_10 | rs383684 | 0.092 | 0.0197 | 3.17E-06 | -0.0021 | 0.003 | 0.4781 |
| ALM | IL_10 | rs41282660 | -0.1169 | 0.0254 | 4.23E-06 | -0.0054 | 0.0029 | 0.06501 |
| ALM | IL_10 | rs6085948 | 0.0977 | 0.0202 | 1.28E-06 | 0.0037 | 0.0022 | 0.09925 |
| ALM | IL_10 | rs6799107 | -0.095 | 0.0206 | 3.99E-06 | 8.00E-04 | 0.0023 | 0.7296 |
| ALM | IL_10 | rs6921438 | -0.2876 | 0.0166 | 1.38E-67 | 2.00E-04 | 0.0019 | 0.927 |
| ALM | IL_10 | rs7088799 | -0.0815 | 0.0166 | 9.35E-07 | -0.0042 | 0.0019 | 0.03032 |
| ALM | IL_12_P70 | rs10761731 | -0.0965 | 0.0161 | 2.12E-09 | -0.0041 | 0.0019 | 0.03053 |
| ALM | IL_12_P70 | rs13209117 | 0.0981 | 0.0186 | 1.27E-07 | 0.0044 | 0.0021 | 0.03778 |
| ALM | IL_12_P70 | rs2123852 | 0.0942 | 0.0204 | 3.73E-06 | -0.007 | 0.0027 | 0.008421 |
| ALM | IL_12_P70 | rs273702 | -0.127 | 0.027 | 2.52E-06 | 0.0022 | 0.0029 | 0.454 |
| ALM | IL_12_P70 | rs282258 | 0.0726 | 0.0156 | 3.28E-06 | 5.00E-04 | 0.0019 | 0.8034 |
| ALM | IL_12_P70 | rs41282644 | 0.1401 | 0.0303 | 3.74E-06 | 0.0071 | 0.0046 | 0.1183 |
| ALM | IL_12_P70 | rs6532374 | -0.1033 | 0.0226 | 4.61E-06 | 0.0023 | 0.0025 | 0.3445 |
| ALM | IL_12_P70 | rs6921438 | -0.3784 | 0.016 | 5.78E-124 | 2.00E-04 | 0.0019 | 0.927 |
| ALM | IL_12_P70 | rs6993770 | 0.0918 | 0.0188 | 1.06E-06 | 0.0026 | 0.0021 | 0.2205 |
| ALM | IL_12_P70 | rs71361173 | 0.1105 | 0.0238 | 3.57E-06 | -0.0057 | 0.0027 | 0.03822 |
| ALM | IL_12_P70 | rs72831623 | 0.1929 | 0.0367 | 1.51E-07 | -0.0035 | 0.0045 | 0.4345 |
| ALM | IL_12_P70 | rs782107 | 0.0765 | 0.0156 | 9.13E-07 | 0.0017 | 0.0019 | 0.3725 |
| ALM | IL_12_P70 | rs9472183 | -0.1006 | 0.0157 | 1.38E-10 | -0.0052 | 0.002 | 0.008474 |
| ALM | IL_13 | rs10995615 | -0.1591 | 0.0341 | 3.12E-06 | -0.005 | 0.0024 | 0.03583 |
| ALM | IL_13 | rs117795020 | -0.3584 | 0.0716 | 5.48E-07 | 0.0039 | 0.007 | 0.576901 |
| ALM | IL_13 | rs12623722 | -0.1189 | 0.0257 | 3.61E-06 | -2.00E-04 | 0.0021 | 0.9077 |
| ALM | IL_13 | rs138854806 | -0.4204 | 0.0839 | 5.45E-07 | -0.0206 | 0.0143 | 0.1486 |
| ALM | IL_13 | rs139083458 | 0.9995 | 0.211 | 2.17E-06 | 0.0018 | 0.0072 | 0.8071 |
| ALM | IL_13 | rs147747784 | 0.369 | 0.0765 | 1.44E-06 | -0.0085 | 0.0052 | 0.1031 |
| ALM | IL_13 | rs6799107 | -0.1472 | 0.0299 | 8.66E-07 | 8.00E-04 | 0.0023 | 0.7296 |
| ALM | IL_13 | rs6921438 | -0.4139 | 0.0242 | 1.28E-65 | 2.00E-04 | 0.0019 | 0.927 |
| ALM | IL_13 | rs7073807 | 0.1618 | 0.0354 | 4.77E-06 | -0.0094 | 0.003 | 0.001751 |
| ALM | IL_13 | rs75383097 | -0.5369 | 0.116 | 3.70E-06 | -0.0202 | 0.008 | 0.01183 |
| ALM | IL_13 | rs76339001 | -0.4375 | 0.0886 | 7.92E-07 | 0.0052 | 0.006 | 0.3847 |
| ALM | IL_13 | rs77955971 | 0.4408 | 0.0868 | 3.76E-07 | -0.0039 | 0.006 | 0.5209 |
| ALM | IL_16 | rs116135478 | 0.8296 | 0.1637 | 4.05E-07 | -0.0131 | 0.0067 | 0.04899 |
| ALM | IL_16 | rs117916513 | -0.4713 | 0.0982 | 1.61E-06 | -0.0114 | 0.0085 | 0.1789 |
| ALM | IL_16 | rs1255143 | 0.1387 | 0.0241 | 8.53E-09 | -0.0022 | 0.0019 | 0.2586 |
| ALM | IL_16 | rs144691581 | 0.4929 | 0.0958 | 2.67E-07 | -0.0016 | 0.0081 | 0.8478 |
| ALM | IL_16 | rs4253283 | 0.1506 | 0.026 | 7.22E-09 | -0.0015 | 0.002 | 0.4643 |
| ALM | IL_16 | rs4778636 | -0.7286 | 0.063 | 6.21E-31 | 0.0083 | 0.0033 | 0.01089 |
| ALM | IL_16 | rs9706053 | 0.4412 | 0.0928 | 1.98E-06 | -7.00E-04 | 0.0064 | 0.9103 |
| ALM | IL_17 | rs11640734 | -0.115 | 0.024 | 1.61E-06 | 0.0049 | 0.0031 | 0.1082 |
| ALM | IL_17 | rs117556572 | -0.5256 | 0.1097 | 1.66E-06 | 0.006 | 0.0073 | 0.4082 |
| ALM | IL_17 | rs12735700 | -0.0943 | 0.0206 | 4.50E-06 | -0.0021 | 0.0022 | 0.332 |
| ALM | IL_17 | rs148562661 | 0.2161 | 0.0434 | 6.37E-07 | 0.0107 | 0.0076 | 0.1581 |
| ALM | IL_17 | rs149738638 | -0.1553 | 0.0337 | 4.11E-06 | -0.001 | 0.0037 | 0.781601 |
| ALM | IL_17 | rs1530455 | 0.1088 | 0.0173 | 3.29E-10 | -3.00E-04 | 0.0019 | 0.8685 |
| ALM | IL_17 | rs17106604 | 0.1119 | 0.0225 | 6.23E-07 | -6.00E-04 | 0.003 | 0.8318 |
| ALM | IL_17 | rs17282552 | -0.2026 | 0.0403 | 4.88E-07 | 0.0034 | 0.0053 | 0.5223 |
| ALM | IL_17 | rs184080173 | 0.236 | 0.0471 | 5.39E-07 | -0.0039 | 0.0038 | 0.3087 |
| ALM | IL_17 | rs78296352 | 0.2949 | 0.0645 | 4.81E-06 | 0.0038 | 0.0048 | 0.4308 |
| ALM | IL_17 | rs9568764 | 0.0825 | 0.018 | 4.68E-06 | 7.00E-04 | 0.0021 | 0.7366 |
| ALM | IL_18 | rs10414578 | -0.1817 | 0.0347 | 1.64E-07 | 0.0038 | 0.003 | 0.2119 |
| ALM | IL_18 | rs116383510 | -0.5412 | 0.1052 | 2.70E-07 | 0.0079 | 0.0083 | 0.342 |
| ALM | IL_18 | rs117266781 | 0.7051 | 0.1436 | 9.18E-07 | 0.0154 | 0.0086 | 0.073281 |
| ALM | IL_18 | rs12420140 | -0.2479 | 0.0261 | 1.95E-21 | 0.002 | 0.0021 | 0.3319 |
| ALM | IL_18 | rs143370787 | -0.3447 | 0.066 | 1.75E-07 | 0.006 | 0.0034 | 0.082279 |
| ALM | IL_18 | rs17229943 | -0.3076 | 0.0463 | 3.06E-11 | -0.0102 | 0.0043 | 0.01784 |
| ALM | IL_18 | rs1979967 | 0.14 | 0.0285 | 8.72E-07 | -0.0025 | 0.0023 | 0.2766 |
| ALM | IL_18 | rs385076 | -0.2472 | 0.0247 | 1.56E-23 | -0.0033 | 0.002 | 0.094111 |
| ALM | IL_18 | rs4482818 | 0.1233 | 0.0243 | 4.11E-07 | -3.00E-04 | 0.002 | 0.8978 |
| ALM | IL_18 | rs610473 | 0.1274 | 0.0242 | 1.43E-07 | -0.0054 | 0.002 | 0.006197 |
| ALM | IL_18 | rs7444013 | -0.5318 | 0.0955 | 2.59E-08 | 0.0203 | 0.0083 | 0.01441 |
| ALM | IL_18 | rs78716465 | 0.3173 | 0.0679 | 2.98E-06 | 0.0063 | 0.005 | 0.2043 |
| ALM | IL_1B | rs143319329 | 0.4357 | 0.093 | 2.84E-06 | 0.0019 | 0.006 | 0.7484 |
| ALM | IL_1B | rs61335305 | 0.4333 | 0.0928 | 3.02E-06 | 0.0058 | 0.0071 | 0.4181 |
| ALM | IL_1B | rs62015704 | 0.1786 | 0.0372 | 1.62E-06 | 0.0054 | 0.0029 | 0.06232 |
| ALM | IL_1RA | rs11627423 | 0.1178 | 0.0246 | 1.65E-06 | -0.0021 | 0.0019 | 0.2792 |
| ALM | IL_1RA | rs11869294 | -0.2286 | 0.047 | 1.13E-06 | 0.0032 | 0.0055 | 0.5662 |
| ALM | IL_1RA | rs147747784 | 0.3582 | 0.0754 | 2.04E-06 | -0.0085 | 0.0052 | 0.1031 |
| ALM | IL_1RA | rs187166731 | -0.2424 | 0.0504 | 1.55E-06 | 0.0157 | 0.014 | 0.2643 |
| ALM | IL_1RA | rs3876037 | 0.1234 | 0.027 | 4.73E-06 | 3.00E-04 | 0.002 | 0.8611 |
| ALM | IL_1RA | rs4441609 | 0.1056 | 0.0231 | 4.75E-06 | 0.0025 | 0.0019 | 0.2014 |
| ALM | IL_1RA | rs56134659 | -0.1109 | 0.0236 | 2.56E-06 | -0.005 | 0.002 | 0.01123 |
| ALM | IL_1RA | rs61335305 | 0.4315 | 0.0904 | 1.81E-06 | 0.0058 | 0.0071 | 0.4181 |
| ALM | IL_1RA | rs6699436 | -0.1858 | 0.0404 | 4.37E-06 | 7.00E-04 | 0.0027 | 0.8068 |
| ALM | IL_2 | rs13412535 | 0.174 | 0.0331 | 1.45E-07 | -0.0016 | 0.0022 | 0.463 |
| ALM | IL_2 | rs16836080 | 0.1158 | 0.0253 | 4.84E-06 | 0.0011 | 0.0021 | 0.5844 |
| ALM | IL_2 | rs170117 | -0.1637 | 0.0347 | 2.44E-06 | -9.00E-04 | 0.0028 | 0.7552 |
| ALM | IL_2 | rs2690020 | 0.1158 | 0.0245 | 2.27E-06 | 0.0025 | 0.0019 | 0.1919 |
| ALM | IL_2 | rs4634519 | -0.1249 | 0.0268 | 3.18E-06 | -0.002 | 0.0021 | 0.3399 |
| ALM | IL_2 | rs61335305 | 0.4439 | 0.0913 | 1.16E-06 | 0.0058 | 0.0071 | 0.4181 |
| ALM | IL_2 | rs62124990 | -0.7013 | 0.149 | 2.50E-06 | 0.018 | 0.0059 | 0.002459 |
| ALM | IL_2 | rs7615304 | -0.1139 | 0.024 | 2.16E-06 | -0.0032 | 0.002 | 0.099991 |
| ALM | IL_2RA | rs11241559 | -0.124 | 0.0264 | 2.75E-06 | 2.00E-04 | 0.0022 | 0.922 |
| ALM | IL_2RA | rs115360066 | 0.1776 | 0.0377 | 2.42E-06 | 0.0091 | 0.003 | 0.002779 |
| ALM | IL_2RA | rs117244812 | -0.7187 | 0.1493 | 1.47E-06 | 0.0094 | 0.0089 | 0.2916 |
| ALM | IL_2RA | rs12799226 | -0.1285 | 0.0277 | 3.56E-06 | 0.0034 | 0.0023 | 0.1417 |
| ALM | IL_2RA | rs185231391 | 0.8568 | 0.1803 | 2.00E-06 | -0.0076 | 0.0086 | 0.3751 |
| ALM | IL_2RA | rs28441585 | 0.1269 | 0.0271 | 2.93E-06 | 0.0068 | 0.0023 | 0.002924 |
| ALM | IL_2RA | rs4733117 | 0.1439 | 0.0291 | 7.91E-07 | 8.00E-04 | 0.0027 | 0.7782 |
| ALM | IL_4 | rs10512267 | -0.0824 | 0.016 | 2.73E-07 | -3.00E-04 | 0.002 | 0.8842 |
| ALM | IL_4 | rs116705532 | -0.4675 | 0.0978 | 1.73E-06 | 0.0072 | 0.0074 | 0.3307 |
| ALM | IL_4 | rs117146485 | -0.2856 | 0.0625 | 4.95E-06 | -0.0284 | 0.0095 | 0.002834 |
| ALM | IL_4 | rs12238729 | 0.5271 | 0.1096 | 1.51E-06 | 5.00E-04 | 0.0083 | 0.9544 |
| ALM | IL_4 | rs13106889 | -0.1186 | 0.0224 | 1.22E-07 | -0.0038 | 0.0026 | 0.1443 |
| ALM | IL_4 | rs17713451 | 0.1255 | 0.0252 | 6.41E-07 | -0.0019 | 0.0027 | 0.49 |
| ALM | IL_4 | rs2073438 | 0.0847 | 0.0183 | 3.73E-06 | -1.00E-04 | 0.0021 | 0.9609 |
| ALM | IL_4 | rs58202480 | -0.0767 | 0.0166 | 3.59E-06 | 0.0061 | 0.0024 | 0.00967 |
| ALM | IL_4 | rs73023729 | -0.1796 | 0.0365 | 8.56E-07 | -0.0054 | 0.0075 | 0.47 |
| ALM | IL_4 | rs7613691 | 0.1787 | 0.0382 | 2.96E-06 | -0.0088 | 0.0039 | 0.02364 |
| ALM | IL_4 | rs79597994 | -0.5855 | 0.1271 | 4.06E-06 | -0.0042 | 0.006 | 0.4809 |
| ALM | IL_4 | rs9508291 | -0.168 | 0.0358 | 2.67E-06 | 0.0024 | 0.0038 | 0.5273 |
| ALM | IL_4 | rs9941733 | 0.1156 | 0.0229 | 4.33E-07 | -0.0021 | 0.0025 | 0.4088 |
| ALM | IL_5 | rs11680908 | 0.2593 | 0.0552 | 2.62E-06 | -0.0033 | 0.0038 | 0.3847 |
| ALM | IL_5 | rs148634917 | -0.517 | 0.1087 | 1.97E-06 | -0.0096 | 0.007 | 0.1716 |
| ALM | IL_5 | rs28793375 | 0.1697 | 0.0362 | 2.75E-06 | 0.0023 | 0.0028 | 0.409 |
| ALM | IL_5 | rs6737109 | 0.1135 | 0.0246 | 3.81E-06 | 0.0016 | 0.0019 | 0.4048 |
| ALM | IL_5 | rs72831687 | -0.5337 | 0.1104 | 1.32E-06 | 0.002 | 0.0105 | 0.8478 |
| ALM | IL_5 | rs73040130 | 0.2745 | 0.0525 | 1.71E-07 | 0.0083 | 0.004 | 0.0385 |
| ALM | IL_5 | rs74811276 | 0.217 | 0.0471 | 4.08E-06 | -0.0045 | 0.0035 | 0.199 |
| ALM | IL_5 | rs9472168 | 0.1568 | 0.0253 | 5.42E-10 | 1.00E-04 | 0.0019 | 0.9774 |
| ALM | IL_6 | rs10752777 | 0.1083 | 0.0235 | 4.17E-06 | -0.0017 | 0.0035 | 0.6239 |
| ALM | IL_6 | rs113098456 | -0.1553 | 0.0339 | 4.64E-06 | 0.0023 | 0.0038 | 0.5489 |
| ALM | IL_6 | rs1333040 | 0.0747 | 0.0157 | 1.99E-06 | 0.0029 | 0.0019 | 0.1355 |
| ALM | IL_6 | rs13412535 | -0.1186 | 0.0214 | 3.14E-08 | -0.0016 | 0.0022 | 0.463 |
| ALM | IL_6 | rs2404476 | 0.0734 | 0.0156 | 2.68E-06 | -0.0022 | 0.0019 | 0.2413 |
| ALM | IL_6 | rs4684700 | -0.0747 | 0.0162 | 3.91E-06 | 0.0037 | 0.0019 | 0.05365 |
| ALM | IL_6 | rs72831623 | 0.197 | 0.0369 | 9.29E-08 | -0.0035 | 0.0045 | 0.4345 |
| ALM | IL_6 | rs73273528 | 0.268 | 0.0553 | 1.25E-06 | -2.00E-04 | 0.0052 | 0.9659 |
| ALM | IL_6 | rs75101555 | -0.3625 | 0.0781 | 3.44E-06 | 0.0068 | 0.006 | 0.2564 |
| ALM | IL_6 | rs76856708 | 0.336 | 0.0697 | 1.43E-06 | -0.0071 | 0.005 | 0.1598 |
| ALM | IL_7 | rs10196226 | 0.1538 | 0.0327 | 2.50E-06 | -0.0034 | 0.0029 | 0.2452 |
| ALM | IL_7 | rs115215018 | 0.5985 | 0.1308 | 4.76E-06 | -0.0144 | 0.0072 | 0.04447 |
| ALM | IL_7 | rs117509142 | -0.3213 | 0.0684 | 2.60E-06 | 0.0027 | 0.0051 | 0.5933 |
| ALM | IL_7 | rs141425475 | -0.4801 | 0.1018 | 2.39E-06 | -0.0103 | 0.006 | 0.08433 |
| ALM | IL_7 | rs147747784 | 0.411 | 0.0777 | 1.22E-07 | -0.0085 | 0.0052 | 0.1031 |
| ALM | IL_7 | rs17091524 | 0.5092 | 0.1015 | 5.24E-07 | 0.01 | 0.0056 | 0.072171 |
| ALM | IL_7 | rs1958987 | 0.1261 | 0.0263 | 1.60E-06 | 0.0021 | 0.002 | 0.3067 |
| ALM | IL_7 | rs218247 | -0.1343 | 0.0285 | 2.42E-06 | 0.0027 | 0.0023 | 0.2331 |
| ALM | IL_7 | rs28793375 | 0.1644 | 0.036 | 4.87E-06 | 0.0023 | 0.0028 | 0.409 |
| ALM | IL_7 | rs62006410 | -0.1492 | 0.0302 | 7.59E-07 | 0.0027 | 0.0024 | 0.2588 |
| ALM | IL_7 | rs6921438 | -0.3204 | 0.0246 | 8.71E-39 | 2.00E-04 | 0.0019 | 0.927 |
| ALM | IL_7 | rs77981494 | -0.5201 | 0.1055 | 8.23E-07 | 0.003 | 0.0067 | 0.6507 |
| ALM | IL_7 | rs78346957 | 0.4632 | 0.1008 | 4.30E-06 | -0.0136 | 0.0069 | 0.05053 |
| ALM | IL_8 | rs116726256 | -0.2247 | 0.0489 | 4.26E-06 | 0.0169 | 0.0075 | 0.02333 |
| ALM | IL_8 | rs12075 | 0.1148 | 0.0235 | 9.97E-07 | -5.00E-04 | 0.0019 | 0.7891 |
| ALM | IL_8 | rs12438669 | -0.1182 | 0.0252 | 2.60E-06 | 0.0037 | 0.002 | 0.063729 |
| ALM | IL_8 | rs141926526 | -0.6221 | 0.1308 | 1.96E-06 | 0.0014 | 0.0049 | 0.771699 |
| ALM | IL_8 | rs183628733 | 0.6547 | 0.1417 | 3.82E-06 | 5.00E-04 | 0.0067 | 0.9365 |
| ALM | IL_8 | rs2673604 | -0.118 | 0.0254 | 3.29E-06 | 0.0022 | 0.0021 | 0.294 |
| ALM | IL_8 | rs3786107 | 0.2463 | 0.0517 | 1.94E-06 | 0.004 | 0.0042 | 0.3361 |
| ALM | IL_8 | rs75840288 | 0.5125 | 0.1121 | 4.85E-06 | 0.0041 | 0.005 | 0.409 |
| ALM | IL_9 | rs117807175 | -0.5225 | 0.1106 | 2.33E-06 | 0.0094 | 0.0058 | 0.1051 |
| ALM | IL_9 | rs3736858 | -0.1351 | 0.0291 | 3.37E-06 | 0.0069 | 0.0027 | 0.009429 |
| ALM | IL_9 | rs41294750 | 0.3442 | 0.0736 | 2.92E-06 | -0.0016 | 0.0056 | 0.7784 |
| ALM | IL_9 | rs4880409 | -0.3552 | 0.0716 | 6.95E-07 | 0.001 | 0.0059 | 0.8591 |
| ALM | IL_9 | rs73443903 | 0.2162 | 0.046 | 2.57E-06 | -0.0032 | 0.0033 | 0.3281 |
| ALM | IL_9 | rs76963786 | -0.2856 | 0.0556 | 2.78E-07 | -0.0016 | 0.0033 | 0.6294 |
| ALM | IP_10 | rs113831257 | 0.3639 | 0.0641 | 1.39E-08 | -0.0024 | 0.0049 | 0.6203 |
| ALM | IP_10 | rs143799975 | -0.7551 | 0.1638 | 4.01E-06 | -0.0068 | 0.0087 | 0.4371 |
| ALM | IP_10 | rs34383175 | -0.3196 | 0.0653 | 9.90E-07 | -0.0091 | 0.0051 | 0.07391 |
| ALM | IP_10 | rs397816 | 0.1211 | 0.0248 | 1.03E-06 | 0.0015 | 0.0019 | 0.4385 |
| ALM | IP_10 | rs4862111 | 0.1448 | 0.0317 | 4.84E-06 | 0.0029 | 0.0025 | 0.2486 |
| ALM | IP_10 | rs6707974 | 0.1574 | 0.0337 | 3.03E-06 | -0.0021 | 0.0026 | 0.4181 |
| ALM | IP_10 | rs75970138 | -0.4845 | 0.1037 | 2.99E-06 | 0.0092 | 0.0106 | 0.3837 |
| ALM | IP_10 | rs7645625 | -0.1116 | 0.0236 | 2.19E-06 | -0.0027 | 0.0019 | 0.1605 |
| ALM | IP_10 | rs79848609 | 0.2514 | 0.0535 | 2.64E-06 | -5.00E-04 | 0.0046 | 0.9159 |
| ALM | IP_10 | rs8112909 | -0.139 | 0.0297 | 2.96E-06 | -8.00E-04 | 0.0024 | 0.7461 |
| ALM | IP_10 | rs9450351 | -0.2651 | 0.0488 | 5.48E-08 | -0.0088 | 0.0038 | 0.02216 |
| ALM | MCP_1_MCAF | rs10744620 | 0.0783 | 0.0161 | 1.12E-06 | -0.0027 | 0.002 | 0.1708 |
| ALM | MCP_1_MCAF | rs111995966 | 0.1428 | 0.0309 | 3.79E-06 | 1.00E-04 | 0.007 | 0.9903 |
| ALM | MCP_1_MCAF | rs12073356 | -0.1436 | 0.031 | 3.49E-06 | -0.0017 | 0.0038 | 0.6528 |
| ALM | MCP_1_MCAF | rs12075 | 0.2186 | 0.0154 | 1.36E-45 | -5.00E-04 | 0.0019 | 0.7891 |
| ALM | MCP_1_MCAF | rs143815843 | -0.2049 | 0.0447 | 4.61E-06 | 0.0054 | 0.0102 | 0.5991 |
| ALM | MCP_1_MCAF | rs146522229 | -0.5942 | 0.1161 | 3.09E-07 | 0.0018 | 0.0086 | 0.8379 |
| ALM | MCP_1_MCAF | rs2036297 | 0.1182 | 0.016 | 1.30E-13 | -0.0039 | 0.002 | 0.05015 |
| ALM | MCP_1_MCAF | rs2288370 | -0.1036 | 0.0162 | 1.56E-10 | -4.00E-04 | 0.002 | 0.8533 |
| ALM | MCP_1_MCAF | rs56212190 | 0.1799 | 0.0372 | 1.32E-06 | 0.0015 | 0.0046 | 0.7428 |
| ALM | MCP_1_MCAF | rs7033586 | -0.22 | 0.0467 | 2.43E-06 | -0.0135 | 0.0205 | 0.5091 |
| ALM | MCP_1_MCAF | rs7197349 | 0.0971 | 0.0206 | 2.40E-06 | -4.00E-04 | 0.0029 | 0.8952 |
| ALM | MCP_1_MCAF | rs7517040 | -0.097 | 0.019 | 3.41E-07 | 0.0039 | 0.0021 | 0.06785 |
| ALM | MCP_1_MCAF | rs9317045 | 0.1157 | 0.0235 | 8.43E-07 | -0.0019 | 0.0026 | 0.4591 |
| ALM | MCP_3 | rs10892381 | 0.2432 | 0.0473 | 2.69E-07 | -0.0029 | 0.0021 | 0.1665 |
| ALM | MCP_3 | rs117286643 | 0.6934 | 0.1474 | 2.54E-06 | -4.00E-04 | 0.0075 | 0.9538 |
| ALM | MCP_3 | rs2838065 | -0.221 | 0.0479 | 3.92E-06 | 0.0061 | 0.0024 | 0.01264 |
| ALM | MCP_3 | rs28394764 | 0.597 | 0.1282 | 3.19E-06 | 0.0018 | 0.0043 | 0.6698 |
| ALM | MCP_3 | rs3129806 | -0.1975 | 0.0433 | 4.98E-06 | 9.00E-04 | 0.002 | 0.646399 |
| ALM | MCP_3 | rs62492260 | -0.2802 | 0.0578 | 1.23E-06 | 0.0013 | 0.0028 | 0.6402 |
| ALM | MIF | rs113218956 | -0.8789 | 0.1876 | 2.82E-06 | -0.0166 | 0.0148 | 0.2617 |
| ALM | MIF | rs11551183 | 0.3666 | 0.0795 | 4.00E-06 | -0.0044 | 0.005 | 0.3841 |
| ALM | MIF | rs12594190 | 0.1321 | 0.0266 | 6.85E-07 | -9.00E-04 | 0.0023 | 0.7019 |
| ALM | MIF | rs13142904 | -0.2232 | 0.0425 | 1.47E-07 | -1.00E-04 | 0.0038 | 0.9894 |
| ALM | MIF | rs2294689 | -0.1338 | 0.0287 | 3.04E-06 | -0.0067 | 0.0043 | 0.1166 |
| ALM | MIF | rs2330634 | 0.1549 | 0.0249 | 4.57E-10 | -0.0048 | 0.0019 | 0.01468 |
| ALM | MIF | rs35890933 | 0.1676 | 0.0365 | 4.46E-06 | 0.0027 | 0.0024 | 0.2509 |
| ALM | MIF | rs3814097 | -0.1163 | 0.0251 | 3.55E-06 | -9.00E-04 | 0.0019 | 0.6567 |
| ALM | MIF | rs78098071 | -0.4583 | 0.0915 | 5.51E-07 | 0.0036 | 0.0088 | 0.6799 |
| ALM | MIG | rs111607343 | -0.5235 | 0.1119 | 2.93E-06 | 1.00E-04 | 0.0055 | 0.992 |
| ALM | MIG | rs11177248 | 0.3157 | 0.0667 | 2.22E-06 | 0.0011 | 0.0038 | 0.783401 |
| ALM | MIG | rs112861654 | -0.2682 | 0.0527 | 3.64E-07 | 0.0013 | 0.0034 | 0.6941 |
| ALM | MIG | rs117831247 | -0.8819 | 0.173 | 3.45E-07 | -0.005 | 0.0088 | 0.5714 |
| ALM | MIG | rs13143163 | 0.2735 | 0.0582 | 2.62E-06 | -0.0061 | 0.004 | 0.1316 |
| ALM | MIG | rs139010077 | 0.4337 | 0.0943 | 4.19E-06 | 0.0158 | 0.0082 | 0.05535 |
| ALM | MIG | rs1796086 | -0.2172 | 0.04 | 5.62E-08 | 0.0061 | 0.0034 | 0.071381 |
| ALM | MIG | rs191555775 | 0.2279 | 0.0412 | 3.28E-08 | 0.0081 | 0.0031 | 0.008175 |
| ALM | MIG | rs55876513 | 0.1638 | 0.0254 | 1.05E-10 | 8.00E-04 | 0.0022 | 0.7059 |
| ALM | MIG | rs62562991 | 0.6239 | 0.1259 | 7.24E-07 | 0.014 | 0.0069 | 0.0436 |
| ALM | MIG | rs6679677 | 0.1628 | 0.0327 | 6.51E-07 | 0.0057 | 0.0031 | 0.067671 |
| ALM | MIG | rs77086208 | 0.327 | 0.0694 | 2.50E-06 | -0.002 | 0.0073 | 0.7861 |
| ALM | MIG | rs816960 | -0.1179 | 0.0242 | 1.15E-06 | 9.00E-04 | 0.0023 | 0.6946 |
| ALM | MIP_1A | rs116615337 | 0.1286 | 0.0278 | 3.66E-06 | -1.00E-04 | 0.0022 | 0.9495 |
| ALM | MIP_1A | rs12690897 | 0.1215 | 0.026 | 3.07E-06 | -2.00E-04 | 0.0022 | 0.9151 |
| ALM | MIP_1A | rs184154340 | 0.3251 | 0.0689 | 2.40E-06 | -0.0113 | 0.0046 | 0.01546 |
| ALM | MIP_1A | rs57786342 | 0.139 | 0.0283 | 8.91E-07 | 0.0023 | 0.0024 | 0.3333 |
| ALM | MIP_1A | rs60198979 | -0.2154 | 0.0455 | 2.22E-06 | 0.0069 | 0.0035 | 0.04934 |
| ALM | MIP_1A | rs6900267 | -0.2472 | 0.0515 | 1.60E-06 | -0.0052 | 0.0044 | 0.2406 |
| ALM | MIP_1B | rs113010081 | -0.5799 | 0.0236 | 1.57E-133 | 0.0069 | 0.003 | 0.02005 |
| ALM | MIP_1B | rs113877493 | -0.607 | 0.0217 | 3.67E-172 | -0.0025 | 0.0035 | 0.4669 |
| ALM | MIP_1B | rs117453826 | -0.5907 | 0.0591 | 1.53E-23 | -0.003 | 0.0075 | 0.684699 |
| ALM | MIP_1B | rs117657747 | 0.2089 | 0.0453 | 4.01E-06 | 0.0011 | 0.0039 | 0.7833 |
| ALM | MIP_1B | rs141102180 | 0.3298 | 0.0392 | 3.75E-17 | 0.0067 | 0.0073 | 0.3644 |
| ALM | MIP_1B | rs1437220 | 0.1437 | 0.0315 | 4.92E-06 | -5.00E-04 | 0.0043 | 0.9017 |
| ALM | MIP_1B | rs17138331 | -0.1434 | 0.0295 | 1.13E-06 | -0.0019 | 0.0029 | 0.5247 |
| ALM | MIP_1B | rs2411161 | 0.1719 | 0.0365 | 2.55E-06 | -5.00E-04 | 0.0042 | 0.9009 |
| ALM | MIP_1B | rs281748 | -0.0794 | 0.0171 | 3.28E-06 | -0.0014 | 0.002 | 0.482 |
| ALM | MIP_1B | rs3760440 | 0.1242 | 0.0162 | 1.73E-14 | -2.00E-04 | 0.002 | 0.9022 |
| ALM | MIP_1B | rs5743614 | 0.1115 | 0.0232 | 1.57E-06 | 3.00E-04 | 0.0023 | 0.8998 |
| ALM | MIP_1B | rs6908843 | 0.0997 | 0.0209 | 1.78E-06 | 0.0018 | 0.0024 | 0.4503 |
| ALM | MIP_1B | rs72791296 | 0.2364 | 0.0466 | 3.97E-07 | -0.0051 | 0.0046 | 0.261 |
| ALM | MIP_1B | rs72799710 | -0.1037 | 0.0217 | 1.79E-06 | 0.0014 | 0.0024 | 0.574101 |
| ALM | MIP_1B | rs76356863 | -0.3456 | 0.0667 | 2.22E-07 | -0.0104 | 0.0056 | 0.06085 |
| ALM | MIP_1B | rs76582507 | 0.3259 | 0.0676 | 1.42E-06 | 0.0121 | 0.0074 | 0.1045 |
| ALM | MIP_1B | rs76776296 | 0.313 | 0.0598 | 1.63E-07 | -0.0016 | 0.0049 | 0.736901 |
| ALM | MIP_1B | rs79068918 | 0.2674 | 0.0271 | 5.54E-23 | -0.0024 | 0.0032 | 0.454099 |
| ALM | M_CSF | rs116274860 | 0.8262 | 0.1739 | 2.03E-06 | 0.0134 | 0.0075 | 0.07369 |
| ALM | M_CSF | rs117867915 | 0.5224 | 0.1096 | 1.87E-06 | 0.006 | 0.0096 | 0.5336 |
| ALM | M_CSF | rs11963606 | -0.5353 | 0.117 | 4.73E-06 | 0.0072 | 0.0076 | 0.3408 |
| ALM | M_CSF | rs12962919 | 0.3025 | 0.0659 | 4.39E-06 | 0.0054 | 0.0031 | 0.08572 |
| ALM | M_CSF | rs34089869 | 0.2194 | 0.0462 | 2.08E-06 | 0.0039 | 0.003 | 0.1882 |
| ALM | M_CSF | rs4269021 | -0.2459 | 0.0504 | 1.05E-06 | -0.0055 | 0.0028 | 0.04788 |
| ALM | M_CSF | rs56367447 | -0.4878 | 0.0876 | 2.57E-08 | -0.0091 | 0.0049 | 0.06465 |
| ALM | M_CSF | rs62294910 | 0.3472 | 0.0687 | 4.38E-07 | -0.0011 | 0.0041 | 0.796 |
| ALM | M_CSF | rs72723242 | -0.4969 | 0.1083 | 4.43E-06 | -9.00E-04 | 0.0041 | 0.8189 |
| ALM | M_CSF | rs78296352 | 0.522 | 0.111 | 2.58E-06 | 0.0038 | 0.0048 | 0.4308 |
| ALM | M_CSF | rs9387100 | -0.135 | 0.029 | 3.34E-06 | -0.001 | 0.002 | 0.606599 |
| ALM | PDGF_BB | rs11247305 | -0.1687 | 0.0364 | 3.47E-06 | 0.0062 | 0.0052 | 0.2324 |
| ALM | PDGF_BB | rs116445074 | 0.2869 | 0.0587 | 1.02E-06 | 0.0077 | 0.0078 | 0.3248 |
| ALM | PDGF_BB | rs11766649 | 0.0902 | 0.0196 | 3.96E-06 | 0.0049 | 0.0022 | 0.02233 |
| ALM | PDGF_BB | rs12289510 | -0.0772 | 0.0158 | 1.00E-06 | -0.0016 | 0.0019 | 0.3904 |
| ALM | PDGF_BB | rs13037046 | -0.0948 | 0.0206 | 3.96E-06 | -0.0042 | 0.0023 | 0.064961 |
| ALM | PDGF_BB | rs13412535 | 0.3317 | 0.0214 | 2.89E-54 | -0.0016 | 0.0022 | 0.463 |
| ALM | PDGF_BB | rs2324229 | 0.0884 | 0.0161 | 4.02E-08 | 0.0015 | 0.002 | 0.4431 |
| ALM | PDGF_BB | rs35859699 | -0.3854 | 0.0838 | 4.22E-06 | -0.0094 | 0.0063 | 0.1392 |
| ALM | PDGF_BB | rs4965869 | 0.1843 | 0.0181 | 2.22E-24 | -2.00E-04 | 0.0021 | 0.9381 |
| ALM | PDGF_BB | rs72777070 | -0.1048 | 0.02 | 1.56E-07 | 0.007 | 0.0024 | 0.003784 |
| ALM | PDGF_BB | rs73162807 | -0.2313 | 0.0499 | 3.55E-06 | 0.0155 | 0.0065 | 0.01766 |
| ALM | PDGF_BB | rs9936075 | -0.0767 | 0.0163 | 2.68E-06 | -0.0037 | 0.002 | 0.06501 |
| ALM | PDGF_BB | rs9941733 | 0.1165 | 0.0227 | 3.02E-07 | -0.0021 | 0.0025 | 0.4088 |
| ALM | RANTES | rs112072646 | 0.4209 | 0.0859 | 9.62E-07 | 0.0027 | 0.0057 | 0.6308 |
| ALM | RANTES | rs147509526 | -0.3558 | 0.0715 | 6.57E-07 | -0.0051 | 0.008 | 0.524901 |
| ALM | RANTES | rs2251660 | 0.1831 | 0.0356 | 2.69E-07 | -0.007 | 0.0027 | 0.008625 |
| ALM | RANTES | rs62438851 | -0.1904 | 0.0413 | 4.01E-06 | -0.0062 | 0.0028 | 0.02348 |
| ALM | RANTES | rs7000423 | -0.1314 | 0.0252 | 1.85E-07 | -0.0022 | 0.002 | 0.2871 |
| ALM | RANTES | rs7170339 | -0.4283 | 0.0904 | 2.19E-06 | 8.00E-04 | 0.0069 | 0.9104 |
| ALM | RANTES | rs72793342 | -0.1505 | 0.0307 | 9.08E-07 | -5.00E-04 | 0.0024 | 0.8199 |
| ALM | RANTES | rs74472919 | 0.3547 | 0.06 | 3.35E-09 | 0.0103 | 0.0055 | 0.06052 |
| ALM | RANTES | rs9675798 | -0.2583 | 0.0552 | 2.89E-06 | 0.009 | 0.005 | 0.075341 |
| ALM | SCF | rs113127926 | 0.1974 | 0.0418 | 2.34E-06 | -0.0058 | 0.0038 | 0.1246 |
| ALM | SCF | rs13412535 | -0.1065 | 0.0213 | 5.59E-07 | -0.0016 | 0.0022 | 0.463 |
| ALM | SCF | rs1557570 | 0.1172 | 0.0169 | 4.13E-12 | -0.0019 | 0.002 | 0.3402 |
| ALM | SCF | rs1568119 | -0.5946 | 0.1129 | 1.37E-07 | -0.0062 | 0.0099 | 0.5329 |
| ALM | SCF | rs4841899 | -0.1002 | 0.0178 | 1.67E-08 | 0.002 | 0.002 | 0.3339 |
| ALM | SCF | rs635634 | -0.1035 | 0.0191 | 5.71E-08 | -0.0019 | 0.0024 | 0.446 |
| ALM | SCF | rs7039247 | 0.079 | 0.0168 | 2.46E-06 | 0.0026 | 0.002 | 0.1894 |
| ALM | SCF | rs72678285 | 0.1062 | 0.0231 | 4.43E-06 | 0.0063 | 0.0026 | 0.01639 |
| ALM | SCF | rs78369473 | -0.2256 | 0.0484 | 3.14E-06 | 0.0062 | 0.0056 | 0.2671 |
| ALM | SCF | rs78666213 | -0.2845 | 0.0574 | 7.15E-07 | 0.0017 | 0.0054 | 0.747699 |
| ALM | SCF | rs80271436 | -0.2393 | 0.0484 | 7.49E-07 | 0.0037 | 0.0044 | 0.3952 |
| ALM | SCGF_B | rs1149926 | -0.3458 | 0.0749 | 3.92E-06 | -0.0035 | 0.0059 | 0.547299 |
| ALM | SCGF_B | rs116924815 | 0.6046 | 0.0737 | 2.25E-16 | -0.0066 | 0.0057 | 0.2477 |
| ALM | SCGF_B | rs117716477 | 0.8242 | 0.084 | 1.03E-22 | 0.0073 | 0.008 | 0.3669 |
| ALM | SCGF_B | rs12480722 | 0.1654 | 0.0353 | 2.81E-06 | 0.0053 | 0.003 | 0.07591 |
| ALM | SCGF_B | rs13287050 | -0.121 | 0.0263 | 4.12E-06 | -0.0022 | 0.002 | 0.2884 |
| ALM | SCGF_B | rs139413256 | -0.5174 | 0.1076 | 1.53E-06 | 0.0014 | 0.005 | 0.780001 |
| ALM | SCGF_B | rs143829871 | -0.1866 | 0.0399 | 2.85E-06 | 0.0045 | 0.0039 | 0.2462 |
| ALM | SCGF_B | rs149009264 | 0.4551 | 0.0985 | 3.79E-06 | 0.0081 | 0.0084 | 0.3329 |
| ALM | SCGF_B | rs150733161 | -0.5255 | 0.112 | 2.69E-06 | -0.0065 | 0.0081 | 0.4211 |
| ALM | SCGF_B | rs151194174 | 0.4536 | 0.0941 | 1.45E-06 | 1.00E-04 | 0.0067 | 0.9927 |
| ALM | SCGF_B | rs264157 | 0.1079 | 0.0233 | 3.69E-06 | -0.0012 | 0.0019 | 0.5272 |
| ALM | SCGF_B | rs34911860 | -0.3674 | 0.0787 | 3.00E-06 | 0.0044 | 0.0097 | 0.6479 |
| ALM | SCGF_B | rs4656185 | 0.2103 | 0.0254 | 1.29E-16 | -0.0017 | 0.002 | 0.3848 |
| ALM | SCGF_B | rs77954165 | 0.2631 | 0.0562 | 2.87E-06 | -0.0044 | 0.0033 | 0.1785 |
| ALM | SCGF_B | rs7815967 | 0.1325 | 0.0288 | 4.37E-06 | -0.003 | 0.0029 | 0.3077 |
| ALM | SCGF_B | rs78217154 | 0.3942 | 0.0861 | 4.72E-06 | -0.0042 | 0.0066 | 0.5246 |
| ALM | SDF_1A | rs10013755 | 0.5188 | 0.0995 | 1.85E-07 | -0.011 | 0.0069 | 0.1131 |
| ALM | SDF_1A | rs10474392 | 0.0934 | 0.0177 | 1.38E-07 | 0.001 | 0.0021 | 0.6367 |
| ALM | SDF_1A | rs12141941 | -0.0881 | 0.0186 | 2.26E-06 | -9.00E-04 | 0.0021 | 0.6747 |
| ALM | SDF_1A | rs149893336 | -0.494 | 0.1082 | 4.93E-06 | 0.0056 | 0.0071 | 0.4327 |
| ALM | SDF_1A | rs1600396 | -0.0933 | 0.0204 | 4.94E-06 | 0.0019 | 0.0022 | 0.379 |
| ALM | SDF_1A | rs3988298 | -0.1263 | 0.0266 | 2.12E-06 | -0.0056 | 0.0029 | 0.0541 |
| ALM | SDF_1A | rs62194947 | -0.0852 | 0.0185 | 4.27E-06 | -0.0067 | 0.0021 | 0.001781 |
| ALM | SDF_1A | rs78037609 | -0.6261 | 0.1334 | 2.67E-06 | -0.016 | 0.0112 | 0.1531 |
| ALM | TNF_A | rs10834997 | -0.123 | 0.0256 | 1.53E-06 | 0.0017 | 0.002 | 0.415 |
| ALM | TNF_A | rs115669577 | 0.981 | 0.1994 | 8.63E-07 | 0.0215 | 0.0099 | 0.02978 |
| ALM | TNF_A | rs79105320 | 0.5573 | 0.1177 | 2.21E-06 | -0.0013 | 0.0074 | 0.8545 |
| ALM | TNF_B | rs10925040 | 0.1738 | 0.0372 | 2.93E-06 | -0.0027 | 0.0019 | 0.1674 |
| ALM | TNF_B | rs75240021 | 0.3713 | 0.0772 | 1.49E-06 | 0.0031 | 0.0036 | 0.3954 |
| ALM | TNF_B | rs753274 | -0.1725 | 0.037 | 3.14E-06 | -0.001 | 0.0019 | 0.6031 |
| ALM | TNF_B | rs7629875 | 0.3841 | 0.0774 | 6.90E-07 | -0.0014 | 0.0043 | 0.747799 |
| ALM | TNF_B | rs78296352 | 1.2028 | 0.1366 | 1.28E-18 | 0.0038 | 0.0048 | 0.4308 |
| ALM | TRAIL | rs13278062 | 0.08 | 0.0157 | 3.33E-07 | -0.0017 | 0.0019 | 0.3673 |
| ALM | TRAIL | rs138987090 | -0.7264 | 0.0749 | 2.97E-22 | -0.0092 | 0.0117 | 0.4338 |
| ALM | TRAIL | rs148051545 | -0.4211 | 0.0843 | 5.84E-07 | -0.001 | 0.0061 | 0.8682 |
| ALM | TRAIL | rs17434886 | -0.0918 | 0.0199 | 4.20E-06 | 4.00E-04 | 0.0026 | 0.8806 |
| ALM | TRAIL | rs193112415 | -1.0456 | 0.062 | 1.01E-63 | -0.0026 | 0.007 | 0.706601 |
| ALM | TRAIL | rs28431810 | -0.1216 | 0.0252 | 1.41E-06 | 0.009 | 0.0102 | 0.3761 |
| ALM | TRAIL | rs28521641 | -0.7004 | 0.0445 | 7.79E-56 | 0.0069 | 0.0053 | 0.1958 |
| ALM | TRAIL | rs550057 | -0.0783 | 0.0169 | 3.71E-06 | -8.00E-04 | 0.0022 | 0.721099 |
| ALM | TRAIL | rs57396456 | -0.5641 | 0.0516 | 7.71E-28 | 0.0085 | 0.0055 | 0.1195 |
| ALM | TRAIL | rs62093514 | 1.0459 | 0.0549 | 5.80E-81 | 0.0072 | 0.0059 | 0.227 |
| ALM | TRAIL | rs72899452 | 0.1223 | 0.0264 | 3.75E-06 | -0.0116 | 0.0038 | 0.002553 |
| ALM | TRAIL | rs73039026 | -0.3098 | 0.0634 | 1.02E-06 | -0.0119 | 0.0087 | 0.174 |
| ALM | TRAIL | rs747324 | -0.0826 | 0.0178 | 3.34E-06 | -0.0021 | 0.002 | 0.3055 |
| ALM | TRAIL | rs74778900 | 0.5791 | 0.0531 | 9.90E-28 | 0.0041 | 0.0079 | 0.6052 |
| ALM | TRAIL | rs75928541 | 0.2784 | 0.0591 | 2.44E-06 | -0.0153 | 0.0069 | 0.02535 |
| ALM | TRAIL | rs79287178 | -0.4304 | 0.042 | 1.17E-24 | 0.0126 | 0.0057 | 0.02666 |
| ALM | VEGF | rs10411345 | -0.1041 | 0.0218 | 1.73E-06 | 0.005 | 0.0025 | 0.04335 |
| ALM | VEGF | rs10761731 | -0.1146 | 0.0174 | 4.31E-11 | -0.0041 | 0.0019 | 0.03053 |
| ALM | VEGF | rs10934631 | -0.1132 | 0.0244 | 3.61E-06 | 5.00E-04 | 0.0024 | 0.828 |
| ALM | VEGF | rs10967186 | 0.0899 | 0.0169 | 1.09E-07 | 9.00E-04 | 0.0019 | 0.6162 |
| ALM | VEGF | rs12456390 | -0.0818 | 0.0179 | 4.88E-06 | -0.0022 | 0.002 | 0.289 |
| ALM | VEGF | rs13209117 | 0.1253 | 0.02 | 3.70E-10 | 0.0044 | 0.0021 | 0.03778 |
| ALM | VEGF | rs143479231 | -0.2628 | 0.0489 | 7.90E-08 | 0.0083 | 0.0073 | 0.2523 |
| ALM | VEGF | rs3108686 | -0.7967 | 0.1702 | 2.86E-06 | 1.00E-04 | 0.0079 | 0.9888 |
| ALM | VEGF | rs4082730 | 0.2455 | 0.0533 | 4.12E-06 | -0.0032 | 0.0052 | 0.5399 |
| ALM | VEGF | rs6921438 | -0.4866 | 0.0174 | 4.11E-172 | 2.00E-04 | 0.0019 | 0.927 |
| ALM | VEGF | rs7030781 | 0.1403 | 0.0172 | 3.45E-16 | 7.00E-04 | 0.0019 | 0.7198 |
| ALM | VEGF | rs73418463 | -0.2498 | 0.0521 | 1.61E-06 | 1.00E-04 | 0.0042 | 0.9861 |
| ALM | VEGF | rs73872715 | -0.6079 | 0.1299 | 2.86E-06 | 0.0029 | 0.0079 | 0.709601 |
| ALM | VEGF | rs8045833 | 0.103 | 0.0211 | 1.01E-06 | -0.002 | 0.0021 | 0.3517 |
| ALM | VEGF | rs9472183 | -0.1264 | 0.017 | 9.54E-14 | -0.0052 | 0.002 | 0.008474 |
