# Supplementary material for: Causal roles of circulating cytokines in sarcopenia-related traits: a Mendelian randomization study
Source: Front Endocrinol (Lausanne). 2024 Sep 13;15:1370985. doi: 10.3389/fendo.2024.1370985 (PMC11427268; doi:10.3389/fendo.2024.1370985)
Supplement: Supplementary file 8 [file Table2.docx]

Supplemental table 2. Genome-wide significant SNPs used as IVs to investigate causal relationships between circulating cytokines and hand grip strength.

| **Outcome** | **Exposure** | **SNP** | **Exposure** | | | **Outcome** | | |
| --- | --- | --- | --- | --- | --- | --- | --- | --- |
|  |  |  | **Beta** | **Se** | **P-value** | **Beta** | **Se** | **P-value** |
| Hand grip strength (right) | B_NGF | rs28637706 | -0.1554 | 0.0261 | 2.72E-09 | -0.00245266 | 0.001533 | 0.11 |
| Hand grip strength (right) | B_NGF | rs71641308 | 0.1969 | 0.0429 | 4.42E-06 | 0.00508103 | 0.002831 | 0.073 |
| Hand grip strength (right) | B_NGF | rs73472576 | -0.1146 | 0.0251 | 4.81E-06 | 1.86E-04 | 0.001544 | 0.9 |
| Hand grip strength (right) | B_NGF | rs7970581 | 0.1358 | 0.028 | 1.22E-06 | 6.77E-04 | 0.001745 | 0.7 |
| Hand grip strength (right) | CTACK | rs116303454 | 0.3754 | 0.081 | 3.58E-06 | -0.00582957 | 0.005112 | 0.25 |
| Hand grip strength (right) | CTACK | rs118084576 | 0.5675 | 0.1226 | 3.66E-06 | 0.0100444 | 0.006894 | 0.15 |
| Hand grip strength (right) | CTACK | rs135564 | -0.1672 | 0.0267 | 3.59E-10 | 0.0012195 | 0.001641 | 0.46 |
| Hand grip strength (right) | CTACK | rs141331414 | 0.1977 | 0.0415 | 1.89E-06 | -0.00440071 | 0.00284 | 0.12 |
| Hand grip strength (right) | CTACK | rs2070074 | 0.4401 | 0.0372 | 2.60E-32 | -0.00329831 | 0.002526 | 0.19 |
| Hand grip strength (right) | CTACK | rs55764737 | 0.5424 | 0.0967 | 2.01E-08 | 0.00227688 | 0.004017 | 0.57 |
| Hand grip strength (right) | CTACK | rs57338032 | 0.1443 | 0.0316 | 4.83E-06 | -0.00342066 | 0.002014 | 0.089 |
| Hand grip strength (right) | CTACK | rs57789542 | -0.7687 | 0.1659 | 3.58E-06 | -0.00594984 | 0.005177 | 0.25 |
| Hand grip strength (right) | CTACK | rs60247384 | 0.1128 | 0.0245 | 4.30E-06 | -9.25E-04 | 0.001715 | 0.59 |
| Hand grip strength (right) | CTACK | rs62578137 | -0.1311 | 0.0286 | 4.66E-06 | -0.00148163 | 0.001941 | 0.450001 |
| Hand grip strength (right) | CTACK | rs72729450 | -0.5123 | 0.1094 | 2.81E-06 | -0.00136986 | 0.006099 | 0.82 |
| Hand grip strength (right) | CTACK | rs7333764 | 0.2811 | 0.0591 | 2.00E-06 | 0.00206047 | 0.005337 | 0.7 |
| Hand grip strength (right) | CTACK | rs76395525 | 0.5193 | 0.1081 | 1.55E-06 | 0.00542613 | 0.006584 | 0.41 |
| Hand grip strength (right) | EOTAXIN | rs11087905 | 0.0954 | 0.0188 | 4.07E-07 | 0.00186384 | 0.001775 | 0.29 |
| Hand grip strength (right) | EOTAXIN | rs112347425 | 0.1595 | 0.0276 | 7.77E-09 | 0.00184562 | 0.002523 | 0.46 |
| Hand grip strength (right) | EOTAXIN | rs12075 | 0.1692 | 0.0155 | 1.21E-27 | 4.21E-04 | 0.001503 | 0.780001 |
| Hand grip strength (right) | EOTAXIN | rs147287945 | -0.1512 | 0.0313 | 1.36E-06 | -0.00812571 | 0.002782 | 0.0035 |
| Hand grip strength (right) | EOTAXIN | rs187131 | 0.1264 | 0.0253 | 5.74E-07 | -0.00574506 | 0.00225 | 0.011 |
| Hand grip strength (right) | EOTAXIN | rs2024050 | 0.164 | 0.0302 | 5.47E-08 | -1.52E-04 | 0.002435 | 0.95 |
| Hand grip strength (right) | EOTAXIN | rs2027855 | 0.0743 | 0.0162 | 4.27E-06 | -0.00312632 | 0.001577 | 0.047 |
| Hand grip strength (right) | EOTAXIN | rs2211994 | 0.0876 | 0.0177 | 6.98E-07 | 9.36E-04 | 0.001691 | 0.58 |
| Hand grip strength (right) | EOTAXIN | rs2228467 | -0.4154 | 0.0291 | 3.47E-46 | -0.00171067 | 0.003089 | 0.58 |
| Hand grip strength (right) | EOTAXIN | rs5754733 | -0.105 | 0.0213 | 8.20E-07 | -0.00113719 | 0.001773 | 0.52 |
| Hand grip strength (right) | EOTAXIN | rs59808887 | -0.1698 | 0.0356 | 1.89E-06 | 6.41E-04 | 0.002797 | 0.82 |
| Hand grip strength (right) | EOTAXIN | rs745331 | -0.0821 | 0.0176 | 3.04E-06 | 7.70E-04 | 0.001631 | 0.64 |
| Hand grip strength (right) | EOTAXIN | rs75426604 | -0.1371 | 0.0291 | 2.40E-06 | 0.00502743 | 0.002199 | 0.022 |
| Hand grip strength (right) | EOTAXIN | rs79722574 | -0.1092 | 0.0227 | 1.50E-06 | -0.00152675 | 0.002034 | 0.450001 |
| Hand grip strength (right) | EOTAXIN | rs9317045 | 0.1172 | 0.0236 | 6.95E-07 | -8.82E-04 | 0.00203 | 0.66 |
| Hand grip strength (right) | FGF_BASIC | rs116745220 | -0.6176 | 0.1324 | 3.09E-06 | 0.00498826 | 0.005801 | 0.39 |
| Hand grip strength (right) | FGF_BASIC | rs13412535 | -0.1129 | 0.0224 | 4.76E-07 | -4.17E-04 | 0.001763 | 0.81 |
| Hand grip strength (right) | FGF_BASIC | rs145577605 | 0.2043 | 0.0427 | 1.67E-06 | 0.00968706 | 0.00958 | 0.31 |
| Hand grip strength (right) | FGF_BASIC | rs61990749 | 0.1124 | 0.0228 | 8.23E-07 | 0.00220179 | 0.002348 | 0.35 |
| Hand grip strength (right) | FGF_BASIC | rs75168112 | -0.1024 | 0.0214 | 1.64E-06 | -0.00329486 | 0.002257 | 0.14 |
| Hand grip strength (right) | FGF_BASIC | rs78873483 | 0.1286 | 0.0282 | 4.98E-06 | 0.00439387 | 0.00241 | 0.068 |
| Hand grip strength (right) | FGF_BASIC | rs9903590 | 0.1281 | 0.0267 | 1.62E-06 | -9.14E-04 | 0.00253 | 0.719999 |
| Hand grip strength (right) | GROA | rs1113500 | 0.1162 | 0.0243 | 1.72E-06 | 0.00115443 | 0.001547 | 0.46 |
| Hand grip strength (right) | GROA | rs118158560 | 0.2761 | 0.0592 | 3.09E-06 | -0.00711437 | 0.003165 | 0.025 |
| Hand grip strength (right) | GROA | rs12075 | 0.3724 | 0.0236 | 3.46E-56 | 4.21E-04 | 0.001503 | 0.780001 |
| Hand grip strength (right) | GROA | rs140734053 | 0.7333 | 0.1545 | 2.07E-06 | -0.0146383 | 0.005725 | 0.011 |
| Hand grip strength (right) | GROA | rs185768063 | 0.4038 | 0.076 | 1.06E-07 | 0.00345586 | 0.008027 | 0.67 |
| Hand grip strength (right) | GROA | rs188345231 | 0.6177 | 0.1322 | 2.97E-06 | -0.00135371 | 0.00545 | 0.8 |
| Hand grip strength (right) | GROA | rs508977 | -0.3838 | 0.0279 | 4.57E-43 | -0.00124742 | 0.001741 | 0.47 |
| Hand grip strength (right) | GROA | rs62024303 | -0.3013 | 0.066 | 4.91E-06 | 0.00109698 | 0.003656 | 0.760001 |
| Hand grip strength (right) | GROA | rs76390238 | 0.6223 | 0.1352 | 4.14E-06 | -0.0010426 | 0.005263 | 0.84 |
| Hand grip strength (right) | GROA | rs78653452 | -0.7395 | 0.1559 | 2.09E-06 | 0.00385652 | 0.006636 | 0.56 |
| Hand grip strength (right) | G_CSF | rs115256310 | -0.6788 | 0.1359 | 5.85E-07 | 0.00502874 | 0.0058 | 0.39 |
| Hand grip strength (right) | G_CSF | rs117261691 | 0.1318 | 0.0288 | 4.67E-06 | -0.00240185 | 0.005471 | 0.66 |
| Hand grip strength (right) | G_CSF | rs11903143 | 0.0889 | 0.0175 | 3.78E-07 | -3.74E-04 | 0.001666 | 0.82 |
| Hand grip strength (right) | G_CSF | rs145756094 | -0.7323 | 0.1479 | 7.40E-07 | -0.00406135 | 0.005901 | 0.49 |
| Hand grip strength (right) | G_CSF | rs2671444 | -0.0776 | 0.0166 | 2.86E-06 | 0.00196589 | 0.001547 | 0.2 |
| Hand grip strength (right) | G_CSF | rs586313 | -0.0883 | 0.0187 | 2.36E-06 | -0.00140217 | 0.001714 | 0.41 |
| Hand grip strength (right) | G_CSF | rs74148555 | -0.3771 | 0.0753 | 5.59E-07 | 2.78E-04 | 0.004305 | 0.95 |
| Hand grip strength (right) | G_CSF | rs76287671 | 0.0894 | 0.0189 | 2.19E-06 | -0.00463293 | 0.001904 | 0.015 |
| Hand grip strength (right) | G_CSF | rs77318030 | -0.2031 | 0.0427 | 2.02E-06 | -0.00699594 | 0.00333 | 0.036 |
| Hand grip strength (right) | HGF | rs11060254 | -0.0765 | 0.0166 | 3.97E-06 | -0.00119572 | 0.00156 | 0.44 |
| Hand grip strength (right) | HGF | rs13412535 | -0.1043 | 0.0213 | 9.67E-07 | -4.17E-04 | 0.001763 | 0.81 |
| Hand grip strength (right) | HGF | rs1617833 | -0.0749 | 0.016 | 2.93E-06 | 5.39E-04 | 0.001515 | 0.719999 |
| Hand grip strength (right) | HGF | rs180840563 | -0.2022 | 0.0416 | 1.15E-06 | -0.0108508 | 0.005108 | 0.034 |
| Hand grip strength (right) | HGF | rs2003620 | 0.2277 | 0.0487 | 2.98E-06 | -0.00226723 | 0.003251 | 0.49 |
| Hand grip strength (right) | HGF | rs3748034 | 0.1529 | 0.0233 | 5.21E-11 | -0.00375702 | 0.00213 | 0.077999 |
| Hand grip strength (right) | HGF | rs4245058 | -0.1552 | 0.0331 | 2.68E-06 | 5.91E-04 | 0.00254 | 0.82 |
| Hand grip strength (right) | HGF | rs5745687 | -0.3008 | 0.0404 | 9.92E-14 | -0.00290288 | 0.002993 | 0.33 |
| Hand grip strength (right) | IFN_G | rs10481651 | -0.0793 | 0.0168 | 2.18E-06 | -0.00277294 | 0.001595 | 0.081999 |
| Hand grip strength (right) | IFN_G | rs10761731 | -0.0813 | 0.0167 | 1.07E-06 | 0.00423069 | 0.001508 | 0.005 |
| Hand grip strength (right) | IFN_G | rs115729819 | 0.2511 | 0.0514 | 1.05E-06 | -0.0140349 | 0.006173 | 0.023 |
| Hand grip strength (right) | IFN_G | rs11843756 | 0.1812 | 0.0391 | 3.62E-06 | 0.00507635 | 0.004619 | 0.27 |
| Hand grip strength (right) | IFN_G | rs12420286 | 0.2357 | 0.05 | 2.45E-06 | -0.0025809 | 0.00368 | 0.48 |
| Hand grip strength (right) | IFN_G | rs2073438 | 0.092 | 0.0188 | 9.55E-07 | 4.23E-04 | 0.001636 | 0.8 |
| Hand grip strength (right) | IFN_G | rs2188420 | 0.1005 | 0.0201 | 5.90E-07 | 0.00390025 | 0.00228 | 0.087 |
| Hand grip strength (right) | IFN_G | rs60059008 | 0.0852 | 0.0176 | 1.30E-06 | -0.00332655 | 0.001578 | 0.035 |
| Hand grip strength (right) | IFN_G | rs73479333 | -0.1123 | 0.024 | 2.82E-06 | -0.00256598 | 0.002812 | 0.36 |
| Hand grip strength (right) | IFN_G | rs74148555 | -0.3771 | 0.077 | 9.86E-07 | 2.78E-04 | 0.004305 | 0.95 |
| Hand grip strength (right) | IFN_G | rs78296352 | 0.3419 | 0.065 | 1.42E-07 | 0.00288067 | 0.003839 | 0.450001 |
| Hand grip strength (right) | IL_10 | rs10457128 | -0.0854 | 0.0172 | 6.96E-07 | -0.00159927 | 0.001548 | 0.3 |
| Hand grip strength (right) | IL_10 | rs10493718 | -0.1081 | 0.0222 | 1.07E-06 | 0.00305651 | 0.001773 | 0.085 |
| Hand grip strength (right) | IL_10 | rs10888839 | 0.1203 | 0.025 | 1.56E-06 | 4.20E-04 | 0.002764 | 0.88 |
| Hand grip strength (right) | IL_10 | rs1530455 | 0.082 | 0.0174 | 2.53E-06 | -0.00106894 | 0.00153 | 0.48 |
| Hand grip strength (right) | IL_10 | rs2086656 | -0.08 | 0.017 | 2.59E-06 | -0.00364156 | 0.00163 | 0.025 |
| Hand grip strength (right) | IL_10 | rs282258 | 0.0993 | 0.0162 | 8.63E-10 | 9.41E-04 | 0.001505 | 0.53 |
| Hand grip strength (right) | IL_10 | rs3002131 | 0.1191 | 0.026 | 4.59E-06 | -0.00293165 | 0.002315 | 0.21 |
| Hand grip strength (right) | IL_10 | rs3025021 | 0.0913 | 0.0194 | 2.61E-06 | 0.00358879 | 0.00162 | 0.027 |
| Hand grip strength (right) | IL_10 | rs339203 | 0.0954 | 0.0203 | 2.75E-06 | -7.24E-04 | 0.001845 | 0.69 |
| Hand grip strength (right) | IL_10 | rs383684 | 0.092 | 0.0197 | 3.17E-06 | -0.00100966 | 0.002373 | 0.67 |
| Hand grip strength (right) | IL_10 | rs41282660 | -0.1169 | 0.0254 | 4.23E-06 | 0.00130859 | 0.002322 | 0.57 |
| Hand grip strength (right) | IL_10 | rs6085948 | 0.0977 | 0.0202 | 1.28E-06 | -1.08E-04 | 0.001757 | 0.95 |
| Hand grip strength (right) | IL_10 | rs6799107 | -0.095 | 0.0206 | 3.99E-06 | 0.00207384 | 0.001829 | 0.26 |
| Hand grip strength (right) | IL_10 | rs6921438 | -0.2876 | 0.0166 | 1.38E-67 | 0.00120347 | 0.001493 | 0.42 |
| Hand grip strength (right) | IL_10 | rs7088799 | -0.0815 | 0.0166 | 9.35E-07 | 0.0042955 | 0.001508 | 0.0044 |
| Hand grip strength (right) | IL_12_P70 | rs10761731 | -0.0965 | 0.0161 | 2.12E-09 | 0.00423069 | 0.001508 | 0.005 |
| Hand grip strength (right) | IL_12_P70 | rs13209117 | 0.0981 | 0.0186 | 1.27E-07 | 0.00263739 | 0.001674 | 0.12 |
| Hand grip strength (right) | IL_12_P70 | rs2123852 | 0.0942 | 0.0204 | 3.73E-06 | -0.00461741 | 0.002089 | 0.027 |
| Hand grip strength (right) | IL_12_P70 | rs273702 | -0.127 | 0.027 | 2.52E-06 | 0.0019052 | 0.002302 | 0.41 |
| Hand grip strength (right) | IL_12_P70 | rs282258 | 0.0726 | 0.0156 | 3.28E-06 | 9.41E-04 | 0.001505 | 0.53 |
| Hand grip strength (right) | IL_12_P70 | rs34291323 | 0.0954 | 0.0198 | 1.49E-06 | -0.00431279 | 0.001553 | 0.0055 |
| Hand grip strength (right) | IL_12_P70 | rs41282644 | 0.1401 | 0.0303 | 3.74E-06 | 0.00490171 | 0.003599 | 0.17 |
| Hand grip strength (right) | IL_12_P70 | rs6532374 | -0.1033 | 0.0226 | 4.61E-06 | 0.0059877 | 0.001947 | 0.0021 |
| Hand grip strength (right) | IL_12_P70 | rs6921438 | -0.3784 | 0.016 | 5.78E-124 | 0.00120347 | 0.001493 | 0.42 |
| Hand grip strength (right) | IL_12_P70 | rs6993770 | 0.0918 | 0.0188 | 1.06E-06 | 0.00285946 | 0.001641 | 0.081001 |
| Hand grip strength (right) | IL_12_P70 | rs71361173 | 0.1105 | 0.0238 | 3.57E-06 | -9.00E-05 | 0.002149 | 0.97 |
| Hand grip strength (right) | IL_12_P70 | rs72831623 | 0.1929 | 0.0367 | 1.51E-07 | 0.00944038 | 0.003528 | 0.0075 |
| Hand grip strength (right) | IL_12_P70 | rs782107 | 0.0765 | 0.0156 | 9.13E-07 | 0.00166066 | 0.001497 | 0.27 |
| Hand grip strength (right) | IL_12_P70 | rs9472183 | -0.1006 | 0.0157 | 1.38E-10 | -0.002858 | 0.001555 | 0.065999 |
| Hand grip strength (right) | IL_13 | rs10995615 | -0.1591 | 0.0341 | 3.12E-06 | 0.00157492 | 0.001892 | 0.41 |
| Hand grip strength (right) | IL_13 | rs117795020 | -0.3584 | 0.0716 | 5.48E-07 | 0.00482186 | 0.005552 | 0.39 |
| Hand grip strength (right) | IL_13 | rs12623722 | -0.1189 | 0.0257 | 3.61E-06 | -0.00210066 | 0.001633 | 0.2 |
| Hand grip strength (right) | IL_13 | rs139083458 | 0.9995 | 0.211 | 2.17E-06 | 0.00673889 | 0.005689 | 0.24 |
| Hand grip strength (right) | IL_13 | rs27949 | -0.1144 | 0.025 | 4.83E-06 | -0.00153828 | 0.001598 | 0.34 |
| Hand grip strength (right) | IL_13 | rs6799107 | -0.1472 | 0.0299 | 8.66E-07 | 0.00207384 | 0.001829 | 0.26 |
| Hand grip strength (right) | IL_13 | rs6921438 | -0.4139 | 0.0242 | 1.28E-65 | 0.00120347 | 0.001493 | 0.42 |
| Hand grip strength (right) | IL_13 | rs7073807 | 0.1618 | 0.0354 | 4.77E-06 | -0.00539006 | 0.002372 | 0.023 |
| Hand grip strength (right) | IL_13 | rs75383097 | -0.5369 | 0.116 | 3.70E-06 | -0.00684819 | 0.00639 | 0.28 |
| Hand grip strength (right) | IL_13 | rs76339001 | -0.4375 | 0.0886 | 7.92E-07 | -1.36E-04 | 0.004681 | 0.98 |
| Hand grip strength (right) | IL_13 | rs77955971 | 0.4408 | 0.0868 | 3.76E-07 | -3.60E-04 | 0.00475 | 0.94 |
| Hand grip strength (right) | IL_16 | rs116135478 | 0.8296 | 0.1637 | 4.05E-07 | -0.00734537 | 0.005236 | 0.16 |
| Hand grip strength (right) | IL_16 | rs117217798 | -0.2064 | 0.044 | 2.77E-06 | 5.93E-04 | 0.003021 | 0.84 |
| Hand grip strength (right) | IL_16 | rs117916513 | -0.4713 | 0.0982 | 1.61E-06 | -0.00250674 | 0.006647 | 0.709999 |
| Hand grip strength (right) | IL_16 | rs1255143 | 0.1387 | 0.0241 | 8.53E-09 | 9.41E-04 | 0.0015 | 0.53 |
| Hand grip strength (right) | IL_16 | rs144691581 | 0.4929 | 0.0958 | 2.67E-07 | 0.00723154 | 0.006359 | 0.26 |
| Hand grip strength (right) | IL_16 | rs4253283 | 0.1506 | 0.026 | 7.22E-09 | -0.00103782 | 0.001598 | 0.52 |
| Hand grip strength (right) | IL_16 | rs4778636 | -0.7286 | 0.063 | 6.21E-31 | 0.00137209 | 0.002573 | 0.59 |
| Hand grip strength (right) | IL_17 | rs11640734 | -0.115 | 0.024 | 1.61E-06 | -0.00451126 | 0.002398 | 0.06 |
| Hand grip strength (right) | IL_17 | rs117556572 | -0.5256 | 0.1097 | 1.66E-06 | -0.00729764 | 0.005691 | 0.2 |
| Hand grip strength (right) | IL_17 | rs12735700 | -0.0943 | 0.0206 | 4.50E-06 | 7.30E-04 | 0.001728 | 0.67 |
| Hand grip strength (right) | IL_17 | rs148562661 | 0.2161 | 0.0434 | 6.37E-07 | -0.00689192 | 0.005988 | 0.25 |
| Hand grip strength (right) | IL_17 | rs149738638 | -0.1553 | 0.0337 | 4.11E-06 | 3.65E-04 | 0.002874 | 0.9 |
| Hand grip strength (right) | IL_17 | rs1530455 | 0.1088 | 0.0173 | 3.29E-10 | -0.00106894 | 0.00153 | 0.48 |
| Hand grip strength (right) | IL_17 | rs17106604 | 0.1119 | 0.0225 | 6.23E-07 | 0.00204422 | 0.002365 | 0.39 |
| Hand grip strength (right) | IL_17 | rs17282552 | -0.2026 | 0.0403 | 4.88E-07 | 0.00519732 | 0.004202 | 0.22 |
| Hand grip strength (right) | IL_17 | rs78296352 | 0.2949 | 0.0645 | 4.81E-06 | 0.00288067 | 0.003839 | 0.450001 |
| Hand grip strength (right) | IL_17 | rs9568764 | 0.0825 | 0.018 | 4.68E-06 | -0.00140279 | 0.001663 | 0.4 |
| Hand grip strength (right) | IL_18 | rs116383510 | -0.5412 | 0.1052 | 2.70E-07 | -0.00364237 | 0.006537 | 0.58 |
| Hand grip strength (right) | IL_18 | rs117266781 | 0.7051 | 0.1436 | 9.18E-07 | 1.88E-04 | 0.006773 | 0.98 |
| Hand grip strength (right) | IL_18 | rs12420140 | -0.2479 | 0.0261 | 1.95E-21 | 2.91E-04 | 0.001648 | 0.86 |
| Hand grip strength (right) | IL_18 | rs143370787 | -0.3447 | 0.066 | 1.75E-07 | -0.00220326 | 0.002694 | 0.41 |
| Hand grip strength (right) | IL_18 | rs17229943 | -0.3076 | 0.0463 | 3.06E-11 | -0.00532 | 0.003393 | 0.12 |
| Hand grip strength (right) | IL_18 | rs1979967 | 0.14 | 0.0285 | 8.72E-07 | 3.74E-04 | 0.001773 | 0.83 |
| Hand grip strength (right) | IL_18 | rs385076 | -0.2472 | 0.0247 | 1.56E-23 | -0.0043353 | 0.001562 | 0.0055 |
| Hand grip strength (right) | IL_18 | rs4482818 | 0.1233 | 0.0243 | 4.11E-07 | -0.00247838 | 0.001541 | 0.11 |
| Hand grip strength (right) | IL_18 | rs610473 | 0.1274 | 0.0242 | 1.43E-07 | -0.00243922 | 0.001553 | 0.12 |
| Hand grip strength (right) | IL_18 | rs7444013 | -0.5318 | 0.0955 | 2.59E-08 | 0.00530421 | 0.006572 | 0.42 |
| Hand grip strength (right) | IL_18 | rs78623212 | 0.8322 | 0.1676 | 6.82E-07 | 0.00134575 | 0.004831 | 0.780001 |
| Hand grip strength (right) | IL_18 | rs78716465 | 0.3173 | 0.0679 | 2.98E-06 | 0.00487832 | 0.003902 | 0.21 |
| Hand grip strength (right) | IL_1B | rs115242021 | 0.2795 | 0.0553 | 4.25E-07 | 7.82E-04 | 0.003241 | 0.81 |
| Hand grip strength (right) | IL_1B | rs143319329 | 0.4357 | 0.093 | 2.84E-06 | 0.00409768 | 0.004696 | 0.38 |
| Hand grip strength (right) | IL_1B | rs61335305 | 0.4333 | 0.0928 | 3.02E-06 | 0.00129182 | 0.005567 | 0.82 |
| Hand grip strength (right) | IL_1B | rs62015704 | 0.1786 | 0.0372 | 1.62E-06 | 0.00204682 | 0.002257 | 0.36 |
| Hand grip strength (right) | IL_1RA | rs11627423 | 0.1178 | 0.0246 | 1.65E-06 | 3.91E-04 | 0.001518 | 0.8 |
| Hand grip strength (right) | IL_1RA | rs11869294 | -0.2286 | 0.047 | 1.13E-06 | 0.00231238 | 0.004324 | 0.59 |
| Hand grip strength (right) | IL_1RA | rs187166731 | -0.2424 | 0.0504 | 1.55E-06 | -3.46E-04 | 0.010964 | 0.97 |
| Hand grip strength (right) | IL_1RA | rs4441609 | 0.1056 | 0.0231 | 4.75E-06 | 5.47E-04 | 0.001533 | 0.719999 |
| Hand grip strength (right) | IL_1RA | rs61335305 | 0.4315 | 0.0904 | 1.81E-06 | 0.00129182 | 0.005567 | 0.82 |
| Hand grip strength (right) | IL_1RA | rs6699436 | -0.1858 | 0.0404 | 4.37E-06 | 6.97E-05 | 0.002131 | 0.97 |
| Hand grip strength (right) | IL_2 | rs13412535 | 0.174 | 0.0331 | 1.45E-07 | -4.17E-04 | 0.001763 | 0.81 |
| Hand grip strength (right) | IL_2 | rs16836080 | 0.1158 | 0.0253 | 4.84E-06 | 7.38E-04 | 0.001655 | 0.66 |
| Hand grip strength (right) | IL_2 | rs170117 | -0.1637 | 0.0347 | 2.44E-06 | 5.98E-04 | 0.002219 | 0.79 |
| Hand grip strength (right) | IL_2 | rs2690020 | 0.1158 | 0.0245 | 2.27E-06 | -0.00105596 | 0.001528 | 0.49 |
| Hand grip strength (right) | IL_2 | rs4634519 | -0.1249 | 0.0268 | 3.18E-06 | -0.00222542 | 0.001647 | 0.18 |
| Hand grip strength (right) | IL_2 | rs61335305 | 0.4439 | 0.0913 | 1.16E-06 | 0.00129182 | 0.005567 | 0.82 |
| Hand grip strength (right) | IL_2 | rs62124990 | -0.7013 | 0.149 | 2.50E-06 | -4.49E-04 | 0.004702 | 0.92 |
| Hand grip strength (right) | IL_2RA | rs11241559 | -0.124 | 0.0264 | 2.75E-06 | 0.00204482 | 0.001756 | 0.24 |
| Hand grip strength (right) | IL_2RA | rs117244812 | -0.7187 | 0.1493 | 1.47E-06 | -0.00786459 | 0.007019 | 0.26 |
| Hand grip strength (right) | IL_2RA | rs12722497 | 0.6287 | 0.0482 | 7.98E-39 | 0.00303937 | 0.002636 | 0.25 |
| Hand grip strength (right) | IL_2RA | rs12799226 | -0.1285 | 0.0277 | 3.56E-06 | 0.00352843 | 0.001836 | 0.055 |
| Hand grip strength (right) | IL_2RA | rs185231391 | 0.8568 | 0.1803 | 2.00E-06 | -0.00788398 | 0.006785 | 0.25 |
| Hand grip strength (right) | IL_2RA | rs28441585 | 0.1269 | 0.0271 | 2.93E-06 | 0.00392432 | 0.001784 | 0.028 |
| Hand grip strength (right) | IL_2RA | rs4733117 | 0.1439 | 0.0291 | 7.91E-07 | -0.00299673 | 0.002125 | 0.16 |
| Hand grip strength (right) | IL_4 | rs10512267 | -0.0824 | 0.016 | 2.73E-07 | -0.00308153 | 0.001588 | 0.052 |
| Hand grip strength (right) | IL_4 | rs116705532 | -0.4675 | 0.0978 | 1.73E-06 | -0.0065349 | 0.005861 | 0.26 |
| Hand grip strength (right) | IL_4 | rs117146485 | -0.2856 | 0.0625 | 4.95E-06 | -0.00788313 | 0.007504 | 0.29 |
| Hand grip strength (right) | IL_4 | rs12238729 | 0.5271 | 0.1096 | 1.51E-06 | -4.02E-04 | 0.006429 | 0.95 |
| Hand grip strength (right) | IL_4 | rs13106889 | -0.1186 | 0.0224 | 1.22E-07 | 0.00101719 | 0.002067 | 0.62 |
| Hand grip strength (right) | IL_4 | rs17713451 | 0.1255 | 0.0252 | 6.41E-07 | -0.00387579 | 0.00216 | 0.073 |
| Hand grip strength (right) | IL_4 | rs2073438 | 0.0847 | 0.0183 | 3.73E-06 | 4.23E-04 | 0.001636 | 0.8 |
| Hand grip strength (right) | IL_4 | rs58202480 | -0.0767 | 0.0166 | 3.59E-06 | -0.00100649 | 0.001859 | 0.59 |
| Hand grip strength (right) | IL_4 | rs6765768 | 0.0796 | 0.0167 | 1.85E-06 | 8.58E-04 | 0.001538 | 0.58 |
| Hand grip strength (right) | IL_4 | rs73023729 | -0.1796 | 0.0365 | 8.56E-07 | -0.00529277 | 0.005938 | 0.37 |
| Hand grip strength (right) | IL_4 | rs7613691 | 0.1787 | 0.0382 | 2.96E-06 | 1.07E-04 | 0.003059 | 0.97 |
| Hand grip strength (right) | IL_4 | rs79597994 | -0.5855 | 0.1271 | 4.06E-06 | -0.00473591 | 0.004783 | 0.32 |
| Hand grip strength (right) | IL_4 | rs9508291 | -0.168 | 0.0358 | 2.67E-06 | 0.00303243 | 0.002989 | 0.31 |
| Hand grip strength (right) | IL_4 | rs9941733 | 0.1156 | 0.0229 | 4.33E-07 | 3.80E-04 | 0.001986 | 0.85 |
| Hand grip strength (right) | IL_5 | rs11680908 | 0.2593 | 0.0552 | 2.62E-06 | -0.00682387 | 0.003033 | 0.024 |
| Hand grip strength (right) | IL_5 | rs148634917 | -0.517 | 0.1087 | 1.97E-06 | -0.00814374 | 0.005565 | 0.14 |
| Hand grip strength (right) | IL_5 | rs28793375 | 0.1697 | 0.0362 | 2.75E-06 | 6.67E-04 | 0.002219 | 0.760001 |
| Hand grip strength (right) | IL_5 | rs6737109 | 0.1135 | 0.0246 | 3.81E-06 | 0.00109726 | 0.001504 | 0.47 |
| Hand grip strength (right) | IL_5 | rs72831687 | -0.5337 | 0.1104 | 1.32E-06 | -0.00405399 | 0.008273 | 0.62 |
| Hand grip strength (right) | IL_5 | rs73040130 | 0.2745 | 0.0525 | 1.71E-07 | -9.32E-04 | 0.00313 | 0.77 |
| Hand grip strength (right) | IL_5 | rs74811276 | 0.217 | 0.0471 | 4.08E-06 | -7.80E-04 | 0.002779 | 0.780001 |
| Hand grip strength (right) | IL_5 | rs9472168 | 0.1568 | 0.0253 | 5.42E-10 | -0.00148946 | 0.001511 | 0.32 |
| Hand grip strength (right) | IL_6 | rs10752777 | 0.1083 | 0.0235 | 4.17E-06 | -0.00182741 | 0.002744 | 0.51 |
| Hand grip strength (right) | IL_6 | rs10982213 | -0.0849 | 0.0176 | 1.35E-06 | -0.00274369 | 0.001775 | 0.12 |
| Hand grip strength (right) | IL_6 | rs113098456 | -0.1553 | 0.0339 | 4.64E-06 | 0.00302465 | 0.003037 | 0.32 |
| Hand grip strength (right) | IL_6 | rs1333040 | 0.0747 | 0.0157 | 1.99E-06 | -0.00113518 | 0.001507 | 0.450001 |
| Hand grip strength (right) | IL_6 | rs13412535 | -0.1186 | 0.0214 | 3.14E-08 | -4.17E-04 | 0.001763 | 0.81 |
| Hand grip strength (right) | IL_6 | rs2404476 | 0.0734 | 0.0156 | 2.68E-06 | -4.76E-04 | 0.001488 | 0.75 |
| Hand grip strength (right) | IL_6 | rs4684700 | -0.0747 | 0.0162 | 3.91E-06 | 0.00235829 | 0.001514 | 0.12 |
| Hand grip strength (right) | IL_6 | rs72831623 | 0.197 | 0.0369 | 9.29E-08 | 0.00944038 | 0.003528 | 0.0075 |
| Hand grip strength (right) | IL_6 | rs73273528 | 0.268 | 0.0553 | 1.25E-06 | -0.00169488 | 0.004051 | 0.68 |
| Hand grip strength (right) | IL_6 | rs75101555 | -0.3625 | 0.0781 | 3.44E-06 | -0.00426965 | 0.004724 | 0.37 |
| Hand grip strength (right) | IL_6 | rs76856708 | 0.336 | 0.0697 | 1.43E-06 | -1.45E-04 | 0.00396 | 0.97 |
| Hand grip strength (right) | IL_7 | rs10196226 | 0.1538 | 0.0327 | 2.50E-06 | -0.00217597 | 0.002296 | 0.34 |
| Hand grip strength (right) | IL_7 | rs115215018 | 0.5985 | 0.1308 | 4.76E-06 | -0.0134829 | 0.005644 | 0.017 |
| Hand grip strength (right) | IL_7 | rs117509142 | -0.3213 | 0.0684 | 2.60E-06 | -0.00193944 | 0.003977 | 0.630001 |
| Hand grip strength (right) | IL_7 | rs141425475 | -0.4801 | 0.1018 | 2.39E-06 | 0.00769191 | 0.004724 | 0.1 |
| Hand grip strength (right) | IL_7 | rs17091524 | 0.5092 | 0.1015 | 5.24E-07 | -2.65E-06 | 0.004366 | 1 |
| Hand grip strength (right) | IL_7 | rs1958987 | 0.1261 | 0.0263 | 1.60E-06 | -5.96E-04 | 0.00159 | 0.709999 |
| Hand grip strength (right) | IL_7 | rs28793375 | 0.1644 | 0.036 | 4.87E-06 | 6.67E-04 | 0.002219 | 0.760001 |
| Hand grip strength (right) | IL_7 | rs62006410 | -0.1492 | 0.0302 | 7.59E-07 | 0.00223275 | 0.001874 | 0.23 |
| Hand grip strength (right) | IL_7 | rs6921438 | -0.3204 | 0.0246 | 8.71E-39 | 0.00120347 | 0.001493 | 0.42 |
| Hand grip strength (right) | IL_7 | rs77981494 | -0.5201 | 0.1055 | 8.23E-07 | 0.007021 | 0.005223 | 0.18 |
| Hand grip strength (right) | IL_8 | rs116726256 | -0.2247 | 0.0489 | 4.26E-06 | 0.0112017 | 0.005908 | 0.058 |
| Hand grip strength (right) | IL_8 | rs12075 | 0.1148 | 0.0235 | 9.97E-07 | 4.21E-04 | 0.001503 | 0.780001 |
| Hand grip strength (right) | IL_8 | rs12438669 | -0.1182 | 0.0252 | 2.60E-06 | -0.00120267 | 0.001551 | 0.44 |
| Hand grip strength (right) | IL_8 | rs141926526 | -0.6221 | 0.1308 | 1.96E-06 | -0.00855061 | 0.003822 | 0.025 |
| Hand grip strength (right) | IL_8 | rs2673604 | -0.118 | 0.0254 | 3.29E-06 | -0.00166718 | 0.001653 | 0.31 |
| Hand grip strength (right) | IL_8 | rs3786107 | 0.2463 | 0.0517 | 1.94E-06 | 0.00426781 | 0.003281 | 0.19 |
| Hand grip strength (right) | IL_8 | rs75840288 | 0.5125 | 0.1121 | 4.85E-06 | -0.00156921 | 0.003887 | 0.69 |
| Hand grip strength (right) | IL_9 | rs117807175 | -0.5225 | 0.1106 | 2.33E-06 | -0.00596256 | 0.004569 | 0.19 |
| Hand grip strength (right) | IL_9 | rs3736858 | -0.1351 | 0.0291 | 3.37E-06 | -0.00100276 | 0.002079 | 0.630001 |
| Hand grip strength (right) | IL_9 | rs41294750 | 0.3442 | 0.0736 | 2.92E-06 | -0.00502311 | 0.004454 | 0.26 |
| Hand grip strength (right) | IL_9 | rs73443903 | 0.2162 | 0.046 | 2.57E-06 | -7.78E-04 | 0.00258 | 0.760001 |
| Hand grip strength (right) | IL_9 | rs76963786 | -0.2856 | 0.0556 | 2.78E-07 | -0.00566181 | 0.00257 | 0.028 |
| Hand grip strength (right) | IP_10 | rs113831257 | 0.3639 | 0.0641 | 1.39E-08 | 0.00135638 | 0.00383 | 0.719999 |
| Hand grip strength (right) | IP_10 | rs143799975 | -0.7551 | 0.1638 | 4.01E-06 | -0.00841326 | 0.006882 | 0.22 |
| Hand grip strength (right) | IP_10 | rs34383175 | -0.3196 | 0.0653 | 9.90E-07 | 7.50E-04 | 0.004009 | 0.85 |
| Hand grip strength (right) | IP_10 | rs397816 | 0.1211 | 0.0248 | 1.03E-06 | 0.00129645 | 0.001506 | 0.39 |
| Hand grip strength (right) | IP_10 | rs4862111 | 0.1448 | 0.0317 | 4.84E-06 | 0.0027046 | 0.00199 | 0.17 |
| Hand grip strength (right) | IP_10 | rs6707974 | 0.1574 | 0.0337 | 3.03E-06 | -0.00241859 | 0.002024 | 0.23 |
| Hand grip strength (right) | IP_10 | rs75970138 | -0.4845 | 0.1037 | 2.99E-06 | 0.00512038 | 0.008352 | 0.54 |
| Hand grip strength (right) | IP_10 | rs7645625 | -0.1116 | 0.0236 | 2.19E-06 | -0.00135064 | 0.001507 | 0.37 |
| Hand grip strength (right) | IP_10 | rs79848609 | 0.2514 | 0.0535 | 2.64E-06 | -0.0091616 | 0.003585 | 0.011 |
| Hand grip strength (right) | IP_10 | rs8112909 | -0.139 | 0.0297 | 2.96E-06 | -0.00207645 | 0.001856 | 0.26 |
| Hand grip strength (right) | IP_10 | rs9450351 | -0.2651 | 0.0488 | 5.48E-08 | 7.65E-04 | 0.003018 | 0.8 |
| Hand grip strength (right) | MCP_1_MCAF | rs10744620 | 0.0783 | 0.0161 | 1.12E-06 | -0.00146132 | 0.001539 | 0.34 |
| Hand grip strength (right) | MCP_1_MCAF | rs111995966 | 0.1428 | 0.0309 | 3.79E-06 | -0.00653544 | 0.005528 | 0.24 |
| Hand grip strength (right) | MCP_1_MCAF | rs12073356 | -0.1436 | 0.031 | 3.49E-06 | 5.15E-04 | 0.003041 | 0.87 |
| Hand grip strength (right) | MCP_1_MCAF | rs12075 | 0.2186 | 0.0154 | 1.36E-45 | 4.21E-04 | 0.001503 | 0.780001 |
| Hand grip strength (right) | MCP_1_MCAF | rs143815843 | -0.2049 | 0.0447 | 4.61E-06 | -0.00591541 | 0.008113 | 0.47 |
| Hand grip strength (right) | MCP_1_MCAF | rs146522229 | -0.5942 | 0.1161 | 3.09E-07 | 0.00685966 | 0.006754 | 0.31 |
| Hand grip strength (right) | MCP_1_MCAF | rs2036297 | 0.1182 | 0.016 | 1.30E-13 | 0.00170471 | 0.001564 | 0.28 |
| Hand grip strength (right) | MCP_1_MCAF | rs2288370 | -0.1036 | 0.0162 | 1.56E-10 | 6.40E-04 | 0.00154 | 0.68 |
| Hand grip strength (right) | MCP_1_MCAF | rs56212190 | 0.1799 | 0.0372 | 1.32E-06 | -0.00279001 | 0.003612 | 0.44 |
| Hand grip strength (right) | MCP_1_MCAF | rs7033586 | -0.22 | 0.0467 | 2.43E-06 | 0.0055253 | 0.015538 | 0.719999 |
| Hand grip strength (right) | MCP_1_MCAF | rs7197349 | 0.0971 | 0.0206 | 2.40E-06 | -7.89E-04 | 0.00226 | 0.73 |
| Hand grip strength (right) | MCP_1_MCAF | rs7632755 | 0.2984 | 0.0315 | 2.79E-21 | 0.00334598 | 0.002918 | 0.25 |
| Hand grip strength (right) | MCP_1_MCAF | rs9317045 | 0.1157 | 0.0235 | 8.43E-07 | -8.82E-04 | 0.00203 | 0.66 |
| Hand grip strength (right) | MCP_3 | rs10892381 | 0.2432 | 0.0473 | 2.69E-07 | -0.00314734 | 0.001651 | 0.056999 |
| Hand grip strength (right) | MCP_3 | rs117286643 | 0.6934 | 0.1474 | 2.54E-06 | -0.00560995 | 0.005885 | 0.34 |
| Hand grip strength (right) | MCP_3 | rs2838065 | -0.221 | 0.0479 | 3.92E-06 | 7.21E-04 | 0.0019 | 0.7 |
| Hand grip strength (right) | MCP_3 | rs28394764 | 0.597 | 0.1282 | 3.19E-06 | -0.00485418 | 0.003414 | 0.16 |
| Hand grip strength (right) | MCP_3 | rs3129806 | -0.1975 | 0.0433 | 4.98E-06 | 0.00138551 | 0.00154 | 0.37 |
| Hand grip strength (right) | MCP_3 | rs62492260 | -0.2802 | 0.0578 | 1.23E-06 | -0.00142263 | 0.002222 | 0.52 |
| Hand grip strength (right) | MIF | rs113218956 | -0.8789 | 0.1876 | 2.82E-06 | -0.00901696 | 0.011323 | 0.43 |
| Hand grip strength (right) | MIF | rs11551183 | 0.3666 | 0.0795 | 4.00E-06 | -0.0020406 | 0.003957 | 0.61 |
| Hand grip strength (right) | MIF | rs12594190 | 0.1321 | 0.0266 | 6.85E-07 | -0.0013683 | 0.001785 | 0.44 |
| Hand grip strength (right) | MIF | rs13142904 | -0.2232 | 0.0425 | 1.47E-07 | 0.00164133 | 0.002977 | 0.58 |
| Hand grip strength (right) | MIF | rs141009259 | -0.6194 | 0.1285 | 1.44E-06 | -0.011183 | 0.007157 | 0.12 |
| Hand grip strength (right) | MIF | rs2294689 | -0.1338 | 0.0287 | 3.04E-06 | -0.00699555 | 0.003373 | 0.038 |
| Hand grip strength (right) | MIF | rs2330634 | 0.1549 | 0.0249 | 4.57E-10 | -8.35E-04 | 0.00152 | 0.58 |
| Hand grip strength (right) | MIF | rs35890933 | 0.1676 | 0.0365 | 4.46E-06 | -2.29E-04 | 0.001862 | 0.9 |
| Hand grip strength (right) | MIF | rs3814097 | -0.1163 | 0.0251 | 3.55E-06 | -0.00112797 | 0.001535 | 0.46 |
| Hand grip strength (right) | MIF | rs78098071 | -0.4583 | 0.0915 | 5.51E-07 | 0.0117223 | 0.006929 | 0.091 |
| Hand grip strength (right) | MIG | rs111607343 | -0.5235 | 0.1119 | 2.93E-06 | -0.00503564 | 0.0043 | 0.24 |
| Hand grip strength (right) | MIG | rs11177248 | 0.3157 | 0.0667 | 2.22E-06 | 0.00192157 | 0.003035 | 0.53 |
| Hand grip strength (right) | MIG | rs117831247 | -0.8819 | 0.173 | 3.45E-07 | -0.00268726 | 0.006923 | 0.7 |
| Hand grip strength (right) | MIG | rs13143163 | 0.2735 | 0.0582 | 2.62E-06 | 0.00178775 | 0.00317 | 0.57 |
| Hand grip strength (right) | MIG | rs139010077 | 0.4337 | 0.0943 | 4.19E-06 | -0.00554781 | 0.006504 | 0.39 |
| Hand grip strength (right) | MIG | rs1796086 | -0.2172 | 0.04 | 5.62E-08 | 0.00169332 | 0.002637 | 0.52 |
| Hand grip strength (right) | MIG | rs62562991 | 0.6239 | 0.1259 | 7.24E-07 | -0.0015391 | 0.005455 | 0.780001 |
| Hand grip strength (right) | MIG | rs6679677 | 0.1628 | 0.0327 | 6.51E-07 | -0.00433964 | 0.002463 | 0.077999 |
| Hand grip strength (right) | MIG | rs77086208 | 0.327 | 0.0694 | 2.50E-06 | -0.00234722 | 0.005728 | 0.68 |
| Hand grip strength (right) | MIG | rs816960 | -0.1179 | 0.0242 | 1.15E-06 | 0.00297891 | 0.001815 | 0.1 |
| Hand grip strength (right) | MIP_1A | rs12690897 | 0.1215 | 0.026 | 3.07E-06 | 0.00102396 | 0.001704 | 0.55 |
| Hand grip strength (right) | MIP_1A | rs57786342 | 0.139 | 0.0283 | 8.91E-07 | 8.79E-05 | 0.001853 | 0.96 |
| Hand grip strength (right) | MIP_1A | rs60198979 | -0.2154 | 0.0455 | 2.22E-06 | 8.21E-04 | 0.002733 | 0.760001 |
| Hand grip strength (right) | MIP_1A | rs6900267 | -0.2472 | 0.0515 | 1.60E-06 | -2.75E-04 | 0.00348 | 0.94 |
| Hand grip strength (right) | MIP_1B | rs113010081 | -0.5799 | 0.0236 | 1.57E-133 | 8.27E-04 | 0.002352 | 0.73 |
| Hand grip strength (right) | MIP_1B | rs113877493 | -0.607 | 0.0217 | 3.67E-172 | 0.00390252 | 0.002733 | 0.15 |
| Hand grip strength (right) | MIP_1B | rs116237296 | 0.5284 | 0.1115 | 2.15E-06 | -0.00456715 | 0.007185 | 0.53 |
| Hand grip strength (right) | MIP_1B | rs117453826 | -0.5907 | 0.0591 | 1.53E-23 | -0.00754761 | 0.00589 | 0.2 |
| Hand grip strength (right) | MIP_1B | rs117657747 | 0.2089 | 0.0453 | 4.01E-06 | -0.00313044 | 0.003084 | 0.31 |
| Hand grip strength (right) | MIP_1B | rs141102180 | 0.3298 | 0.0392 | 3.75E-17 | -0.00824409 | 0.005783 | 0.15 |
| Hand grip strength (right) | MIP_1B | rs1437220 | 0.1437 | 0.0315 | 4.92E-06 | 0.00361322 | 0.003345 | 0.28 |
| Hand grip strength (right) | MIP_1B | rs1564708 | -0.1697 | 0.0187 | 1.29E-19 | 9.40E-04 | 0.001882 | 0.62 |
| Hand grip strength (right) | MIP_1B | rs17138331 | -0.1434 | 0.0295 | 1.13E-06 | 0.00199078 | 0.002309 | 0.39 |
| Hand grip strength (right) | MIP_1B | rs2411161 | 0.1719 | 0.0365 | 2.55E-06 | 0.00276753 | 0.003284 | 0.4 |
| Hand grip strength (right) | MIP_1B | rs281748 | -0.0794 | 0.0171 | 3.28E-06 | 0.00364003 | 0.001603 | 0.023 |
| Hand grip strength (right) | MIP_1B | rs3760440 | 0.1242 | 0.0162 | 1.73E-14 | 4.85E-04 | 0.001573 | 0.760001 |
| Hand grip strength (right) | MIP_1B | rs6908843 | 0.0997 | 0.0209 | 1.78E-06 | -0.00161808 | 0.001928 | 0.4 |
| Hand grip strength (right) | MIP_1B | rs72791296 | 0.2364 | 0.0466 | 3.97E-07 | 2.81E-05 | 0.003606 | 0.99 |
| Hand grip strength (right) | MIP_1B | rs72799710 | -0.1037 | 0.0217 | 1.79E-06 | 0.00161763 | 0.001921 | 0.4 |
| Hand grip strength (right) | MIP_1B | rs76356863 | -0.3456 | 0.0667 | 2.22E-07 | -0.00161182 | 0.004379 | 0.709999 |
| Hand grip strength (right) | MIP_1B | rs76582507 | 0.3259 | 0.0676 | 1.42E-06 | -0.00216303 | 0.005839 | 0.709999 |
| Hand grip strength (right) | MIP_1B | rs76776296 | 0.313 | 0.0598 | 1.63E-07 | -0.00271062 | 0.003859 | 0.48 |
| Hand grip strength (right) | MIP_1B | rs79068918 | 0.2674 | 0.0271 | 5.54E-23 | 0.0032894 | 0.0025 | 0.19 |
| Hand grip strength (right) | M_CSF | rs116274860 | 0.8262 | 0.1739 | 2.03E-06 | 0.0108305 | 0.005899 | 0.065999 |
| Hand grip strength (right) | M_CSF | rs117867915 | 0.5224 | 0.1096 | 1.87E-06 | -0.00516603 | 0.007547 | 0.49 |
| Hand grip strength (right) | M_CSF | rs11963606 | -0.5353 | 0.117 | 4.73E-06 | 0.00620872 | 0.005922 | 0.29 |
| Hand grip strength (right) | M_CSF | rs12962919 | 0.3025 | 0.0659 | 4.39E-06 | -0.00208569 | 0.002459 | 0.4 |
| Hand grip strength (right) | M_CSF | rs34089869 | 0.2194 | 0.0462 | 2.08E-06 | 0.00377586 | 0.002359 | 0.11 |
| Hand grip strength (right) | M_CSF | rs4269021 | -0.2459 | 0.0504 | 1.05E-06 | 1.85E-04 | 0.002162 | 0.93 |
| Hand grip strength (right) | M_CSF | rs56367447 | -0.4878 | 0.0876 | 2.57E-08 | -0.00972089 | 0.00388 | 0.012 |
| Hand grip strength (right) | M_CSF | rs62294910 | 0.3472 | 0.0687 | 4.38E-07 | -0.00649649 | 0.003251 | 0.046 |
| Hand grip strength (right) | M_CSF | rs72723242 | -0.4969 | 0.1083 | 4.43E-06 | 0.00100427 | 0.00323 | 0.760001 |
| Hand grip strength (right) | M_CSF | rs78296352 | 0.522 | 0.111 | 2.58E-06 | 0.00288067 | 0.003839 | 0.450001 |
| Hand grip strength (right) | M_CSF | rs9387100 | -0.135 | 0.029 | 3.34E-06 | 0.00409515 | 0.001572 | 0.0092 |
| Hand grip strength (right) | PDGF_BB | rs11247305 | -0.1687 | 0.0364 | 3.47E-06 | -0.00165639 | 0.004043 | 0.68 |
| Hand grip strength (right) | PDGF_BB | rs116445074 | 0.2869 | 0.0587 | 1.02E-06 | 0.00105992 | 0.006142 | 0.86 |
| Hand grip strength (right) | PDGF_BB | rs11766649 | 0.0902 | 0.0196 | 3.96E-06 | -3.13E-04 | 0.001699 | 0.85 |
| Hand grip strength (right) | PDGF_BB | rs12289510 | -0.0772 | 0.0158 | 1.00E-06 | 0.00112927 | 0.001491 | 0.450001 |
| Hand grip strength (right) | PDGF_BB | rs13037046 | -0.0948 | 0.0206 | 3.96E-06 | -0.00141646 | 0.001764 | 0.42 |
| Hand grip strength (right) | PDGF_BB | rs13412535 | 0.3317 | 0.0214 | 2.89E-54 | -4.17E-04 | 0.001763 | 0.81 |
| Hand grip strength (right) | PDGF_BB | rs2324229 | 0.0884 | 0.0161 | 4.02E-08 | 0.00133235 | 0.001539 | 0.39 |
| Hand grip strength (right) | PDGF_BB | rs35859699 | -0.3854 | 0.0838 | 4.22E-06 | 0.00336332 | 0.00499 | 0.5 |
| Hand grip strength (right) | PDGF_BB | rs4965869 | 0.1843 | 0.0181 | 2.22E-24 | -0.00161371 | 0.001679 | 0.34 |
| Hand grip strength (right) | PDGF_BB | rs72777070 | -0.1048 | 0.02 | 1.56E-07 | 0.0029583 | 0.001926 | 0.12 |
| Hand grip strength (right) | PDGF_BB | rs73162807 | -0.2313 | 0.0499 | 3.55E-06 | 0.0043474 | 0.00516 | 0.4 |
| Hand grip strength (right) | PDGF_BB | rs9936075 | -0.0767 | 0.0163 | 2.68E-06 | -9.76E-05 | 0.001572 | 0.95 |
| Hand grip strength (right) | PDGF_BB | rs9941733 | 0.1165 | 0.0227 | 3.02E-07 | 3.80E-04 | 0.001986 | 0.85 |
| Hand grip strength (right) | RANTES | rs112072646 | 0.4209 | 0.0859 | 9.62E-07 | 0.00136404 | 0.004523 | 0.760001 |
| Hand grip strength (right) | RANTES | rs147509526 | -0.3558 | 0.0715 | 6.57E-07 | 0.00294846 | 0.006249 | 0.64 |
| Hand grip strength (right) | RANTES | rs2251660 | 0.1831 | 0.0356 | 2.69E-07 | -0.00221188 | 0.002088 | 0.29 |
| Hand grip strength (right) | RANTES | rs62438851 | -0.1904 | 0.0413 | 4.01E-06 | 0.00145463 | 0.002177 | 0.5 |
| Hand grip strength (right) | RANTES | rs7000423 | -0.1314 | 0.0252 | 1.85E-07 | -0.0014699 | 0.00159 | 0.36 |
| Hand grip strength (right) | RANTES | rs7170339 | -0.4283 | 0.0904 | 2.19E-06 | 0.00397256 | 0.005432 | 0.46 |
| Hand grip strength (right) | RANTES | rs72793342 | -0.1505 | 0.0307 | 9.08E-07 | 0.0059508 | 0.00186 | 0.0014 |
| Hand grip strength (right) | RANTES | rs74472919 | 0.3547 | 0.06 | 3.35E-09 | -0.00390611 | 0.004284 | 0.36 |
| Hand grip strength (right) | RANTES | rs9675798 | -0.2583 | 0.0552 | 2.89E-06 | 6.25E-04 | 0.00395 | 0.87 |
| Hand grip strength (right) | SCF | rs113127926 | 0.1974 | 0.0418 | 2.34E-06 | 0.00276929 | 0.002961 | 0.35 |
| Hand grip strength (right) | SCF | rs13412535 | -0.1065 | 0.0213 | 5.59E-07 | -4.17E-04 | 0.001763 | 0.81 |
| Hand grip strength (right) | SCF | rs1557570 | 0.1172 | 0.0169 | 4.13E-12 | 7.36E-04 | 0.001582 | 0.64 |
| Hand grip strength (right) | SCF | rs1568119 | -0.5946 | 0.1129 | 1.37E-07 | -0.00448441 | 0.007722 | 0.56 |
| Hand grip strength (right) | SCF | rs4841899 | -0.1002 | 0.0178 | 1.67E-08 | -0.00264743 | 0.001591 | 0.096 |
| Hand grip strength (right) | SCF | rs7039247 | 0.079 | 0.0168 | 2.46E-06 | 6.93E-04 | 0.001581 | 0.66 |
| Hand grip strength (right) | SCF | rs72678285 | 0.1062 | 0.0231 | 4.43E-06 | -0.0018311 | 0.002046 | 0.37 |
| Hand grip strength (right) | SCF | rs78369473 | -0.2256 | 0.0484 | 3.14E-06 | -0.00903435 | 0.004419 | 0.041 |
| Hand grip strength (right) | SCF | rs78666213 | -0.2845 | 0.0574 | 7.15E-07 | 0.00226204 | 0.004243 | 0.59 |
| Hand grip strength (right) | SCF | rs80271436 | -0.2393 | 0.0484 | 7.49E-07 | -0.00278851 | 0.003424 | 0.42 |
| Hand grip strength (right) | SCGF_B | rs112346514 | -0.3261 | 0.0703 | 3.54E-06 | -0.00473929 | 0.004421 | 0.28 |
| Hand grip strength (right) | SCGF_B | rs1149926 | -0.3458 | 0.0749 | 3.92E-06 | 0.00438457 | 0.004632 | 0.34 |
| Hand grip strength (right) | SCGF_B | rs116924815 | 0.6046 | 0.0737 | 2.25E-16 | -0.00140076 | 0.004465 | 0.75 |
| Hand grip strength (right) | SCGF_B | rs117716477 | 0.8242 | 0.084 | 1.03E-22 | 0.00738753 | 0.006351 | 0.24 |
| Hand grip strength (right) | SCGF_B | rs12480722 | 0.1654 | 0.0353 | 2.81E-06 | -0.00177913 | 0.00235 | 0.450001 |
| Hand grip strength (right) | SCGF_B | rs13287050 | -0.121 | 0.0263 | 4.12E-06 | -1.96E-04 | 0.001594 | 0.9 |
| Hand grip strength (right) | SCGF_B | rs139413256 | -0.5174 | 0.1076 | 1.53E-06 | 0.00573464 | 0.003931 | 0.14 |
| Hand grip strength (right) | SCGF_B | rs143829871 | -0.1866 | 0.0399 | 2.85E-06 | -0.00508162 | 0.003078 | 0.099001 |
| Hand grip strength (right) | SCGF_B | rs149009264 | 0.4551 | 0.0985 | 3.79E-06 | -0.0128251 | 0.006586 | 0.052 |
| Hand grip strength (right) | SCGF_B | rs150733161 | -0.5255 | 0.112 | 2.69E-06 | 0.00121292 | 0.006368 | 0.85 |
| Hand grip strength (right) | SCGF_B | rs151194174 | 0.4536 | 0.0941 | 1.45E-06 | 0.00716129 | 0.005244 | 0.17 |
| Hand grip strength (right) | SCGF_B | rs264157 | 0.1079 | 0.0233 | 3.69E-06 | -3.39E-04 | 0.001511 | 0.82 |
| Hand grip strength (right) | SCGF_B | rs34911860 | -0.3674 | 0.0787 | 3.00E-06 | -0.00608012 | 0.007687 | 0.43 |
| Hand grip strength (right) | SCGF_B | rs4656185 | 0.2103 | 0.0254 | 1.29E-16 | 9.77E-04 | 0.001584 | 0.54 |
| Hand grip strength (right) | SCGF_B | rs77954165 | 0.2631 | 0.0562 | 2.87E-06 | -0.0032536 | 0.002583 | 0.21 |
| Hand grip strength (right) | SCGF_B | rs7815967 | 0.1325 | 0.0288 | 4.37E-06 | 6.31E-04 | 0.002309 | 0.780001 |
| Hand grip strength (right) | SCGF_B | rs78217154 | 0.3942 | 0.0861 | 4.72E-06 | -0.00329917 | 0.005233 | 0.53 |
| Hand grip strength (right) | SDF_1A | rs10013755 | 0.5188 | 0.0995 | 1.85E-07 | -0.00551826 | 0.005436 | 0.31 |
| Hand grip strength (right) | SDF_1A | rs149893336 | -0.494 | 0.1082 | 4.93E-06 | -3.12E-04 | 0.005632 | 0.96 |
| Hand grip strength (right) | SDF_1A | rs1600396 | -0.0933 | 0.0204 | 4.94E-06 | 0.00321352 | 0.001711 | 0.06 |
| Hand grip strength (right) | SDF_1A | rs3988298 | -0.1263 | 0.0266 | 2.12E-06 | -7.76E-04 | 0.002269 | 0.73 |
| Hand grip strength (right) | SDF_1A | rs62194947 | -0.0852 | 0.0185 | 4.27E-06 | 0.00295984 | 0.001688 | 0.08 |
| Hand grip strength (right) | SDF_1A | rs78037609 | -0.6261 | 0.1334 | 2.67E-06 | 0.00465185 | 0.008731 | 0.59 |
| Hand grip strength (right) | TNF_A | rs10834997 | -0.123 | 0.0256 | 1.53E-06 | -6.28E-05 | 0.00161 | 0.97 |
| Hand grip strength (right) | TNF_A | rs111332265 | -0.3678 | 0.0745 | 7.91E-07 | -6.81E-04 | 0.00323 | 0.83 |
| Hand grip strength (right) | TNF_A | rs115669577 | 0.981 | 0.1994 | 8.63E-07 | -0.00736352 | 0.007788 | 0.34 |
| Hand grip strength (right) | TNF_A | rs7256693 | -0.1841 | 0.04 | 4.11E-06 | -0.00311873 | 0.002165 | 0.15 |
| Hand grip strength (right) | TNF_A | rs79105320 | 0.5573 | 0.1177 | 2.21E-06 | 0.00946385 | 0.005789 | 0.1 |
| Hand grip strength (right) | TNF_B | rs10925040 | 0.1738 | 0.0372 | 2.93E-06 | -0.00269576 | 0.00154 | 0.08 |
| Hand grip strength (right) | TNF_B | rs75240021 | 0.3713 | 0.0772 | 1.49E-06 | -1.73E-04 | 0.002846 | 0.95 |
| Hand grip strength (right) | TNF_B | rs753274 | -0.1725 | 0.037 | 3.14E-06 | -0.00231554 | 0.001497 | 0.12 |
| Hand grip strength (right) | TNF_B | rs7629875 | 0.3841 | 0.0774 | 6.90E-07 | -0.00179961 | 0.003371 | 0.59 |
| Hand grip strength (right) | TNF_B | rs78296352 | 1.2028 | 0.1366 | 1.28E-18 | 0.00288067 | 0.003839 | 0.450001 |
| Hand grip strength (right) | TRAIL | rs13278062 | 0.08 | 0.0157 | 3.33E-07 | -0.00145787 | 0.001488 | 0.33 |
| Hand grip strength (right) | TRAIL | rs138987090 | -0.7264 | 0.0749 | 2.97E-22 | -0.0177852 | 0.009194 | 0.053001 |
| Hand grip strength (right) | TRAIL | rs148051545 | -0.4211 | 0.0843 | 5.84E-07 | 0.00571837 | 0.0048 | 0.23 |
| Hand grip strength (right) | TRAIL | rs17434886 | -0.0918 | 0.0199 | 4.20E-06 | -0.00221131 | 0.002035 | 0.28 |
| Hand grip strength (right) | TRAIL | rs193112415 | -1.0456 | 0.062 | 1.01E-63 | -0.00390059 | 0.005494 | 0.48 |
| Hand grip strength (right) | TRAIL | rs28431810 | -0.1216 | 0.0252 | 1.41E-06 | 0.0056109 | 0.007994 | 0.48 |
| Hand grip strength (right) | TRAIL | rs28521641 | -0.7004 | 0.0445 | 7.79E-56 | 0.00201463 | 0.004143 | 0.630001 |
| Hand grip strength (right) | TRAIL | rs57396456 | -0.5641 | 0.0516 | 7.71E-28 | 0.00245739 | 0.004287 | 0.57 |
| Hand grip strength (right) | TRAIL | rs62093514 | 1.0459 | 0.0549 | 5.80E-81 | 0.00331304 | 0.00465 | 0.48 |
| Hand grip strength (right) | TRAIL | rs72899452 | 0.1223 | 0.0264 | 3.75E-06 | -0.00333193 | 0.003012 | 0.27 |
| Hand grip strength (right) | TRAIL | rs73039026 | -0.3098 | 0.0634 | 1.02E-06 | 5.71E-04 | 0.006863 | 0.93 |
| Hand grip strength (right) | TRAIL | rs747324 | -0.0826 | 0.0178 | 3.34E-06 | 1.10E-04 | 0.001594 | 0.95 |
| Hand grip strength (right) | TRAIL | rs74778900 | 0.5791 | 0.0531 | 9.90E-28 | -0.00253231 | 0.006214 | 0.68 |
| Hand grip strength (right) | TRAIL | rs75928541 | 0.2784 | 0.0591 | 2.44E-06 | -0.00582675 | 0.005401 | 0.28 |
| Hand grip strength (right) | TRAIL | rs79287178 | -0.4304 | 0.042 | 1.17E-24 | -0.00123471 | 0.004493 | 0.780001 |
| Hand grip strength (right) | VEGF | rs10411345 | -0.1041 | 0.0218 | 1.73E-06 | 0.00373796 | 0.001958 | 0.056 |
| Hand grip strength (right) | VEGF | rs10761731 | -0.1146 | 0.0174 | 4.31E-11 | 0.00423069 | 0.001508 | 0.005 |
| Hand grip strength (right) | VEGF | rs10934631 | -0.1132 | 0.0244 | 3.61E-06 | 0.00250948 | 0.001859 | 0.18 |
| Hand grip strength (right) | VEGF | rs10967186 | 0.0899 | 0.0169 | 1.09E-07 | -0.00181104 | 0.001488 | 0.22 |
| Hand grip strength (right) | VEGF | rs12456390 | -0.0818 | 0.0179 | 4.88E-06 | -5.20E-04 | 0.001596 | 0.74 |
| Hand grip strength (right) | VEGF | rs13209117 | 0.1253 | 0.02 | 3.70E-10 | 0.00263739 | 0.001674 | 0.12 |
| Hand grip strength (right) | VEGF | rs143479231 | -0.2628 | 0.0489 | 7.90E-08 | 0.00357394 | 0.005745 | 0.53 |
| Hand grip strength (right) | VEGF | rs3108686 | -0.7967 | 0.1702 | 2.86E-06 | -0.00524174 | 0.00619 | 0.4 |
| Hand grip strength (right) | VEGF | rs4082730 | 0.2455 | 0.0533 | 4.12E-06 | 7.95E-04 | 0.00406 | 0.84 |
| Hand grip strength (right) | VEGF | rs6921438 | -0.4866 | 0.0174 | 4.11E-172 | 0.00120347 | 0.001493 | 0.42 |
| Hand grip strength (right) | VEGF | rs7030781 | 0.1403 | 0.0172 | 3.45E-16 | -4.27E-04 | 0.001521 | 0.780001 |
| Hand grip strength (right) | VEGF | rs73418463 | -0.2498 | 0.0521 | 1.61E-06 | 3.17E-04 | 0.003301 | 0.92 |
| Hand grip strength (right) | VEGF | rs73872715 | -0.6079 | 0.1299 | 2.86E-06 | 0.00890155 | 0.006191 | 0.15 |
| Hand grip strength (right) | VEGF | rs8045833 | 0.103 | 0.0211 | 1.01E-06 | 2.65E-04 | 0.001675 | 0.87 |
| Hand grip strength (right) | VEGF | rs9472183 | -0.1264 | 0.017 | 9.54E-14 | -0.002858 | 0.001555 | 0.065999 |
