# Supplementary material for: Causal roles of circulating cytokines in sarcopenia-related traits: a Mendelian randomization study
Source: Front Endocrinol (Lausanne). 2024 Sep 13;15:1370985. doi: 10.3389/fendo.2024.1370985 (PMC11427268; doi:10.3389/fendo.2024.1370985)
Supplement: Supplementary file 9 [file Table3.docx]

Supplementary Table 3. Genome-wide significant SNPs as IVs used to investigate causal relationships between circulating cytokines and usual walking pace.

| **Outcome** | **Exposure** | **SNP** | **Exposure** | | | **Outcome** | | |
| --- | --- | --- | --- | --- | --- | --- | --- | --- |
|  |  |  | **Beta** | **Se** | **P-value** | **Beta** | **Se** | **P-value** |
| Usual walking pace | B_NGF | rs28637706 | -0.1554 | 0.0261 | 2.72E-09 | 0.00385149 | 0.0013046 | 0.0032 |
| Usual walking pace | B_NGF | rs71641308 | 0.1969 | 0.0429 | 4.42E-06 | -0.0058632 | 0.00241187 | 0.015 |
| Usual walking pace | B_NGF | rs73472576 | -0.1146 | 0.0251 | 4.81E-06 | -0.0013698 | 0.00131441 | 0.3 |
| Usual walking pace | B_NGF | rs7970581 | 0.1358 | 0.028 | 1.22E-06 | -0.0015022 | 0.00148558 | 0.31 |
| Usual walking pace | CTACK | rs116303454 | 0.3754 | 0.081 | 3.58E-06 | -0.0026945 | 0.00435357 | 0.54 |
| Usual walking pace | CTACK | rs118084576 | 0.5675 | 0.1226 | 3.66E-06 | 0.00486981 | 0.00587265 | 0.41 |
| Usual walking pace | CTACK | rs135564 | -0.1672 | 0.0267 | 3.59E-10 | 9.59E-04 | 0.00139632 | 0.49 |
| Usual walking pace | CTACK | rs141331414 | 0.1977 | 0.0415 | 1.89E-06 | -0.0031162 | 0.00241845 | 0.2 |
| Usual walking pace | CTACK | rs2070074 | 0.4401 | 0.0372 | 2.60E-32 | -0.0037377 | 0.00215053 | 0.081999 |
| Usual walking pace | CTACK | rs55764737 | 0.5424 | 0.0967 | 2.01E-08 | 0.00560608 | 0.00342083 | 0.1 |
| Usual walking pace | CTACK | rs57338032 | 0.1443 | 0.0316 | 4.83E-06 | 0.00238965 | 0.00171471 | 0.16 |
| Usual walking pace | CTACK | rs57789542 | -0.7687 | 0.1659 | 3.58E-06 | -0.0022771 | 0.00440925 | 0.61 |
| Usual walking pace | CTACK | rs60247384 | 0.1128 | 0.0245 | 4.30E-06 | -0.0015634 | 0.00146 | 0.28 |
| Usual walking pace | CTACK | rs62578137 | -0.1311 | 0.0286 | 4.66E-06 | 0.00171836 | 0.00165307 | 0.3 |
| Usual walking pace | CTACK | rs72729450 | -0.5123 | 0.1094 | 2.81E-06 | -0.0021918 | 0.00519347 | 0.67 |
| Usual walking pace | CTACK | rs7333764 | 0.2811 | 0.0591 | 2.00E-06 | 0.00461351 | 0.00454215 | 0.31 |
| Usual walking pace | CTACK | rs76395525 | 0.5193 | 0.1081 | 1.55E-06 | 0.00359315 | 0.00560486 | 0.52 |
| Usual walking pace | EOTAXIN | rs11087905 | 0.0954 | 0.0188 | 4.07E-07 | 0.00223711 | 0.00151055 | 0.14 |
| Usual walking pace | EOTAXIN | rs112347425 | 0.1595 | 0.0276 | 7.77E-09 | -5.45E-04 | 0.00214862 | 0.8 |
| Usual walking pace | EOTAXIN | rs12075 | 0.1692 | 0.0155 | 1.21E-27 | 0.00190711 | 0.0012804 | 0.14 |
| Usual walking pace | EOTAXIN | rs147287945 | -0.1512 | 0.0313 | 1.36E-06 | -1.98E-04 | 0.00236937 | 0.93 |
| Usual walking pace | EOTAXIN | rs187131 | 0.1264 | 0.0253 | 5.74E-07 | -0.0018828 | 0.00191716 | 0.33 |
| Usual walking pace | EOTAXIN | rs2024050 | 0.164 | 0.0302 | 5.47E-08 | -0.0018873 | 0.00207344 | 0.36 |
| Usual walking pace | EOTAXIN | rs2027855 | 0.0743 | 0.0162 | 4.27E-06 | 7.96E-04 | 0.0013421 | 0.55 |
| Usual walking pace | EOTAXIN | rs2211994 | 0.0876 | 0.0177 | 6.98E-07 | 0.00289187 | 0.0014393 | 0.045 |
| Usual walking pace | EOTAXIN | rs2228467 | -0.4154 | 0.0291 | 3.47E-46 | -0.0015748 | 0.00263065 | 0.55 |
| Usual walking pace | EOTAXIN | rs5754733 | -0.105 | 0.0213 | 8.20E-07 | 6.43E-04 | 0.00150886 | 0.67 |
| Usual walking pace | EOTAXIN | rs59808887 | -0.1698 | 0.0356 | 1.89E-06 | 0.00323941 | 0.00238229 | 0.17 |
| Usual walking pace | EOTAXIN | rs745331 | -0.0821 | 0.0176 | 3.04E-06 | -3.45E-04 | 0.00138944 | 0.8 |
| Usual walking pace | EOTAXIN | rs75426604 | -0.1371 | 0.0291 | 2.40E-06 | -6.46E-04 | 0.00187173 | 0.73 |
| Usual walking pace | EOTAXIN | rs79722574 | -0.1092 | 0.0227 | 1.50E-06 | -0.0016271 | 0.00173167 | 0.35 |
| Usual walking pace | EOTAXIN | rs9317045 | 0.1172 | 0.0236 | 6.95E-07 | -4.80E-04 | 0.00172738 | 0.780001 |
| Usual walking pace | FGF_BASIC | rs116745220 | -0.6176 | 0.1324 | 3.09E-06 | -0.0012558 | 0.00494154 | 0.8 |
| Usual walking pace | FGF_BASIC | rs13412535 | -0.1129 | 0.0224 | 4.76E-07 | -0.0012967 | 0.00150183 | 0.39 |
| Usual walking pace | FGF_BASIC | rs145577605 | 0.2043 | 0.0427 | 1.67E-06 | -0.0013779 | 0.00815236 | 0.87 |
| Usual walking pace | FGF_BASIC | rs61990749 | 0.1124 | 0.0228 | 8.23E-07 | -0.0027985 | 0.00199853 | 0.16 |
| Usual walking pace | FGF_BASIC | rs75168112 | -0.1024 | 0.0214 | 1.64E-06 | -0.0014426 | 0.00192054 | 0.450001 |
| Usual walking pace | FGF_BASIC | rs78873483 | 0.1286 | 0.0282 | 4.98E-06 | 0.00562093 | 0.0020515 | 0.0061 |
| Usual walking pace | FGF_BASIC | rs9903590 | 0.1281 | 0.0267 | 1.62E-06 | 8.82E-04 | 0.00215374 | 0.68 |
| Usual walking pace | GROA | rs1113500 | 0.1162 | 0.0243 | 1.72E-06 | -0.0018552 | 0.00131768 | 0.16 |
| Usual walking pace | GROA | rs118158560 | 0.2761 | 0.0592 | 3.09E-06 | 2.68E-04 | 0.00269477 | 0.92 |
| Usual walking pace | GROA | rs12075 | 0.3724 | 0.0236 | 3.46E-56 | 0.00190711 | 0.0012804 | 0.14 |
| Usual walking pace | GROA | rs140734053 | 0.7333 | 0.1545 | 2.07E-06 | 0.00216707 | 0.00487449 | 0.66 |
| Usual walking pace | GROA | rs185768063 | 0.4038 | 0.076 | 1.06E-07 | 0.00333231 | 0.00684048 | 0.630001 |
| Usual walking pace | GROA | rs188345231 | 0.6177 | 0.1322 | 2.97E-06 | 0.0025855 | 0.00464078 | 0.58 |
| Usual walking pace | GROA | rs508977 | -0.3838 | 0.0279 | 4.57E-43 | -0.0018354 | 0.00148258 | 0.22 |
| Usual walking pace | GROA | rs62024303 | -0.3013 | 0.066 | 4.91E-06 | -9.92E-04 | 0.00311234 | 0.75 |
| Usual walking pace | GROA | rs76390238 | 0.6223 | 0.1352 | 4.14E-06 | 9.65E-04 | 0.00448139 | 0.83 |
| Usual walking pace | GROA | rs78653452 | -0.7395 | 0.1559 | 2.09E-06 | 8.41E-04 | 0.00565175 | 0.88 |
| Usual walking pace | G_CSF | rs115256310 | -0.6788 | 0.1359 | 5.85E-07 | -0.0012242 | 0.00494113 | 0.8 |
| Usual walking pace | G_CSF | rs117261691 | 0.1318 | 0.0288 | 4.67E-06 | -0.0058962 | 0.00465915 | 0.21 |
| Usual walking pace | G_CSF | rs11903143 | 0.0889 | 0.0175 | 3.78E-07 | 6.93E-04 | 0.00141938 | 0.630001 |
| Usual walking pace | G_CSF | rs145756094 | -0.7323 | 0.1479 | 7.40E-07 | 0.00198141 | 0.00502434 | 0.69 |
| Usual walking pace | G_CSF | rs2671444 | -0.0776 | 0.0166 | 2.86E-06 | 5.43E-04 | 0.00131713 | 0.68 |
| Usual walking pace | G_CSF | rs586313 | -0.0883 | 0.0187 | 2.36E-06 | -3.84E-04 | 0.00145931 | 0.79 |
| Usual walking pace | G_CSF | rs74148555 | -0.3771 | 0.0753 | 5.59E-07 | -0.0023279 | 0.00366569 | 0.53 |
| Usual walking pace | G_CSF | rs76287671 | 0.0894 | 0.0189 | 2.19E-06 | 4.85E-04 | 0.00162034 | 0.760001 |
| Usual walking pace | G_CSF | rs77318030 | -0.2031 | 0.0427 | 2.02E-06 | 4.10E-04 | 0.00283375 | 0.88 |
| Usual walking pace | HGF | rs11060254 | -0.0765 | 0.0166 | 3.97E-06 | 1.33E-04 | 0.00132793 | 0.92 |
| Usual walking pace | HGF | rs13412535 | -0.1043 | 0.0213 | 9.67E-07 | -0.0012967 | 0.00150183 | 0.39 |
| Usual walking pace | HGF | rs1617833 | -0.0749 | 0.016 | 2.93E-06 | 0.00312237 | 0.0012903 | 0.016 |
| Usual walking pace | HGF | rs180840563 | -0.2022 | 0.0416 | 1.15E-06 | -0.0094039 | 0.00434908 | 0.031 |
| Usual walking pace | HGF | rs2003620 | 0.2277 | 0.0487 | 2.98E-06 | -5.94E-04 | 0.00276791 | 0.83 |
| Usual walking pace | HGF | rs3748034 | 0.1529 | 0.0233 | 5.21E-11 | -0.0057381 | 0.00181342 | 0.0016 |
| Usual walking pace | HGF | rs4245058 | -0.1552 | 0.0331 | 2.68E-06 | -1.91E-04 | 0.00216293 | 0.93 |
| Usual walking pace | HGF | rs5745687 | -0.3008 | 0.0404 | 9.92E-14 | -5.24E-04 | 0.00254953 | 0.84 |
| Usual walking pace | IFN_G | rs10481651 | -0.0793 | 0.0168 | 2.18E-06 | -2.06E-04 | 0.00135843 | 0.88 |
| Usual walking pace | IFN_G | rs113600793 | 0.1871 | 0.0371 | 4.43E-07 | 0.00973047 | 0.00334148 | 0.0036 |
| Usual walking pace | IFN_G | rs115729819 | 0.2511 | 0.0514 | 1.05E-06 | -0.0084904 | 0.0052631 | 0.11 |
| Usual walking pace | IFN_G | rs11843756 | 0.1812 | 0.0391 | 3.62E-06 | 0.00456372 | 0.00393369 | 0.25 |
| Usual walking pace | IFN_G | rs12420286 | 0.2357 | 0.05 | 2.45E-06 | -0.0059038 | 0.00313337 | 0.06 |
| Usual walking pace | IFN_G | rs2073438 | 0.092 | 0.0188 | 9.55E-07 | -2.06E-04 | 0.0013927 | 0.88 |
| Usual walking pace | IFN_G | rs2188420 | 0.1005 | 0.0201 | 5.90E-07 | 0.00147924 | 0.0019413 | 0.450001 |
| Usual walking pace | IFN_G | rs60059008 | 0.0852 | 0.0176 | 1.30E-06 | 1.02E-04 | 0.00134336 | 0.94 |
| Usual walking pace | IFN_G | rs73479333 | -0.1123 | 0.024 | 2.82E-06 | 0.00227901 | 0.00239567 | 0.34 |
| Usual walking pace | IFN_G | rs74148555 | -0.3771 | 0.077 | 9.86E-07 | -0.0023279 | 0.00366569 | 0.53 |
| Usual walking pace | IFN_G | rs78296352 | 0.3419 | 0.065 | 1.42E-07 | -0.0013505 | 0.00326992 | 0.68 |
| Usual walking pace | IL_10 | rs10457128 | -0.0854 | 0.0172 | 6.96E-07 | -2.22E-04 | 0.00131798 | 0.87 |
| Usual walking pace | IL_10 | rs10493718 | -0.1081 | 0.0222 | 1.07E-06 | 0.00231684 | 0.0015107 | 0.13 |
| Usual walking pace | IL_10 | rs10888839 | 0.1203 | 0.025 | 1.56E-06 | -9.83E-04 | 0.00235446 | 0.68 |
| Usual walking pace | IL_10 | rs1530455 | 0.082 | 0.0174 | 2.53E-06 | -0.001531 | 0.00130298 | 0.24 |
| Usual walking pace | IL_10 | rs2086656 | -0.08 | 0.017 | 2.59E-06 | -6.21E-04 | 0.00138778 | 0.649999 |
| Usual walking pace | IL_10 | rs282258 | 0.0993 | 0.0162 | 8.63E-10 | 0.0020613 | 0.00128175 | 0.11 |
| Usual walking pace | IL_10 | rs3002131 | 0.1191 | 0.026 | 4.59E-06 | -3.33E-04 | 0.00197179 | 0.87 |
| Usual walking pace | IL_10 | rs3025021 | 0.0913 | 0.0194 | 2.61E-06 | 0.00316954 | 0.00137986 | 0.022 |
| Usual walking pace | IL_10 | rs339203 | 0.0954 | 0.0203 | 2.75E-06 | 0.00232445 | 0.00157165 | 0.14 |
| Usual walking pace | IL_10 | rs383684 | 0.092 | 0.0197 | 3.17E-06 | -0.0033155 | 0.00202017 | 0.1 |
| Usual walking pace | IL_10 | rs41282660 | -0.1169 | 0.0254 | 4.23E-06 | -0.00108 | 0.00197796 | 0.59 |
| Usual walking pace | IL_10 | rs6085948 | 0.0977 | 0.0202 | 1.28E-06 | -2.29E-04 | 0.00149547 | 0.88 |
| Usual walking pace | IL_10 | rs6799107 | -0.095 | 0.0206 | 3.99E-06 | 0.00240672 | 0.00155774 | 0.12 |
| Usual walking pace | IL_10 | rs6921438 | -0.2876 | 0.0166 | 1.38E-67 | -9.08E-04 | 0.00127181 | 0.48 |
| Usual walking pace | IL_12_P70 | rs13209117 | 0.0981 | 0.0186 | 1.27E-07 | -8.83E-04 | 0.00142606 | 0.54 |
| Usual walking pace | IL_12_P70 | rs2123852 | 0.0942 | 0.0204 | 3.73E-06 | -0.0021135 | 0.00177789 | 0.23 |
| Usual walking pace | IL_12_P70 | rs273702 | -0.127 | 0.027 | 2.52E-06 | -7.47E-05 | 0.00195947 | 0.97 |
| Usual walking pace | IL_12_P70 | rs282258 | 0.0726 | 0.0156 | 3.28E-06 | 0.0020613 | 0.00128175 | 0.11 |
| Usual walking pace | IL_12_P70 | rs34291323 | 0.0954 | 0.0198 | 1.49E-06 | -0.0011999 | 0.00132216 | 0.36 |
| Usual walking pace | IL_12_P70 | rs41282644 | 0.1401 | 0.0303 | 3.74E-06 | 0.00256255 | 0.00306497 | 0.4 |
| Usual walking pace | IL_12_P70 | rs6532374 | -0.1033 | 0.0226 | 4.61E-06 | 0.0017703 | 0.00165849 | 0.29 |
| Usual walking pace | IL_12_P70 | rs6921438 | -0.3784 | 0.016 | 5.78E-124 | -9.08E-04 | 0.00127181 | 0.48 |
| Usual walking pace | IL_12_P70 | rs6993770 | 0.0918 | 0.0188 | 1.06E-06 | -0.0039647 | 0.00139685 | 0.0045 |
| Usual walking pace | IL_12_P70 | rs71361173 | 0.1105 | 0.0238 | 3.57E-06 | -0.0013246 | 0.00182845 | 0.47 |
| Usual walking pace | IL_12_P70 | rs72831623 | 0.1929 | 0.0367 | 1.51E-07 | 0.00722133 | 0.00300381 | 0.016 |
| Usual walking pace | IL_12_P70 | rs782107 | 0.0765 | 0.0156 | 9.13E-07 | -0.0018977 | 0.00127433 | 0.14 |
| Usual walking pace | IL_12_P70 | rs9472183 | -0.1006 | 0.0157 | 1.38E-10 | 0.00161951 | 0.00132477 | 0.22 |
| Usual walking pace | IL_13 | rs10995615 | -0.1591 | 0.0341 | 3.12E-06 | 0.00119616 | 0.00161069 | 0.46 |
| Usual walking pace | IL_13 | rs117795020 | -0.3584 | 0.0716 | 5.48E-07 | 4.32E-04 | 0.00472572 | 0.93 |
| Usual walking pace | IL_13 | rs12623722 | -0.1189 | 0.0257 | 3.61E-06 | 0.00253894 | 0.00139112 | 0.068 |
| Usual walking pace | IL_13 | rs139083458 | 0.9995 | 0.211 | 2.17E-06 | 0.00556721 | 0.00483917 | 0.25 |
| Usual walking pace | IL_13 | rs27949 | -0.1144 | 0.025 | 4.83E-06 | -0.0020737 | 0.00136102 | 0.13 |
| Usual walking pace | IL_13 | rs6799107 | -0.1472 | 0.0299 | 8.66E-07 | 0.00240672 | 0.00155774 | 0.12 |
| Usual walking pace | IL_13 | rs6921438 | -0.4139 | 0.0242 | 1.28E-65 | -9.08E-04 | 0.00127181 | 0.48 |
| Usual walking pace | IL_13 | rs7073807 | 0.1618 | 0.0354 | 4.77E-06 | 3.59E-04 | 0.00201993 | 0.86 |
| Usual walking pace | IL_13 | rs75383097 | -0.5369 | 0.116 | 3.70E-06 | -0.0021786 | 0.00544085 | 0.69 |
| Usual walking pace | IL_13 | rs76339001 | -0.4375 | 0.0886 | 7.92E-07 | -0.0058653 | 0.00398662 | 0.14 |
| Usual walking pace | IL_13 | rs77955971 | 0.4408 | 0.0868 | 3.76E-07 | -0.0024397 | 0.00404676 | 0.55 |
| Usual walking pace | IL_16 | rs116135478 | 0.8296 | 0.1637 | 4.05E-07 | -0.0036481 | 0.00446127 | 0.41 |
| Usual walking pace | IL_16 | rs117217798 | -0.2064 | 0.044 | 2.77E-06 | 0.00344811 | 0.00257037 | 0.18 |
| Usual walking pace | IL_16 | rs117916513 | -0.4713 | 0.0982 | 1.61E-06 | -0.0028069 | 0.00565724 | 0.62 |
| Usual walking pace | IL_16 | rs1255143 | 0.1387 | 0.0241 | 8.53E-09 | 0.0023803 | 0.00127701 | 0.062 |
| Usual walking pace | IL_16 | rs144691581 | 0.4929 | 0.0958 | 2.67E-07 | 0.00881979 | 0.00541319 | 0.1 |
| Usual walking pace | IL_16 | rs1801020 | 0.1678 | 0.0271 | 5.63E-10 | 0.00262382 | 0.00146095 | 0.072 |
| Usual walking pace | IL_16 | rs4253283 | 0.1506 | 0.026 | 7.22E-09 | -4.20E-04 | 0.0013614 | 0.760001 |
| Usual walking pace | IL_16 | rs4778636 | -0.7286 | 0.063 | 6.21E-31 | -0.0011832 | 0.00218989 | 0.59 |
| Usual walking pace | IL_17 | rs11640734 | -0.115 | 0.024 | 1.61E-06 | 1.28E-04 | 0.00204145 | 0.95 |
| Usual walking pace | IL_17 | rs117556572 | -0.5256 | 0.1097 | 1.66E-06 | -0.0053319 | 0.00484378 | 0.27 |
| Usual walking pace | IL_17 | rs12735700 | -0.0943 | 0.0206 | 4.50E-06 | 1.51E-04 | 0.00147172 | 0.92 |
| Usual walking pace | IL_17 | rs148562661 | 0.2161 | 0.0434 | 6.37E-07 | 0.00173438 | 0.00509836 | 0.73 |
| Usual walking pace | IL_17 | rs149738638 | -0.1553 | 0.0337 | 4.11E-06 | 0.00267505 | 0.00244729 | 0.27 |
| Usual walking pace | IL_17 | rs1530455 | 0.1088 | 0.0173 | 3.29E-10 | -0.001531 | 0.00130298 | 0.24 |
| Usual walking pace | IL_17 | rs17106604 | 0.1119 | 0.0225 | 6.23E-07 | -0.002432 | 0.00201267 | 0.23 |
| Usual walking pace | IL_17 | rs17282552 | -0.2026 | 0.0403 | 4.88E-07 | -0.0044154 | 0.00358004 | 0.22 |
| Usual walking pace | IL_17 | rs78296352 | 0.2949 | 0.0645 | 4.81E-06 | -0.0013505 | 0.00326992 | 0.68 |
| Usual walking pace | IL_17 | rs9568764 | 0.0825 | 0.018 | 4.68E-06 | -5.64E-04 | 0.00141558 | 0.69 |
| Usual walking pace | IL_18 | rs116383510 | -0.5412 | 0.1052 | 2.70E-07 | 0.003934 | 0.00556472 | 0.48 |
| Usual walking pace | IL_18 | rs117266781 | 0.7051 | 0.1436 | 9.18E-07 | -0.0052378 | 0.00576599 | 0.36 |
| Usual walking pace | IL_18 | rs12420140 | -0.2479 | 0.0261 | 1.95E-21 | 0.00114987 | 0.00140341 | 0.41 |
| Usual walking pace | IL_18 | rs143370787 | -0.3447 | 0.066 | 1.75E-07 | 0.0037725 | 0.00229286 | 0.1 |
| Usual walking pace | IL_18 | rs17229943 | -0.3076 | 0.0463 | 3.06E-11 | 0.00439062 | 0.00288931 | 0.13 |
| Usual walking pace | IL_18 | rs1979967 | 0.14 | 0.0285 | 8.72E-07 | -0.0021584 | 0.00150967 | 0.15 |
| Usual walking pace | IL_18 | rs385076 | -0.2472 | 0.0247 | 1.56E-23 | -0.0016968 | 0.001331 | 0.2 |
| Usual walking pace | IL_18 | rs4482818 | 0.1233 | 0.0243 | 4.11E-07 | -0.0041535 | 0.00131185 | 0.0015 |
| Usual walking pace | IL_18 | rs610473 | 0.1274 | 0.0242 | 1.43E-07 | -0.0026035 | 0.00132276 | 0.049 |
| Usual walking pace | IL_18 | rs7444013 | -0.5318 | 0.0955 | 2.59E-08 | -0.0076035 | 0.00559477 | 0.17 |
| Usual walking pace | IL_18 | rs78623212 | 0.8322 | 0.1676 | 6.82E-07 | 0.00296429 | 0.004113 | 0.47 |
| Usual walking pace | IL_18 | rs78716465 | 0.3173 | 0.0679 | 2.98E-06 | 0.00303576 | 0.00332247 | 0.36 |
| Usual walking pace | IL_1B | rs115242021 | 0.2795 | 0.0553 | 4.25E-07 | 0.00114843 | 0.00276156 | 0.68 |
| Usual walking pace | IL_1B | rs143319329 | 0.4357 | 0.093 | 2.84E-06 | 0.00348047 | 0.00399984 | 0.38 |
| Usual walking pace | IL_1B | rs61335305 | 0.4333 | 0.0928 | 3.02E-06 | 0.00699819 | 0.00474218 | 0.14 |
| Usual walking pace | IL_1B | rs62015704 | 0.1786 | 0.0372 | 1.62E-06 | 0.00218404 | 0.00192095 | 0.26 |
| Usual walking pace | IL_1RA | rs1054402 | 0.1325 | 0.0269 | 8.20E-07 | -0.0024271 | 0.00147092 | 0.099001 |
| Usual walking pace | IL_1RA | rs11627423 | 0.1178 | 0.0246 | 1.65E-06 | -0.0017424 | 0.00129242 | 0.18 |
| Usual walking pace | IL_1RA | rs11869294 | -0.2286 | 0.047 | 1.13E-06 | 5.51E-04 | 0.00368281 | 0.88 |
| Usual walking pace | IL_1RA | rs187166731 | -0.2424 | 0.0504 | 1.55E-06 | 0.00869784 | 0.00933419 | 0.35 |
| Usual walking pace | IL_1RA | rs4441609 | 0.1056 | 0.0231 | 4.75E-06 | -0.0015358 | 0.00130532 | 0.24 |
| Usual walking pace | IL_1RA | rs61335305 | 0.4315 | 0.0904 | 1.81E-06 | 0.00699819 | 0.00474218 | 0.14 |
| Usual walking pace | IL_1RA | rs6699436 | -0.1858 | 0.0404 | 4.37E-06 | 0.00380707 | 0.0018153 | 0.036 |
| Usual walking pace | IL_2 | rs13412535 | 0.174 | 0.0331 | 1.45E-07 | -0.0012967 | 0.00150183 | 0.39 |
| Usual walking pace | IL_2 | rs16836080 | 0.1158 | 0.0253 | 4.84E-06 | 0.00163638 | 0.00140909 | 0.25 |
| Usual walking pace | IL_2 | rs2690020 | 0.1158 | 0.0245 | 2.27E-06 | 6.62E-04 | 0.00130175 | 0.61 |
| Usual walking pace | IL_2 | rs4634519 | -0.1249 | 0.0268 | 3.18E-06 | -0.0023601 | 0.00140213 | 0.092001 |
| Usual walking pace | IL_2 | rs61335305 | 0.4439 | 0.0913 | 1.16E-06 | 0.00699819 | 0.00474218 | 0.14 |
| Usual walking pace | IL_2 | rs62124990 | -0.7013 | 0.149 | 2.50E-06 | -0.0050022 | 0.00400744 | 0.21 |
| Usual walking pace | IL_2 | rs7615304 | -0.1139 | 0.024 | 2.16E-06 | 0.00188485 | 0.00132055 | 0.15 |
| Usual walking pace | IL_2RA | rs11241559 | -0.124 | 0.0264 | 2.75E-06 | -0.0020464 | 0.00149569 | 0.17 |
| Usual walking pace | IL_2RA | rs117244812 | -0.7187 | 0.1493 | 1.47E-06 | 3.43E-04 | 0.00597548 | 0.95 |
| Usual walking pace | IL_2RA | rs12722497 | 0.6287 | 0.0482 | 7.98E-39 | -1.08E-04 | 0.00224478 | 0.96 |
| Usual walking pace | IL_2RA | rs12799226 | -0.1285 | 0.0277 | 3.56E-06 | -0.0013784 | 0.00156325 | 0.38 |
| Usual walking pace | IL_2RA | rs185231391 | 0.8568 | 0.1803 | 2.00E-06 | 0.0099954 | 0.00577849 | 0.084 |
| Usual walking pace | IL_2RA | rs28441585 | 0.1269 | 0.0271 | 2.93E-06 | 0.00491297 | 0.00151936 | 0.0012 |
| Usual walking pace | IL_2RA | rs4733117 | 0.1439 | 0.0291 | 7.91E-07 | -0.0025971 | 0.00180968 | 0.15 |
| Usual walking pace | IL_4 | rs10512267 | -0.0824 | 0.016 | 2.73E-07 | -2.93E-04 | 0.0013519 | 0.83 |
| Usual walking pace | IL_4 | rs116705532 | -0.4675 | 0.0978 | 1.73E-06 | -0.0080112 | 0.00499063 | 0.11 |
| Usual walking pace | IL_4 | rs117146485 | -0.2856 | 0.0625 | 4.95E-06 | -0.005439 | 0.00638671 | 0.39 |
| Usual walking pace | IL_4 | rs12238729 | 0.5271 | 0.1096 | 1.51E-06 | -0.0089672 | 0.00547543 | 0.1 |
| Usual walking pace | IL_4 | rs13106889 | -0.1186 | 0.0224 | 1.22E-07 | -1.24E-04 | 0.00176006 | 0.94 |
| Usual walking pace | IL_4 | rs17713451 | 0.1255 | 0.0252 | 6.41E-07 | -0.0013253 | 0.00183962 | 0.47 |
| Usual walking pace | IL_4 | rs2073438 | 0.0847 | 0.0183 | 3.73E-06 | -2.06E-04 | 0.0013927 | 0.88 |
| Usual walking pace | IL_4 | rs58202480 | -0.0767 | 0.0166 | 3.59E-06 | 0.00247739 | 0.0015827 | 0.12 |
| Usual walking pace | IL_4 | rs6765768 | 0.0796 | 0.0167 | 1.85E-06 | 0.00133971 | 0.00130954 | 0.31 |
| Usual walking pace | IL_4 | rs73023729 | -0.1796 | 0.0365 | 8.56E-07 | -0.0089137 | 0.00505582 | 0.077999 |
| Usual walking pace | IL_4 | rs7613691 | 0.1787 | 0.0382 | 2.96E-06 | -9.40E-04 | 0.00260428 | 0.719999 |
| Usual walking pace | IL_4 | rs79597994 | -0.5855 | 0.1271 | 4.06E-06 | 0.0056071 | 0.00407484 | 0.17 |
| Usual walking pace | IL_4 | rs9508291 | -0.168 | 0.0358 | 2.67E-06 | 0.00152414 | 0.00254451 | 0.55 |
| Usual walking pace | IL_4 | rs9941733 | 0.1156 | 0.0229 | 4.33E-07 | -0.0014415 | 0.00169076 | 0.39 |
| Usual walking pace | IL_5 | rs11680908 | 0.2593 | 0.0552 | 2.62E-06 | -0.0012166 | 0.00258373 | 0.64 |
| Usual walking pace | IL_5 | rs148634917 | -0.517 | 0.1087 | 1.97E-06 | -0.0014564 | 0.00474151 | 0.760001 |
| Usual walking pace | IL_5 | rs28793375 | 0.1697 | 0.0362 | 2.75E-06 | -5.49E-04 | 0.00188986 | 0.77 |
| Usual walking pace | IL_5 | rs6737109 | 0.1135 | 0.0246 | 3.81E-06 | -0.0031004 | 0.00128174 | 0.016 |
| Usual walking pace | IL_5 | rs72831687 | -0.5337 | 0.1104 | 1.32E-06 | 0.00100567 | 0.0070435 | 0.89 |
| Usual walking pace | IL_5 | rs73040130 | 0.2745 | 0.0525 | 1.71E-07 | -0.0038742 | 0.0026643 | 0.15 |
| Usual walking pace | IL_5 | rs74811276 | 0.217 | 0.0471 | 4.08E-06 | 0.00134146 | 0.00236548 | 0.57 |
| Usual walking pace | IL_5 | rs9472168 | 0.1568 | 0.0253 | 5.42E-10 | 3.70E-04 | 0.00128642 | 0.77 |
| Usual walking pace | IL_6 | rs10752777 | 0.1083 | 0.0235 | 4.17E-06 | 2.48E-04 | 0.00233772 | 0.92 |
| Usual walking pace | IL_6 | rs10982213 | -0.0849 | 0.0176 | 1.35E-06 | -9.70E-05 | 0.00151139 | 0.95 |
| Usual walking pace | IL_6 | rs113098456 | -0.1553 | 0.0339 | 4.64E-06 | -0.0024085 | 0.00258712 | 0.35 |
| Usual walking pace | IL_6 | rs1333040 | 0.0747 | 0.0157 | 1.99E-06 | -0.0013549 | 0.00128317 | 0.29 |
| Usual walking pace | IL_6 | rs13412535 | -0.1186 | 0.0214 | 3.14E-08 | -0.0012967 | 0.00150183 | 0.39 |
| Usual walking pace | IL_6 | rs2404476 | 0.0734 | 0.0156 | 2.68E-06 | -0.0026421 | 0.00126712 | 0.037 |
| Usual walking pace | IL_6 | rs4684700 | -0.0747 | 0.0162 | 3.91E-06 | -0.0041576 | 0.00128953 | 0.0013 |
| Usual walking pace | IL_6 | rs72831623 | 0.197 | 0.0369 | 9.29E-08 | 0.00722133 | 0.00300381 | 0.016 |
| Usual walking pace | IL_6 | rs73273528 | 0.268 | 0.0553 | 1.25E-06 | 0.00469701 | 0.00345075 | 0.17 |
| Usual walking pace | IL_6 | rs75101555 | -0.3625 | 0.0781 | 3.44E-06 | -0.0027479 | 0.00402176 | 0.49 |
| Usual walking pace | IL_6 | rs76856708 | 0.336 | 0.0697 | 1.43E-06 | -1.39E-04 | 0.00337057 | 0.97 |
| Usual walking pace | IL_7 | rs10196226 | 0.1538 | 0.0327 | 2.50E-06 | -0.0026748 | 0.0019573 | 0.17 |
| Usual walking pace | IL_7 | rs115215018 | 0.5985 | 0.1308 | 4.76E-06 | -0.0046667 | 0.00480591 | 0.33 |
| Usual walking pace | IL_7 | rs117509142 | -0.3213 | 0.0684 | 2.60E-06 | -0.0040631 | 0.00338637 | 0.23 |
| Usual walking pace | IL_7 | rs141425475 | -0.4801 | 0.1018 | 2.39E-06 | -0.0048036 | 0.00402084 | 0.23 |
| Usual walking pace | IL_7 | rs17091524 | 0.5092 | 0.1015 | 5.24E-07 | 0.00130894 | 0.00371779 | 0.719999 |
| Usual walking pace | IL_7 | rs1958987 | 0.1261 | 0.0263 | 1.60E-06 | -6.16E-04 | 0.00135385 | 0.649999 |
| Usual walking pace | IL_7 | rs28793375 | 0.1644 | 0.036 | 4.87E-06 | -5.49E-04 | 0.00188986 | 0.77 |
| Usual walking pace | IL_7 | rs62006410 | -0.1492 | 0.0302 | 7.59E-07 | 0.00249562 | 0.00159484 | 0.12 |
| Usual walking pace | IL_7 | rs6921438 | -0.3204 | 0.0246 | 8.71E-39 | -9.08E-04 | 0.00127181 | 0.48 |
| Usual walking pace | IL_7 | rs77981494 | -0.5201 | 0.1055 | 8.23E-07 | -0.004679 | 0.0044489 | 0.29 |
| Usual walking pace | IL_8 | rs116726256 | -0.2247 | 0.0489 | 4.26E-06 | 0.00442779 | 0.0050361 | 0.38 |
| Usual walking pace | IL_8 | rs12075 | 0.1148 | 0.0235 | 9.97E-07 | 0.00190711 | 0.0012804 | 0.14 |
| Usual walking pace | IL_8 | rs12438669 | -0.1182 | 0.0252 | 2.60E-06 | 3.79E-04 | 0.00132066 | 0.77 |
| Usual walking pace | IL_8 | rs141926526 | -0.6221 | 0.1308 | 1.96E-06 | -0.0043518 | 0.0032538 | 0.18 |
| Usual walking pace | IL_8 | rs2673604 | -0.118 | 0.0254 | 3.29E-06 | -7.44E-04 | 0.0014074 | 0.6 |
| Usual walking pace | IL_8 | rs3786107 | 0.2463 | 0.0517 | 1.94E-06 | -0.0017169 | 0.00279476 | 0.54 |
| Usual walking pace | IL_8 | rs75840288 | 0.5125 | 0.1121 | 4.85E-06 | -0.0015142 | 0.00331074 | 0.649999 |
| Usual walking pace | IL_9 | rs117807175 | -0.5225 | 0.1106 | 2.33E-06 | -0.0085901 | 0.00389 | 0.027 |
| Usual walking pace | IL_9 | rs3736858 | -0.1351 | 0.0291 | 3.37E-06 | -0.0026033 | 0.00176946 | 0.14 |
| Usual walking pace | IL_9 | rs41294750 | 0.3442 | 0.0736 | 2.92E-06 | -0.0052836 | 0.00379575 | 0.16 |
| Usual walking pace | IL_9 | rs73443903 | 0.2162 | 0.046 | 2.57E-06 | -0.0010437 | 0.00219762 | 0.630001 |
| Usual walking pace | IL_9 | rs76963786 | -0.2856 | 0.0556 | 2.78E-07 | -8.20E-04 | 0.00218794 | 0.709999 |
| Usual walking pace | IP_10 | rs113831257 | 0.3639 | 0.0641 | 1.39E-08 | -0.0030138 | 0.00325997 | 0.36 |
| Usual walking pace | IP_10 | rs143799975 | -0.7551 | 0.1638 | 4.01E-06 | 0.00955285 | 0.00585917 | 0.1 |
| Usual walking pace | IP_10 | rs34383175 | -0.3196 | 0.0653 | 9.90E-07 | -0.0039134 | 0.00341323 | 0.25 |
| Usual walking pace | IP_10 | rs397816 | 0.1211 | 0.0248 | 1.03E-06 | 7.42E-05 | 0.00128184 | 0.95 |
| Usual walking pace | IP_10 | rs4862111 | 0.1448 | 0.0317 | 4.84E-06 | -0.0053179 | 0.00169462 | 0.0017 |
| Usual walking pace | IP_10 | rs6707974 | 0.1574 | 0.0337 | 3.03E-06 | 0.00251788 | 0.00172501 | 0.14 |
| Usual walking pace | IP_10 | rs75970138 | -0.4845 | 0.1037 | 2.99E-06 | 0.0139531 | 0.00711737 | 0.05 |
| Usual walking pace | IP_10 | rs7645625 | -0.1116 | 0.0236 | 2.19E-06 | -0.001736 | 0.00128356 | 0.18 |
| Usual walking pace | IP_10 | rs79848609 | 0.2514 | 0.0535 | 2.64E-06 | -0.0021873 | 0.00305158 | 0.47 |
| Usual walking pace | IP_10 | rs8112909 | -0.139 | 0.0297 | 2.96E-06 | -0.0014628 | 0.00157986 | 0.35 |
| Usual walking pace | IP_10 | rs9450351 | -0.2651 | 0.0488 | 5.48E-08 | 0.00339472 | 0.00257125 | 0.19 |
| Usual walking pace | MCP_1_MCAF | rs10744620 | 0.0783 | 0.0161 | 1.12E-06 | -0.0014939 | 0.00131086 | 0.25 |
| Usual walking pace | MCP_1_MCAF | rs111995966 | 0.1428 | 0.0309 | 3.79E-06 | 0.00325473 | 0.00471184 | 0.49 |
| Usual walking pace | MCP_1_MCAF | rs12073356 | -0.1436 | 0.031 | 3.49E-06 | 0.00268381 | 0.00259099 | 0.3 |
| Usual walking pace | MCP_1_MCAF | rs12075 | 0.2186 | 0.0154 | 1.36E-45 | 0.00190711 | 0.0012804 | 0.14 |
| Usual walking pace | MCP_1_MCAF | rs143815843 | -0.2049 | 0.0447 | 4.61E-06 | -0.0113553 | 0.00691763 | 0.1 |
| Usual walking pace | MCP_1_MCAF | rs146522229 | -0.5942 | 0.1161 | 3.09E-07 | 0.00230625 | 0.00575162 | 0.69 |
| Usual walking pace | MCP_1_MCAF | rs2036297 | 0.1182 | 0.016 | 1.30E-13 | 0.0010441 | 0.00133126 | 0.43 |
| Usual walking pace | MCP_1_MCAF | rs2288370 | -0.1036 | 0.0162 | 1.56E-10 | -2.53E-04 | 0.00131136 | 0.85 |
| Usual walking pace | MCP_1_MCAF | rs56212190 | 0.1799 | 0.0372 | 1.32E-06 | -0.0026556 | 0.0030785 | 0.39 |
| Usual walking pace | MCP_1_MCAF | rs7033586 | -0.22 | 0.0467 | 2.43E-06 | -0.016393 | 0.0132446 | 0.22 |
| Usual walking pace | MCP_1_MCAF | rs7197349 | 0.0971 | 0.0206 | 2.40E-06 | 9.33E-04 | 0.00192377 | 0.630001 |
| Usual walking pace | MCP_1_MCAF | rs7632755 | 0.2984 | 0.0315 | 2.79E-21 | 4.25E-04 | 0.0024848 | 0.86 |
| Usual walking pace | MCP_1_MCAF | rs9317045 | 0.1157 | 0.0235 | 8.43E-07 | -4.80E-04 | 0.00172738 | 0.780001 |
| Usual walking pace | MCP_3 | rs10892381 | 0.2432 | 0.0473 | 2.69E-07 | 0.00237221 | 0.00140579 | 0.092001 |
| Usual walking pace | MCP_3 | rs117286643 | 0.6934 | 0.1474 | 2.54E-06 | -0.0051613 | 0.00500961 | 0.3 |
| Usual walking pace | MCP_3 | rs2838065 | -0.221 | 0.0479 | 3.92E-06 | 0.00309463 | 0.00161711 | 0.056 |
| Usual walking pace | MCP_3 | rs28394764 | 0.597 | 0.1282 | 3.19E-06 | -0.0020094 | 0.00290625 | 0.49 |
| Usual walking pace | MCP_3 | rs3129806 | -0.1975 | 0.0433 | 4.98E-06 | 0.00137517 | 0.00131159 | 0.29 |
| Usual walking pace | MCP_3 | rs62492260 | -0.2802 | 0.0578 | 1.23E-06 | -0.0041107 | 0.00189216 | 0.03 |
| Usual walking pace | MIF | rs113218956 | -0.8789 | 0.1876 | 2.82E-06 | -0.0095555 | 0.00963865 | 0.32 |
| Usual walking pace | MIF | rs11551183 | 0.3666 | 0.0795 | 4.00E-06 | -0.005261 | 0.00336862 | 0.12 |
| Usual walking pace | MIF | rs12594190 | 0.1321 | 0.0266 | 6.85E-07 | -6.21E-04 | 0.00152 | 0.68 |
| Usual walking pace | MIF | rs13142904 | -0.2232 | 0.0425 | 1.47E-07 | 0.00314183 | 0.00253498 | 0.22 |
| Usual walking pace | MIF | rs141009259 | -0.6194 | 0.1285 | 1.44E-06 | -0.0042213 | 0.00609934 | 0.49 |
| Usual walking pace | MIF | rs2294689 | -0.1338 | 0.0287 | 3.04E-06 | -0.0039594 | 0.00287318 | 0.17 |
| Usual walking pace | MIF | rs2330634 | 0.1549 | 0.0249 | 4.57E-10 | 8.26E-04 | 0.00129356 | 0.52 |
| Usual walking pace | MIF | rs35890933 | 0.1676 | 0.0365 | 4.46E-06 | -0.0011148 | 0.00158451 | 0.48 |
| Usual walking pace | MIF | rs3814097 | -0.1163 | 0.0251 | 3.55E-06 | 0.00128397 | 0.00130723 | 0.33 |
| Usual walking pace | MIF | rs78098071 | -0.4583 | 0.0915 | 5.51E-07 | 0.0156084 | 0.00589755 | 0.0081 |
| Usual walking pace | MIG | rs111607343 | -0.5235 | 0.1119 | 2.93E-06 | 0.00220004 | 0.00365963 | 0.55 |
| Usual walking pace | MIG | rs11177248 | 0.3157 | 0.0667 | 2.22E-06 | -0.0016136 | 0.00258493 | 0.53 |
| Usual walking pace | MIG | rs117831247 | -0.8819 | 0.173 | 3.45E-07 | 0.0037083 | 0.005894 | 0.53 |
| Usual walking pace | MIG | rs13143163 | 0.2735 | 0.0582 | 2.62E-06 | 0.00319715 | 0.00270031 | 0.24 |
| Usual walking pace | MIG | rs139010077 | 0.4337 | 0.0943 | 4.19E-06 | 0.00102274 | 0.00554175 | 0.85 |
| Usual walking pace | MIG | rs1796086 | -0.2172 | 0.04 | 5.62E-08 | 0.00148574 | 0.00224571 | 0.51 |
| Usual walking pace | MIG | rs62562991 | 0.6239 | 0.1259 | 7.24E-07 | -0.0062693 | 0.00464706 | 0.18 |
| Usual walking pace | MIG | rs6679677 | 0.1628 | 0.0327 | 6.51E-07 | -0.0049512 | 0.0020984 | 0.018 |
| Usual walking pace | MIG | rs77086208 | 0.327 | 0.0694 | 2.50E-06 | -0.0039695 | 0.0048778 | 0.42 |
| Usual walking pace | MIG | rs816960 | -0.1179 | 0.0242 | 1.15E-06 | -0.0010172 | 0.00154475 | 0.51 |
| Usual walking pace | MIP_1A | rs12690897 | 0.1215 | 0.026 | 3.07E-06 | 5.57E-04 | 0.00145109 | 0.7 |
| Usual walking pace | MIP_1A | rs57786342 | 0.139 | 0.0283 | 8.91E-07 | -1.28E-04 | 0.00157684 | 0.94 |
| Usual walking pace | MIP_1A | rs60198979 | -0.2154 | 0.0455 | 2.22E-06 | 3.04E-04 | 0.00232536 | 0.9 |
| Usual walking pace | MIP_1A | rs6900267 | -0.2472 | 0.0515 | 1.60E-06 | -0.0052167 | 0.00296454 | 0.077999 |
| Usual walking pace | MIP_1B | rs113010081 | -0.5799 | 0.0236 | 1.57E-133 | 0.00177793 | 0.00200296 | 0.37 |
| Usual walking pace | MIP_1B | rs113877493 | -0.607 | 0.0217 | 3.67E-172 | -4.46E-04 | 0.00232708 | 0.85 |
| Usual walking pace | MIP_1B | rs116237296 | 0.5284 | 0.1115 | 2.15E-06 | -8.08E-04 | 0.00611657 | 0.89 |
| Usual walking pace | MIP_1B | rs117453826 | -0.5907 | 0.0591 | 1.53E-23 | -0.0043203 | 0.00501452 | 0.39 |
| Usual walking pace | MIP_1B | rs117657747 | 0.2089 | 0.0453 | 4.01E-06 | -0.0026086 | 0.00262469 | 0.32 |
| Usual walking pace | MIP_1B | rs141102180 | 0.3298 | 0.0392 | 3.75E-17 | 6.37E-06 | 0.0049224 | 1 |
| Usual walking pace | MIP_1B | rs1437220 | 0.1437 | 0.0315 | 4.92E-06 | 0.0034983 | 0.0028482 | 0.22 |
| Usual walking pace | MIP_1B | rs1564708 | -0.1697 | 0.0187 | 1.29E-19 | -0.0028359 | 0.00160192 | 0.077 |
| Usual walking pace | MIP_1B | rs17138331 | -0.1434 | 0.0295 | 1.13E-06 | 3.50E-05 | 0.00196587 | 0.99 |
| Usual walking pace | MIP_1B | rs2411161 | 0.1719 | 0.0365 | 2.55E-06 | -0.0010349 | 0.00279483 | 0.709999 |
| Usual walking pace | MIP_1B | rs281748 | -0.0794 | 0.0171 | 3.28E-06 | -6.91E-04 | 0.0013646 | 0.61 |
| Usual walking pace | MIP_1B | rs3760440 | 0.1242 | 0.0162 | 1.73E-14 | 0.00124829 | 0.00133911 | 0.35 |
| Usual walking pace | MIP_1B | rs6908843 | 0.0997 | 0.0209 | 1.78E-06 | -2.39E-04 | 0.00164219 | 0.88 |
| Usual walking pace | MIP_1B | rs72791296 | 0.2364 | 0.0466 | 3.97E-07 | -0.0013965 | 0.00307189 | 0.649999 |
| Usual walking pace | MIP_1B | rs72799710 | -0.1037 | 0.0217 | 1.79E-06 | 8.54E-04 | 0.00163609 | 0.6 |
| Usual walking pace | MIP_1B | rs76356863 | -0.3456 | 0.0667 | 2.22E-07 | 3.90E-05 | 0.00372815 | 0.99 |
| Usual walking pace | MIP_1B | rs76582507 | 0.3259 | 0.0676 | 1.42E-06 | -0.0054081 | 0.0049701 | 0.28 |
| Usual walking pace | MIP_1B | rs76776296 | 0.313 | 0.0598 | 1.63E-07 | 0.00188182 | 0.00328552 | 0.57 |
| Usual walking pace | MIP_1B | rs79068918 | 0.2674 | 0.0271 | 5.54E-23 | -1.79E-04 | 0.00212909 | 0.93 |
| Usual walking pace | M_CSF | rs116274860 | 0.8262 | 0.1739 | 2.03E-06 | -0.0055014 | 0.00502086 | 0.27 |
| Usual walking pace | M_CSF | rs117867915 | 0.5224 | 0.1096 | 1.87E-06 | 0.00359569 | 0.00642282 | 0.58 |
| Usual walking pace | M_CSF | rs11963606 | -0.5353 | 0.117 | 4.73E-06 | -0.0043636 | 0.00504619 | 0.39 |
| Usual walking pace | M_CSF | rs12962919 | 0.3025 | 0.0659 | 4.39E-06 | -0.0012138 | 0.00209311 | 0.56 |
| Usual walking pace | M_CSF | rs34089869 | 0.2194 | 0.0462 | 2.08E-06 | -3.36E-04 | 0.00200982 | 0.87 |
| Usual walking pace | M_CSF | rs4269021 | -0.2459 | 0.0504 | 1.05E-06 | 7.22E-04 | 0.00183994 | 0.69 |
| Usual walking pace | M_CSF | rs56367447 | -0.4878 | 0.0876 | 2.57E-08 | 8.74E-04 | 0.00330365 | 0.79 |
| Usual walking pace | M_CSF | rs62294910 | 0.3472 | 0.0687 | 4.38E-07 | -6.23E-04 | 0.00276744 | 0.82 |
| Usual walking pace | M_CSF | rs72723242 | -0.4969 | 0.1083 | 4.43E-06 | 0.00161707 | 0.00275119 | 0.56 |
| Usual walking pace | M_CSF | rs78296352 | 0.522 | 0.111 | 2.58E-06 | -0.0013505 | 0.00326992 | 0.68 |
| Usual walking pace | M_CSF | rs9387100 | -0.135 | 0.029 | 3.34E-06 | -1.90E-04 | 0.00133884 | 0.89 |
| Usual walking pace | PDGF_BB | rs11247305 | -0.1687 | 0.0364 | 3.47E-06 | 3.66E-05 | 0.0034423 | 0.99 |
| Usual walking pace | PDGF_BB | rs116445074 | 0.2869 | 0.0587 | 1.02E-06 | 0.00307888 | 0.00522862 | 0.56 |
| Usual walking pace | PDGF_BB | rs11766649 | 0.0902 | 0.0196 | 3.96E-06 | 6.18E-04 | 0.00144674 | 0.67 |
| Usual walking pace | PDGF_BB | rs12289510 | -0.0772 | 0.0158 | 1.00E-06 | -0.0014996 | 0.00126951 | 0.24 |
| Usual walking pace | PDGF_BB | rs13037046 | -0.0948 | 0.0206 | 3.96E-06 | 8.93E-04 | 0.00150164 | 0.55 |
| Usual walking pace | PDGF_BB | rs13412535 | 0.3317 | 0.0214 | 2.89E-54 | -0.0012967 | 0.00150183 | 0.39 |
| Usual walking pace | PDGF_BB | rs2324229 | 0.0884 | 0.0161 | 4.02E-08 | 6.98E-04 | 0.00131052 | 0.59 |
| Usual walking pace | PDGF_BB | rs35859699 | -0.3854 | 0.0838 | 4.22E-06 | 0.00147393 | 0.00424977 | 0.73 |
| Usual walking pace | PDGF_BB | rs4965869 | 0.1843 | 0.0181 | 2.22E-24 | 7.37E-04 | 0.00142922 | 0.61 |
| Usual walking pace | PDGF_BB | rs72777070 | -0.1048 | 0.02 | 1.56E-07 | -0.0013795 | 0.00164098 | 0.4 |
| Usual walking pace | PDGF_BB | rs73162807 | -0.2313 | 0.0499 | 3.55E-06 | -0.0012511 | 0.00439204 | 0.780001 |
| Usual walking pace | PDGF_BB | rs9936075 | -0.0767 | 0.0163 | 2.68E-06 | 0.00240343 | 0.00133832 | 0.073 |
| Usual walking pace | PDGF_BB | rs9941733 | 0.1165 | 0.0227 | 3.02E-07 | -0.0014415 | 0.00169076 | 0.39 |
| Usual walking pace | RANTES | rs112072646 | 0.4209 | 0.0859 | 9.62E-07 | -0.0030138 | 0.00385422 | 0.43 |
| Usual walking pace | RANTES | rs147509526 | -0.3558 | 0.0715 | 6.57E-07 | -0.0025142 | 0.00532042 | 0.64 |
| Usual walking pace | RANTES | rs2251660 | 0.1831 | 0.0356 | 2.69E-07 | -0.0020433 | 0.00177768 | 0.25 |
| Usual walking pace | RANTES | rs2731672 | -0.1242 | 0.0272 | 4.83E-06 | 0.00236837 | 0.00145 | 0.1 |
| Usual walking pace | RANTES | rs62438851 | -0.1904 | 0.0413 | 4.01E-06 | -0.0016744 | 0.00185319 | 0.37 |
| Usual walking pace | RANTES | rs7000423 | -0.1314 | 0.0252 | 1.85E-07 | -0.0019936 | 0.00135394 | 0.14 |
| Usual walking pace | RANTES | rs7170339 | -0.4283 | 0.0904 | 2.19E-06 | 0.00244567 | 0.00462362 | 0.6 |
| Usual walking pace | RANTES | rs72793342 | -0.1505 | 0.0307 | 9.08E-07 | 0.0017007 | 0.0015834 | 0.28 |
| Usual walking pace | RANTES | rs74472919 | 0.3547 | 0.06 | 3.35E-09 | 0.00389253 | 0.00364838 | 0.29 |
| Usual walking pace | RANTES | rs9675798 | -0.2583 | 0.0552 | 2.89E-06 | 8.20E-04 | 0.00336087 | 0.81 |
| Usual walking pace | SCF | rs113127926 | 0.1974 | 0.0418 | 2.34E-06 | 2.13E-04 | 0.00252113 | 0.93 |
| Usual walking pace | SCF | rs13412535 | -0.1065 | 0.0213 | 5.59E-07 | -0.0012967 | 0.00150183 | 0.39 |
| Usual walking pace | SCF | rs1557570 | 0.1172 | 0.0169 | 4.13E-12 | -9.39E-04 | 0.00134747 | 0.49 |
| Usual walking pace | SCF | rs1568119 | -0.5946 | 0.1129 | 1.37E-07 | -0.0062738 | 0.00657891 | 0.34 |
| Usual walking pace | SCF | rs4841899 | -0.1002 | 0.0178 | 1.67E-08 | 4.27E-04 | 0.00135451 | 0.75 |
| Usual walking pace | SCF | rs635634 | -0.1035 | 0.0191 | 5.71E-08 | -0.0017334 | 0.00163493 | 0.29 |
| Usual walking pace | SCF | rs7039247 | 0.079 | 0.0168 | 2.46E-06 | 7.89E-04 | 0.00134611 | 0.56 |
| Usual walking pace | SCF | rs72678285 | 0.1062 | 0.0231 | 4.43E-06 | -0.0011007 | 0.0017418 | 0.53 |
| Usual walking pace | SCF | rs78369473 | -0.2256 | 0.0484 | 3.14E-06 | -0.0051016 | 0.0037618 | 0.18 |
| Usual walking pace | SCF | rs78666213 | -0.2845 | 0.0574 | 7.15E-07 | 0.00469543 | 0.00361647 | 0.19 |
| Usual walking pace | SCF | rs80271436 | -0.2393 | 0.0484 | 7.49E-07 | 0.00124982 | 0.00291533 | 0.67 |
| Usual walking pace | SCGF_B | rs112346514 | -0.3261 | 0.0703 | 3.54E-06 | 0.00520807 | 0.00376336 | 0.17 |
| Usual walking pace | SCGF_B | rs1149926 | -0.3458 | 0.0749 | 3.92E-06 | -0.0034982 | 0.00394118 | 0.37 |
| Usual walking pace | SCGF_B | rs116924815 | 0.6046 | 0.0737 | 2.25E-16 | -0.001297 | 0.00380124 | 0.73 |
| Usual walking pace | SCGF_B | rs117716477 | 0.8242 | 0.084 | 1.03E-22 | 0.00577602 | 0.00540661 | 0.29 |
| Usual walking pace | SCGF_B | rs12480722 | 0.1654 | 0.0353 | 2.81E-06 | 0.00280513 | 0.00200033 | 0.16 |
| Usual walking pace | SCGF_B | rs13287050 | -0.121 | 0.0263 | 4.12E-06 | -9.22E-05 | 0.00135715 | 0.95 |
| Usual walking pace | SCGF_B | rs139413256 | -0.5174 | 0.1076 | 1.53E-06 | -0.0021333 | 0.00334799 | 0.52 |
| Usual walking pace | SCGF_B | rs143829871 | -0.1866 | 0.0399 | 2.85E-06 | -0.006525 | 0.00262051 | 0.013 |
| Usual walking pace | SCGF_B | rs149009264 | 0.4551 | 0.0985 | 3.79E-06 | -0.0073632 | 0.00560703 | 0.19 |
| Usual walking pace | SCGF_B | rs150733161 | -0.5255 | 0.112 | 2.69E-06 | 0.0163819 | 0.00542038 | 0.0025 |
| Usual walking pace | SCGF_B | rs151194174 | 0.4536 | 0.0941 | 1.45E-06 | 4.88E-04 | 0.00446555 | 0.91 |
| Usual walking pace | SCGF_B | rs17876031 | -0.1496 | 0.0254 | 3.67E-09 | 0.00214064 | 0.00135882 | 0.12 |
| Usual walking pace | SCGF_B | rs264157 | 0.1079 | 0.0233 | 3.69E-06 | 0.00101629 | 0.00128583 | 0.43 |
| Usual walking pace | SCGF_B | rs34911860 | -0.3674 | 0.0787 | 3.00E-06 | 0.00248308 | 0.00654994 | 0.7 |
| Usual walking pace | SCGF_B | rs4656185 | 0.2103 | 0.0254 | 1.29E-16 | -8.48E-04 | 0.00134956 | 0.53 |
| Usual walking pace | SCGF_B | rs77954165 | 0.2631 | 0.0562 | 2.87E-06 | -0.0044076 | 0.00219894 | 0.045 |
| Usual walking pace | SCGF_B | rs7815967 | 0.1325 | 0.0288 | 4.37E-06 | -0.0058435 | 0.00196558 | 0.0029 |
| Usual walking pace | SCGF_B | rs78217154 | 0.3942 | 0.0861 | 4.72E-06 | 0.00593049 | 0.00445561 | 0.18 |
| Usual walking pace | SDF_1A | rs10013755 | 0.5188 | 0.0995 | 1.85E-07 | 0.00226625 | 0.00463139 | 0.62 |
| Usual walking pace | SDF_1A | rs149893336 | -0.494 | 0.1082 | 4.93E-06 | -0.0071696 | 0.00479841 | 0.14 |
| Usual walking pace | SDF_1A | rs1600396 | -0.0933 | 0.0204 | 4.94E-06 | 0.00362726 | 0.001457 | 0.013 |
| Usual walking pace | SDF_1A | rs3988298 | -0.1263 | 0.0266 | 2.12E-06 | 0.00105377 | 0.0019325 | 0.59 |
| Usual walking pace | SDF_1A | rs62194947 | -0.0852 | 0.0185 | 4.27E-06 | -0.0018876 | 0.00143807 | 0.19 |
| Usual walking pace | SDF_1A | rs78037609 | -0.6261 | 0.1334 | 2.67E-06 | 0.00570517 | 0.00743212 | 0.44 |
| Usual walking pace | SDF_1A | rs78883416 | -0.0871 | 0.0182 | 1.76E-06 | -0.001691 | 0.00134933 | 0.21 |
| Usual walking pace | TNF_A | rs10834997 | -0.123 | 0.0256 | 1.53E-06 | -5.30E-04 | 0.00137035 | 0.7 |
| Usual walking pace | TNF_A | rs111332265 | -0.3678 | 0.0745 | 7.91E-07 | -0.0001698 | 0.00275154 | 0.95 |
| Usual walking pace | TNF_A | rs115669577 | 0.981 | 0.1994 | 8.63E-07 | -0.0032945 | 0.00663583 | 0.62 |
| Usual walking pace | TNF_A | rs7256693 | -0.1841 | 0.04 | 4.11E-06 | -2.82E-04 | 0.00184271 | 0.88 |
| Usual walking pace | TNF_A | rs79105320 | 0.5573 | 0.1177 | 2.21E-06 | 0.00498397 | 0.0049265 | 0.31 |
| Usual walking pace | TNF_B | rs10925040 | 0.1738 | 0.0372 | 2.93E-06 | -1.10E-04 | 0.00131263 | 0.93 |
| Usual walking pace | TNF_B | rs75240021 | 0.3713 | 0.0772 | 1.49E-06 | -0.0021966 | 0.00242304 | 0.36 |
| Usual walking pace | TNF_B | rs753274 | -0.1725 | 0.037 | 3.14E-06 | 2.78E-04 | 0.00127378 | 0.83 |
| Usual walking pace | TNF_B | rs7629875 | 0.3841 | 0.0774 | 6.90E-07 | -0.0032192 | 0.00287099 | 0.26 |
| Usual walking pace | TNF_B | rs78296352 | 1.2028 | 0.1366 | 1.28E-18 | -0.0013505 | 0.00326992 | 0.68 |
| Usual walking pace | TRAIL | rs13278062 | 0.08 | 0.0157 | 3.33E-07 | 0.00304853 | 0.00126651 | 0.016 |
| Usual walking pace | TRAIL | rs138987090 | -0.7264 | 0.0749 | 2.97E-22 | -0.004172 | 0.00782742 | 0.59 |
| Usual walking pace | TRAIL | rs148051545 | -0.4211 | 0.0843 | 5.84E-07 | 0.00627794 | 0.00408655 | 0.12 |
| Usual walking pace | TRAIL | rs17434886 | -0.0918 | 0.0199 | 4.20E-06 | -0.0011877 | 0.00173359 | 0.49 |
| Usual walking pace | TRAIL | rs193112415 | -1.0456 | 0.062 | 1.01E-63 | 0.00248054 | 0.00467426 | 0.6 |
| Usual walking pace | TRAIL | rs28431810 | -0.1216 | 0.0252 | 1.41E-06 | 0.00670871 | 0.00680916 | 0.32 |
| Usual walking pace | TRAIL | rs28521641 | -0.7004 | 0.0445 | 7.79E-56 | -0.0014139 | 0.00352717 | 0.69 |
| Usual walking pace | TRAIL | rs550057 | -0.0783 | 0.0169 | 3.71E-06 | -7.81E-04 | 0.0014509 | 0.59 |
| Usual walking pace | TRAIL | rs57396456 | -0.5641 | 0.0516 | 7.71E-28 | -0.0033212 | 0.00364883 | 0.36 |
| Usual walking pace | TRAIL | rs62093514 | 1.0459 | 0.0549 | 5.80E-81 | 0.00146312 | 0.00395867 | 0.709999 |
| Usual walking pace | TRAIL | rs72899452 | 0.1223 | 0.0264 | 3.75E-06 | 0.00174949 | 0.00256573 | 0.5 |
| Usual walking pace | TRAIL | rs73039026 | -0.3098 | 0.0634 | 1.02E-06 | -0.0063396 | 0.00584294 | 0.28 |
| Usual walking pace | TRAIL | rs747324 | -0.0826 | 0.0178 | 3.34E-06 | -5.97E-04 | 0.00135673 | 0.66 |
| Usual walking pace | TRAIL | rs74778900 | 0.5791 | 0.0531 | 9.90E-28 | 0.00224722 | 0.0052892 | 0.67 |
| Usual walking pace | TRAIL | rs75928541 | 0.2784 | 0.0591 | 2.44E-06 | 0.00658896 | 0.00459922 | 0.15 |
| Usual walking pace | TRAIL | rs79287178 | -0.4304 | 0.042 | 1.17E-24 | 0.00250186 | 0.00382461 | 0.51 |
| Usual walking pace | VEGF | rs10411345 | -0.1041 | 0.0218 | 1.73E-06 | 0.00258374 | 0.00166602 | 0.12 |
| Usual walking pace | VEGF | rs10934631 | -0.1132 | 0.0244 | 3.61E-06 | -8.98E-04 | 0.00158291 | 0.57 |
| Usual walking pace | VEGF | rs10967186 | 0.0899 | 0.0169 | 1.09E-07 | 3.00E-04 | 0.0012673 | 0.81 |
| Usual walking pace | VEGF | rs12456390 | -0.0818 | 0.0179 | 4.88E-06 | 5.85E-05 | 0.0013585 | 0.97 |
| Usual walking pace | VEGF | rs13209117 | 0.1253 | 0.02 | 3.70E-10 | -8.83E-04 | 0.00142606 | 0.54 |
| Usual walking pace | VEGF | rs143479231 | -0.2628 | 0.0489 | 7.90E-08 | 0.0016093 | 0.00489102 | 0.74 |
| Usual walking pace | VEGF | rs3108686 | -0.7967 | 0.1702 | 2.86E-06 | -0.0059703 | 0.00527279 | 0.26 |
| Usual walking pace | VEGF | rs4082730 | 0.2455 | 0.0533 | 4.12E-06 | -0.0042678 | 0.00345688 | 0.22 |
| Usual walking pace | VEGF | rs6921438 | -0.4866 | 0.0174 | 4.11E-172 | -9.08E-04 | 0.00127181 | 0.48 |
| Usual walking pace | VEGF | rs7030781 | 0.1403 | 0.0172 | 3.45E-16 | -0.0021197 | 0.001295 | 0.1 |
| Usual walking pace | VEGF | rs73418463 | -0.2498 | 0.0521 | 1.61E-06 | 0.00622353 | 0.00280998 | 0.027 |
| Usual walking pace | VEGF | rs73872715 | -0.6079 | 0.1299 | 2.86E-06 | -0.0024189 | 0.00527518 | 0.649999 |
| Usual walking pace | VEGF | rs8045833 | 0.103 | 0.0211 | 1.01E-06 | 3.00E-04 | 0.00142619 | 0.83 |
| Usual walking pace | VEGF | rs9472183 | -0.1264 | 0.017 | 9.54E-14 | 0.00161951 | 0.00132477 | 0.22 |
